# Structure and Drug Binding of the SARS-CoV-2 Envelope Protein in Phospholipid Bilayers

**DOI:** 10.21203/rs.3.rs-77124/v1

**Published:** 2020-09-24

**Authors:** Venkata S. Mandala, Matthew J. McKay, Alexander A. Shcherbakov, Aurelio J. Dregni, Antonios Kolocouris, Mei Hong

**Affiliations:** 1 Department of Chemistry, Massachusetts Institute of Technology, Cambridge, Massachusetts, 02139, USA; 2 Department of Pharmaceutical Chemistry, National and Kapodistrian University of Athens, Panepistimioupolis Zografou, Athens 15771, Greece

## Abstract

Severe acute respiratory syndrome coronavirus 2 (SARS-CoV-2) is the causative agent of the ongoing COVID-19 pandemic. Successful development of vaccines and antivirals against SARS-CoV-2 requires a comprehensive understanding of the essential proteins of the virus. The envelope (E) protein of SARS-CoV-2 assembles into a cation-selective channel that mediates virus budding, release, and host inflammation response. E blockage reduces virus pathogenicity while E deletion attenuates the virus. Here we report the 2.4 Å structure and drug-binding site of E’s transmembrane (TM) domain, determined using solid-state nuclear magnetic resonance (NMR) spectroscopy. In lipid bilayers that mimic the endoplasmic reticulum Golgi intermediate compartment (ERGIC) membrane, ETM forms a five-helix bundle surrounding a narrow central pore. The middle of the TM segment is distorted from the ideal α-helical geometry due to three regularly spaced phenylalanine residues, which stack within each helix and between neighboring helices. These aromatic interactions, together with interhelical Val and Leu interdigitation, cause a dehydrated pore compared to the viroporins of influenza and HIV viruses. Hexamethylene amiloride and amantadine bind shallowly to polar residues at the N-terminal lumen, while acidic pH affects the C-terminal conformation. These results indicate that SARS-CoV-2 E forms a structurally robust but bipartite channel whose N- and C-terminal halves can interact with drugs, ions and other viral and host proteins semi-independently. This structure establishes the atomic basis for designing E inhibitors as antiviral drugs against SARS-CoV-2.

Nine months into the COVID-19 pandemic, no vaccine or antiviral drugs are yet available against SARS-CoV-2, owing to a lack of knowledge about the detailed structures and functions of key virus proteins. The RNA genome of SARS-CoV-2 encodes three membrane proteins: the spike protein, which binds the cell-surface receptor to mediate virus entry; the membrane protein, which contributes to virus assembly and budding ^1^; and the envelope protein (Fig. 1a). E is a 75-residue viroporin (Fig. 1b) that forms a cation-selective channel across the ERGIC membrane ^2,3^. The protein mediates the budding and release of progeny viruses ^4^ and is involved in activation of the host inflammasome ^5^. E’s channel activity is blocked by hexamethylene amiloride (HMA) ^6^ and amantadine (AMT) ^7^, the latter also known to inhibit the viroporins of influenza A virus and HIV-1 ^8,9^. E inhibition reduces viral pathogenicity while E deletion gives rise to attenuated viruses in some coronaviruses ^10–12^, suggesting that E is a potential antiviral and vaccine target.

Despite its importance to SARS-CoV-2 pathogenesis, E’s structure, particularly for the ion-conducting TM domain (residues 8–38) ^2,13^, has been elusive. Sedimentation equilibrium and gel electrophoresis data indicate that the TM domain assembles into a pentamer in detergent micelles such as SDS and perfluorooctanoic acid ^3,14,15^, but the membrane topology is debated. X-ray scattering of DMPC-bound ETM of SARS-CoV found Phe23 electron density in the lipid headgroup region, suggesting that the TM domain crosses the lipid bilayer twice as a short hairpin ^16^, thus juxtaposing the N-terminal Glu8 with the C-terminal Arg38. In contrast, solution NMR studies of E bound to DPC ^10^, SDS ^15^, and LMPG ^17^ micelles indicate a single-span TM helix; however, the pore-facing residues and the pentameric assembly have not been well established.

To avoid potential structural distortion caused by detergents, we determined the ETM structure in phospholipid bilayers using solid-state NMR spectroscopy. We reconstituted ETM into an ERGIC-mimetic lipid membrane containing phosphocholine, phosphoethanolamine, phosphatidylinositol, phosphoserine and cholesterol. For comparison, we also incorporated the protein into dimyristoylphosphocholine (DMPC) : dimyristoyl-phosphoglycerol (DMPG) membranes. ETM was expressed in *E. coli* using a His_6_-SUMO fusion tag (Fig. S1) and purified by nickel affinity column chromatography and reverse-phase HPLC. One-dimensional (1D) ^13^C and ^15^N NMR spectra of the protein in ERGIC and DMPC : DMPG membranes show temperature-insensitive high intensities (Fig. S2a, b), indicating that the protein is immobilized in the lipid membranes at ambient temperature. Two-dimensional (2D) ^15^N-^13^C and ^13^C-^13^C correlation spectra show well-resolved peaks for most residues (Fig. 1c, d) with ^13^C and ^15^N linewidths of 0.5 ppm and 0.9 ppm, indicating that the protein conformation is highly homogeneous. We assigned the chemical shifts using 3D correlation NMR experiments (Fig. S3a, Table S1). These chemical shifts indicate that residues 14–34 form the α-helical core of the TM domain (Fig. S3b, c). Comparison of spectra between the two membranes and at different temperatures (Fig. S2d–f) indicate that the N-terminal segment (residues E8-I13) is dynamic at high temperature but has α-helical propensity, while the C-terminal segment (residues T35-R38) is more rigid but displays temperature-dependent conformations. Acidic pH perturbed the chemical shifts of C-terminal residues L34-R38 (Fig. S4), supporting the conclusion that the C-terminal segment is conformationally plastic.

The temperature insensitivity of the protein spectra suggests that ETM is oligomerized in lipid bilayers. To determine the oligomeric structure, we prepared two mixed labeled protein samples to measure intermolecular distances. An equimolar ^13^C-labeled protein mixed with 4-^19^F-Phe labeled protein (Fig. S1e) was used to measure intermolecular ^13^C-^19^F distances using the REDOR technique ^18^ (Fig. 2a). ETM contains three regularly spaced phenylalanine (Phe) residues, Phe20, Phe23 and Phe26, at the center of the TM segment. 1D and 2D ^13^C NMR spectra were measured without and with ^19^F pulses. The resulting difference spectra show the signals of carbons that are in close proximity to a fluorinated Phe on a neighboring helix (Fig. 2b, Fig. S5a–c). As expected, residues V17 to L31 are affected by 4-^19^F-Phe, while residues I13 to S16 and A36 to R38 show no REDOR dephasing. Moreover, the three Phe’s display two resolved ^19^F chemical shifts, indicating that one of the residues has a distinct sidechain conformation. A 2D ^13^C-^19^F correlation spectrum (Fig. 2c) shows a cross peak between the −118 ppm ^19^F signal and A22 Cβ, indicating that this −118 ppm peak is due to either F20 or F23. The −113 ppm ^19^F peak shows strong cross peaks with aromatic and numerous aliphatic ^13^C chemical shifts. Since F20 and F26 are too far away from each other to form intermolecular contacts, the −118 ppm ^19^F peak must be assigned to F20, while F23 and F26 resonate at −113 ppm. To constrain the interhelical packing at the two termini of the TM domain, we prepared a ^13^C and ^15^N mixed labeled sample, and measured 2D NHHC correlation spectra, which exhibit ^15^N-^13^C correlation peaks that are exclusively intermolecular (Fig. 2d). These experiments together yielded 35 interhelical ^13^C-^19^F distance restraints and 52 interhelical ^15^N-^13^C correlations, which are crucial for determining the oligomeric structure of ETM.

To further constrain the E channel architecture, we measured the water accessibilities of different residues using water-edited 2D ^15^N-^13^C correlation experiments (Fig. 2e, Fig. S5d) ^19,20^. Water ^1^H magnetization transfer is the highest to the N-terminal residues, the least to the central residues L17 to A32, and moderate to the C terminus (Fig. 2f). Thus, the hydration gradient of the protein is primarily along the bilayer normal. The preferential hydration of the N-terminus is especially manifested by the high water-transferred intensity of L19 compared to T30, despite favorable chemical exchange to the Thr sidechain. For the dehydrated center of the TM domain, L28 and V25 show higher hydration than their neighboring residues, suggesting that these residues face the pore. A complementary lipid-edited experiment (Fig. 2g) showed much higher intensities for the Phe sidechain carbons than the corresponding water-transferred intensities, indicating that the Phe’s are more lipid-facing. The ERGIC-bound ETM shows two-fold lower water accessibility than the closed state of influenza BM2 at the same neutral pH (Fig. 2f).

We calculated the structure of ETM using the measured 56 (ϕ, ψ) torsion angles, 87 interhelical distance restraints (Tables S2, S3), and 196 intrahelical ^13^C-^13^C contacts obtained from 250 ms ^13^C spin diffusion spectra (Fig. S6) ^21^. We disambiguated the direction of interhelical contacts from one helix to the two neighboring helices by considering the pore- versus lipid-facing positions of the residues, the helical distortion between F20 and F23 (Fig. S3b), and the interhelical ^13^C-^19^F Phe-Phe contacts (Fig. S7e). The lowest-energy structure ensemble, with a heavy-atom RMSD of 2.4 Å (Table 1), shows a long and tight five-helix bundle with a vertical length of ~35 Å for residues V14-L34. The channel diameter varies from 11 Å to 14 Å, based on the Cα-Cα distances between helices *i* and *i*+2 for pore-facing residues (Fig. 3a). The helical bundle is primarily lef-thanded, although a minor conformer of right-handed bundle is also seen. Each helix is tilted by an angle of about 10° from the bilayer normal (Fig. 3b); however, this orientation is not uniform, because the helix is not ideal, and exhibits a significant rotation angle change, or twist, between residues F20-F23 ^10,17^. The pore of the channel is occupied by mostly hydrophobic residues, including N15, L18, L21, V25, L28, A32 and T35 (Fig. 3b–d, Fig. S8a), explaining the poor hydration of the protein. The N-terminus pore is constricted by N15, which forms interhelical sidechain hydrogen bonds (Fig. 3g) ^22^. Mutation of N15, as well as V25, is known to abolish cation conduction ^13^. The helix-helix interface is stabilized by aromatic stacking of F23 and F26 (Fig. 3e, g) and van der Waals packing among methyl-rich residues such as the V29-L31-I33 triad (Fig. 3f). These extensive hydrophobic interactions create a tighter helical bundle than the influenza viroporin BM2 and the HIV-1 viroporin Vpu (Fig. S8b).

To investigate how the E pentamer interacts with drugs, we measured the chemical shifts of the protein in the presence of HMA and AMT. At a drug : protein molar ratio of 4 : 1, HMA caused significant chemical shift perturbations (CSPs) to N-terminal residues, including T9, G10, T11, I13 and S16, followed by modest CSPs for the C-terminal A36 and L37 (Fig. 4a–c). This trend is consistent with the micelle data ^10,17^, but the CSPs in bilayers are much larger than in micelles, with the N-terminal ^9^TGT^11^ triplet giving CSPs of 0.35–0.70 ppm. Moreover, in bilayers CSPs were observed at only 4-fold drug excess, while in micelles CSPs were observed at higher drug excesses of 10 to 31-fold ^10,17^. The higher sensitivity to drug in lipid bilayers suggests that the bilayer-bound protein conformation is more native. Docking based on these CSPs found that HMA intercalates shallowly into the N-terminal lumen with a distribution of orientations (Fig. 4d, Fig. S9), suggesting a dynamic binding mode where HMA exchanges between multiple helices and inhibits cation conduction by steric occlusion of the pore. Within the ensemble of docked structures, more HMA molecules point the guanidinium into the pore and the hexamethylene ring to the lipid headgroups than the reverse orientation. AMT caused smaller CSPs than HMA (Fig. 4c, Fig. S10a, b), but the site of binding remains at the N-terminus. Using the 3-^19^F probe on the adamantane, we measured protein-drug proximities using ^13^C-^19^F REDOR. The spectra showed modest dephasing for the N-terminal N15 and C-terminal I33 (Fig. S10c–e), in qualitative agreement with the observed CSPs. The larger CSPs of HMA than AMT are consistent with the micromolar EC_50_ reported for the HCoV-229E E protein ^6^ compared to the millimolar binding affinities of AMT to SARS-CoV-2 E ^7^.

Which structural features of this ETM pentamer might be responsible for cation conduction? The N-terminal part of ETM contains a conserved (E/D/R)_1_x(G/A)_3_xxhh(N/Q)_8_ motif (Fig. 1b), where h is a hydrophobic residue. The most exposed residue, E8, belongs to a dynamic N-terminus whose residues (e.g. T9 and G10) manifest intensities only at high temperature (Fig. S2d–f). The E8 sidechain carboxyl is deprotonated at neutral pH and protonated at acidic pH, as seen in the ^13^C chemical shifts (Fig. S2c). We hypothesize that the protonation equilibria of this loose ring of E8 quintet, together with the anionic lipids in the ERGIC membrane, may regulate the ion selectivity of ETM at the channel entrance. A ring of negatively charged Glu residues has been observed as selectivity filters in the hexameric Ca^2+^-selective Orai channels ^23^ and designed K^+^ channels ^24^. The third residue of the motif, G10, is conserved among coronaviruses to be small and flexible, thus permitting N-terminus motion. The last residue of the motif, N15, is conserved to be either Asn or Gln, whose polar sidechains can coordinate ions as well as forming interhelical hydrogen bonds to stabilize the channel ^22^. At the C-terminal end, the conserved small residues A32 and T35 provide an open cavity for ions. In contrast to these small polar residues, the central portion of the TM domain contains four layers of hydrophobic residues, L18, L21, V25 and L28, which narrow the pore radius to ~2 Å (Fig. 3d). This narrow pore can only permit a single file of water molecules or ions, thus partially dehydrating any ions that move through the pore. Thus, the structure shown here may represent the closed state of SARS-CoV-2 E, while the open state may have a larger and more hydrated pore. We note, however, that narrow pores with multiple hydrophobic layers have been observed in ion channels, including the tetrameric K^+^ channel TMEM175 ^25^ and the pentameric bestrophin channels ^26,27^. Thus, it is possible to achieve charge stabilization and ion selectivity in such a hydrophobic environment, although the detailed mechanisms remain to be understood.

The bilayer-bound structure of SARS-CoV-2 E has similarities as well as differences from the micelle-bound structure ^10,17^. In micelles, ETM helix also displays a kink and the N-terminus is similarly disordered, but the handedness of the helical bundle and the identity of pore-facing residues vary with the detergent. For example, in LMPG micelles, F26 and T30 point to the lumen rather than lipids. Thus, the membrane-mimetic environment appears to influence E’s oligomeric structure. Compared to influenza and HIV-1 viroporins, the SARS-CoV-2 E helical bundle is tighter and more rigid. AM2 and BM2’s TM domains have a higher percentage of polar residues such as His and Ser. As a result, M2 forms wider and more hydrated pores (Fig. S8b) ^9,28^. The HIV-1 Vpu TM domain has a similarly high percentage of hydrophobic residues as SARS-CoV-2 E, but forms a shorter (~20 Å vertical length) pentameric helical bundle with more tilted helices (~20°) ^29,30^. The E helical bundle is more immobilized than M2 and Vpu ^31^, and does not undergo whole-body fast uniaxial rotation at high temperatures in DMPX membranes (Fig. S2). This immobilization suggest that the protein may interact extensively with lipids. Finally, the helix distortion at F20-F23 may cause the two halves of E’s TM domain to respond independently to environmental factors such as pH, membrane composition ^16^, and other viral and host proteins.

This membrane-bound ETM structure suggests that small-molecule E inhibitors should bind with high affinity to both the acidic E8 and the polar N15 in order to occlude the N-terminal entrance of the protein. The membrane topology of SARS-CoV-2 E is now recognized to be N_lumen_ – C_cyto_ based on antibody-detected selective permeabilization assays ^32^ and glycosylation data ^33^. This orientation would prime the protein to conduct Ca^2+^ out of the ERGIC lumen to activate the host inflammasome ^5^. Thus, small-molecule drugs should ideally be targeted and delivered to the ERGIC and Golgi of host cells to maximally inhibit SARS-CoV-2 E ^34^.

## Methods

### Cloning of recombinant ETM(8–38)

The gene encoding the full-length SARS-CoV-2/Wuhan-Hu-1 envelope (E) protein (NCBI reference sequence YP_009724392.1, residues 1–75) was purchased from Genewiz. The gene encoding the TM domain (residues 8–38, ETGTLIVNSVLLFLAFVVFLLVTLAILTALR) was isolated using PCR and cloned into a Champion pET-SUMO plasmid (Invitrogen). The plasmid was transfected into *E. coli* BL21 (DE3) cells (Invitrogen) to express the SUMO-ETM fusion protein containing an N-terminal His_6_ tag (Fig. S1a). The construct’s DNA sequence was verified by Sanger sequencing (Genewiz).

### Expression and purification of ^13^C, ^15^N-labeled ETM

A glycerol cell swab stored at −70°C was used to start a 10 mL LB culture containing 50 μg/mL kanamycin. The starter culture was used to inoculate 2 L of LB media. Cells were grown at 37°C until an OD_600_ of 0.6–0.8 and were harvested by centrifugation for 10 minutes at 20°C and 4,400x g. These LB cells were resuspended in 1 L of M9 media (pH 7.8, 48 mM Na_2_HPO_4_, 22 mM KH_2_PO_4_, 8.6 mM NaCl, 4 mM MgSO_4_, 0.2 mM CaCl_2_, 50 mg kanamycin) containing 1 g/L ^15^N-NH_4_Cl. The cells were incubated in M9 media for 30 min at 18°C, then 1 g/L U-^13^C glucose dissolved in 5 mL sterile H_2_O and 3 mL 100x MEM vitamins were added. The cells were grown for another 30 min, then protein expression was induced by addition of 0.4 mM isopropyl β-D-1-thiogalactopyranoside (IPTG) along with 2 g/L U-^13^C glucose in 10 mL sterile H_2_O. Additional IPTG was added after 1 hour to bring the final concentration to 0.8 mM. Protein expression proceeded overnight for 16 hours at 18°C, reaching an OD_600_ of 2.5.

The cells were spun down at 4°C and 5,000 rpm for 10 min and resuspended in 35 mL Lysis Buffer I (pH 8.0, 50 mM Tris-HCl, 100 mM NaCl, 1.0% Triton X-100, 0.5 mg/mL lysozyme, 10 μL benozonase nuclease, 1 mM Mg^2+^, 10 mM imidazole). Cells were lysed at 4°C by sonication (5 sec on and 5 sec off) for 1 hour using a probe sonicator. The soluble fraction of the cell lysate was separated from the inclusion bodies by centrifugation at 17,000x g for 20 min at 4°C. The supernatant was loaded onto a gravity-flow chromatography column containing ~6 mL nickel affinity resin (Profinity IMAC, BioRad) pre-equilibrated with Lysis Buffer I. The fractions were bound to the resin for 1 hour by gentle rocking at 4°C. The column was washed with 50 mL of Wash Buffer I (pH 8.0, 50 mM Tris-HCl, 100 mM NaCl, 0.1% n-dodecyl-B-D-maltoside (DDM), 30 mM imidazole). SUMO-ETM was eluted with 10–15 mL Elution Buffer (pH 8.0, 50 mM Tris-HCl, 100 mM NaCl, 0.1% DDM, 250 mM Imidazole) (Fig. S1b). The eluted protein was diluted to one-third of the original concentration by adding twice the elution volume of Dilution Buffer (pH 8.0, 50 mM Tris-HCl, 100 mM NaCl, 0.1% DDM) to reduce the imidazole concentration before protease cleavage. Approximately 20% of the protein was also found in the insoluble membrane and inclusion body fraction. To purify this fraction, the pelleted mass was resuspended in Lysis Buffer II (Lysis buffer I with added 6 M urea) and rocked gently at 4°C overnight. Soluble protein was isolated by centrifugation at 17,000x g for 20 min at 4°C. Nickel affinity column chromatography proceeded as described above for the soluble fraction, except that Wash Buffer II (Wash Buffer I with added 3 M urea) was utilized in place of Wash Buffer I.

The purified SUMO-ETM fusion protein from both the soluble and inclusion body fractions were cleaved by addition of 1 : 10 (w/w) SUMO protease : SUMO-ETM and 5 mM tris(2-carboxyethyl)phosphine (TCEP) for 2 hours at room temperature with gentle rocking. The cleavage efficiency was assessed by analytical HPLC and was typically ~75%. ETM was purified using preparative RP-HPLC on a Varian ProStar 210 System using an Agilent C3 column (5 μm particle size, 21.2 mm × 150 mm). The protein was eluted using a linear gradient of 5–99% (9:1, acetonitrile : isopropanol) : water containing 0.1% trifluoroacetic acid over 35 minutes at a flow rate of 10 mL/min (Fig. S1c). The purified protein was dried down to a film under a stream of nitrogen gas and placed under vacuum overnight. The protein film was stored at −20°C. Typical yield of the purified protein was 10 mg/L of M9 media. Labeling efficiency was ~94% as estimated by MALDI mass spectrometry (Fig. S1d). U-^13^C-labeled ETM and U-^15^N-labeled ETM were expressed and purified following the same protocol, but substituting ^15^N-NH_4_Cl or ^13^C-glucose with unlabeled reagents.

### Expression of 4-^19^F-Phe fluorinated ETM

A glycerol cell swab was used to start a 10 mL LB culture containing 50 μg/mL kanamycin. The starter culture was then used to inoculate 2 L of M9 media (pH 7.8, 48 mM Na_2_HPO_4_, 22 mM KH_2_PO_4_, 8.6 mM NaCl, 4 mM MgSO_4_, 0.2 mM CaCl_2_, 50 mg kanamycin) containing 3 g/L unlabeled glucose and 1 g/L unlabeled NH_4_Cl. The cells were grown in M9 at 37°C for media for 8 hours until an OD_600_ of 0.5. The cells were collected by centrifugation at 4,400x g for 10 min at 20°C, then concentrated into a fresh 1 L M9 culture and incubated at 30°C for 60 min. Subsequently, 1.5 g/L of glyphosate was added to halt the pentose phosphate pathway for aromatic amino acid synthesis ^35^, followed by addition of 115 mg L-Trp, 115 mg L-Tyr and 400 mg of 4-^19^F-L-Phe to the culture. After 30 min, IPTG was added to a final concentration of 0.4 mM, and protein expression was allowed to proceed at 30°C for 5.5 hours. The cells were collected by centrifugation at 4,400x g for 10 min at 4°C. The pellet was stored at −70°C until purification. Cell lysis and protein purification followed the same protocol as outlined above, except that the ETM peak during preparative HPLC was collected in two fractions of approximately 1 min each. Fluorine incorporation in the two fractions was measured using MALDI mass spectrometry. The first fraction had a higher fluorine incorporation level of 83% for all three Phe residues labeled with ^19^F, indicating a per-residue labeling efficiency of 94% (Fig. S1e). Only this fraction was used to prepare the mixed ^13^C and ^19^F labeled protein for interhelical distance measurement. The final yield of the Phe-fluorinated ETM expression was 1.5 mg/L of M9 media. The protocol was originally tested using 100 mg/L of 4-^19^F-Phe, 1.0 g/L of glyphosate, 6 g/L unlabeled glucose and with expression at 18°C for 5.5 hours, which yielded a much lower per-residue labeling efficiency of ~35%.

### Membrane sample preparation

Eight proteins samples in two different lipid membranes were prepared for this study. Five membrane samples contained ^13^C, ^15^N-labeled ETM and one contained ^13^C-labeled ETM. Another sample contained a 1 : 1 mixture of ^13^C-labeled protein : ^15^N-labeled protein. The last sample contained a 1 : 1 mixture of ^13^C-labeled protein : 4-^19^F-Phe-labeled protein. Six of the eight samples were prepared in a pH 7.5 Tris buffer (20 mM Tris-HCl, 5 mM NaCl, 2 mM ethylenediaminetetraacetic acid (EDTA) and 0.2 mM NaN_3_). One sample was prepared in a pH 5 citrate buffer with calcium (20 mM Citrate, 5 mM CaCl_2_ and 0.2 mM NaN_3_), while the final sample was prepared in the same pH 5 citrate buffer without calcium chloride.

Chemical shift assignment and interhelical distance measurements were conducted on ETM bound to an ERGIC-mimetic membrane ^36,37^ containing 1-palmitoyl-2-oleoyl-glycero-3-phosphocholine (POPC), 1-palmitoyl-2-oleoyl-sn-glycero-3-phosphoethanolamine (POPE), bovine phosphatidylinositol (PI), 1-palmitoyl-2-oleoyl-sn-glycero-3-phospho-L-serine (POPS), and cholesterol (Chol). The POPC : POPE : PI : POPS : Chol molar ratios were 45 : 20 : 13 : 7 : 15. All lipids were purchased from Avanti Polar Lipids. The membrane has a protein : lipid molar ratio (P : L) of 1 : 20, and 2–4 mg ^13^C, ^15^N-labeled protein was used for most 2D and 3D correlation experiments. The intermolecular NHHC spectra were measured using a sample containing 4 mg each of ^13^C-labeled ETM and ^15^N-labeled ETM. This mixture was reconstituted into the ERGIC membrane at a P : L of 1 : 10 to increase the experimental sensitivity. ^13^C-^19^F REDOR experiments were conducted on 3.7 mg total of 1 : 1 mixed ^13^C-labeled and fluorinated ETM bound to the ERGIC membrane at P : L = 1 : 10.

To reconstitute ETM into lipid bilayers, we dissolved 2 mg protein in 1 mL trifluoroethanol (TFE) and mixed with appropriate amounts of lipids in 400 μL chloroform. For the HMA-bound sample, HMA was dissolved in TFE (1 mg/100 μL) and added to the protein-lipid mixture. The organic solvents were removed under a gentle stream of nitrogen gas, and the film was dried under vacuum at room temperature overnight. The proteoliposome film was resuspended in 3 mL of pH 7.5 sample buffer by vortexing and sonicating 2–3 times for 5 min until the suspension was homogenous. This was followed by 7 freeze-thaw cycles between a 42°C water bath and liquid nitrogen. The proteoliposomes were then pelleted using ultracentrifugation for 3 hours at 164,000x g and 4°C. The pellet was dried in a desiccator or under a gentle stream of nitrogen gas to a final hydration level of ~40% by mass and then packed into an appropriate MAS rotor using a benchtop centrifuge.

Drug binding to ETM was assessed in a “DMPX” membrane consisting of 1,2-dimyristoyl-sn-glycero-3-phosphocholine (DMPC) : 1,2-dimyristoyl-sn-glycero-3-phospho-(1’-rac-glycerol) (DMPG) at a 80% : 20% molar ratio. The mixture was chosen to maintain the same 20% anionic lipid fraction as the ERGIC membrane. A drug-free sample contained 2 mg of U-^13^C, ^15^N-labeled ETM bound to the membrane at a P : L of 1 : 20. The sample containing 5-(N,N-hexamethylene)-amiloride (HMA) was prepared using a protein : drug (P : D) molar ratio of 1 : 1, with HMA (0.2 mg) added during organic solution mixing. The same P : L of 1:20 as the apo sample was used. After initial spectra showed only small CSPs, we titrated an additional 0.6 mg of HMA in 6 μl dimethyl sulfoxide (DMSO) into the proteoliposome, giving a P : D of 1 : 4. The solubility of HMA in aqueous solutions was very low (< 0.1 mg/ml), necessitating the use of DMSO. 3-^19^F-amantadine (AMT) was titrated into the proteoliposome stepwise, from an initial P : D molar ratio of 1 : 1 to a final P : D of 1 : 8. The protein/lipid molar ratio of the sample is 1 : 15. The fluorinated AMT has high solubility in water, thus can be mixed with the membrane directly. For the ^13^C-^19^F REDOR experiments, the sample was packed in a 1.9 mm MAS rotor, while chemical shift measurements were conducted in a 3.2 mm MAS rotor on the 800 MHz spectrometer.

Chemical shift changes under acidic pH and with added calcium were assessed in the same “DMPX” membrane. The sample with 5 mM CaCl_2_ at pH 5 contained 2 mg of U-^13^C, ^15^N-labeled ETM bound to the membrane at a P : L of 1 : 20, while the sample without calcium contained 2 mg of U-^13^C-labeled ETM bound to the membrane at a P : L of 1 : 20.

## Synthesis of F-Amt

**Scheme S1:**
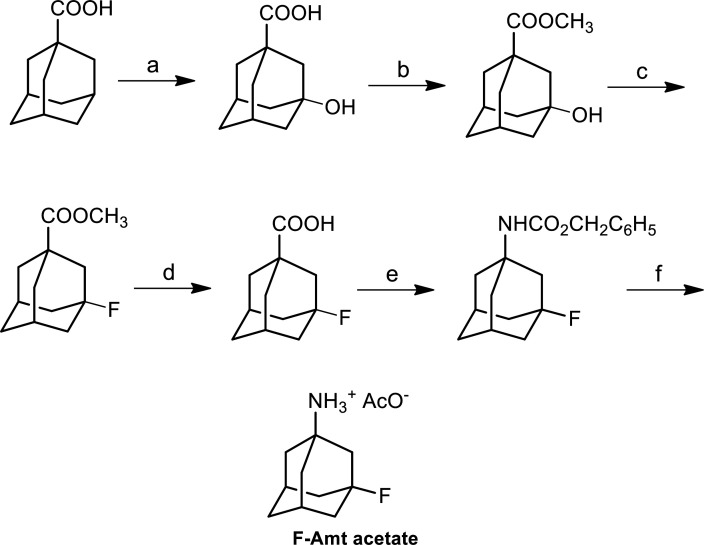


The synthetic protocol (Scheme S1) used for preparation of F-Amt was adapted from that described by Jasys and coworkers (Jasys, V. J.; Lombardo, F.; Appleton, T. A.; Bordner, J.; Ziliox, M.; Volkmann, R. A. Preparation of Fluoroadamantane Acids and Amines: Impact of Bridgehead Fluorine Substitution on the Solution-and Solid-State Properties of Functionalized Adamantanes. *J. Am. Chem. Soc.* **2000**, *122*, 466–473). Thus, the reaction between 1-adamantanecarboxylic acid and KMnO_4_ afforded 3-hydroxyadamantanecarboxylic acid which was transformed through its tetrabutylammonium salt to the corresponding methyl ester. The fluorination of the hydroxyester was accomplished through treatment with diethylaminosulfur trifluoride (DAST) at −50 °C. The 3-fluoroadamantane-1-amine acetate (F-Amt acetate) was obtained by treatment of 3-fluoroadamantanecarboxylic acid with diphenylphosphorylazide (DPPA) and subsequent hydrogenolysis of the resultant benzyl carbamate.

Reagents and Conditions: (a) KMnO_4_, KOH, 50 °C; (b) TBAHSO_4_, NaHCO_3_, CH_3_I, acetone, 48 h, r.t.; (c) DAST, CH_2_Cl_2_, 3 h, −50 °C → 60 °C; (d) NaOH, MeOH, THF, H_2_O, 24 h, r.t.; (e) DPPA, TEA, BnOH, benzene, 70 °C; (f) H_2_, 10 % Pd/C, AcOH.

3-fluoroadamantane-1-amine acetate (F-Amt acetate): ^1^H-NMR (phosphate buffer, pH 7, 10 % D_2_O, 500 MHz) *δ* (ppm) 1.60–1.69 (m, 2H, 6-H), 1.87 (s, 4H, 4,10-H), 1.89–1.98 (m, 7H, 8,9-H, CH_3_COO^−^), 2.09 (br d, 2H, 2-H), 2.48 (br s, 2H, 5,7-H); LC-MS (m/z) 170.3 (FC_10_H_14_NH_3_^+^). *Base:* mp 210 °C (EtOH-ether); ^1^H-NMR (CDCl_3_, 400 MHz) *δ* (ppm) 1.41 (br s, 2H, 6-H), 1.49 (br s, 2H, NH_2_), 1.51 (br s, 4H, 4,10-H), 1.74 (br d, 2H, *J* = 5.8 Hz, 2-H), 1.79 (br m, 4H, 8,9-H), 2.31 (br s, 2H, 5,7-H); ^13^C-NMR (CDCl_3_, 50 MHz) *δ* (ppm) 31.33, 31.55 (5,7-C), 34.67 (6-C), 41.44, 41.77 (4,10-C), 44.74 (8,9-C), 51.14, 51.47 (2-C, 3-C), 93.29 (d, *J*_C-F_ = 183.8 Hz, 1-C). Anal. Acetate (C_12_H_20_NO_2_F) (EtOH-Et_2_O). Calc. C: 62.86 H: 8.79. Found. C: 62.56 H: 8.99.

### Solid-state NMR experiments

Most solid-state NMR spectra were measured on a Bruker AVANCE NEO 900 MHz (21.1 T) spectrometer and an Avance II 800 MHz (18.8 T) spectrometer using 3.2 mm HCN probes. Intermolecular ^13^C-^19^F REDOR experiments were conducted on an Avance III HD 600 MHz (14.1 T) spectrometer using a 1.9 mm HFX probe. MAS frequencies were 11.8 kHz for all 900 MHz experiments and 14 kHz for the 800 and 600 MHz experiments. Radiofrequency (RF) field strengths on the 3.2 mm probes were 50–91 kHz for ^1^H, 50–63 kHz for ^13^C, and 33–42 kHz for ^15^N. RF field strengths on the 1.9 mm MAS probe were 83–130 kHz for ^1^H, 62.5 kHz for ^13^C, and 71 kHz for ^19^F. Sample temperatures are direct readings from the probe thermocouple, whereas actual sample temperatures are 5–15 K higher at the MAS frequencies employed. ^13^C chemical shifts are reported on the tetramethylsilane scale using the adamantane CH_2_ chemical shift at 38.48 ppm as an external standard. ^15^N chemical shifts are reported on the liquid ammonia scale using the N-acetylvaline peak at 122.00 ppm as an external standard.

2D ^13^C-^13^C correlation experiments were conducted using COmbined -Driven (CORD) mixing ^38^ for ^13^C spin diffusion. 2D and 3D ^15^N-^13^C correlation spectra, namely, NCACX, NCOCX, and CONCA ^39^, were measured on the 900 MHz spectrometer. These experiments used SPECtrally Induced Filtering In Combination with Cross Polarization (SPECIFIC-CP) ^40^ for heteronuclear polarization transfer. Water-edited 2D ^15^N-^13^Cα correlation spectra were measured under 11.8 kHz MAS ^19,20^ using ^1^H mixing times of 9 ms and 100 ms. 2D ^15^N-^13^C correlation spectra were measured using an out-and-back Transferred-Echo Double Resonance (TEDOR) pulse sequence on the 800 MHz NMR ^41^. Intermolecular 2D NHHC correlation spectra ^42^ were measured used 0.5 ms and 1 ms ^1^H-^1^H mixing. 1D and 2D ^13^C-^19^F REDOR experiments ^18,43,44^ were conducted to measure distances between 4-^19^F-Phe-labeled and ^13^C-labeled ETM, and to measure dipolar dephasing of ^13^C-labeled ETM by 3-^19^F-AMT. Additional specific parameters for the NMR experiments are given in Table S5.

### NMR spectral analysis

NMR spectra were processed in the TopSpin software while chemical shifts were assigned in Sparky ^45^. TALOS-N ^46^ was used to calculate (ϕ, ψ) torsion angles after converting the ^13^C chemical shifts to the DSS scale. Residue-specific chemical shift differences between drug bound and apo samples were calculated from the measured ^13^C and ^15^N chemical shifts according to:

(1) $$\Delta \delta =\sqrt{\left[\sum_{C_{i}}{\left(\delta_{C_{i}}^{\mathrm{drug}}-\delta_{C_{i}}^{apo}\right)}^{2}+\frac{{\left(\delta_{N}^{\mathrm{drug}}-\delta_{N}^{\mathrm{apo}}\right)}^{2}}{2.5}\right]}$$

2D heatmaps of normalized water-edited 2D NCA spectra were generated using an in-house Python script that removes spectral noise while calculating intensity ratios. The intensities of the 9 ms and 100 ms spin diffusion spectra of the ERGIC-bound ETM were read using the NMRglue package ^47^. Spectral intensity was noise filtered by setting signal lower than 3.5 times the average noise level in an empty region of the 2D spectrum to zero for the S spectrum and to a large number for the S_0_ spectrum ^28,48^. The intensities were divided and scaled by the number of scans to obtain a 2D contour map, I_9 ms_/I_100 ms_.

**Figure 2.**
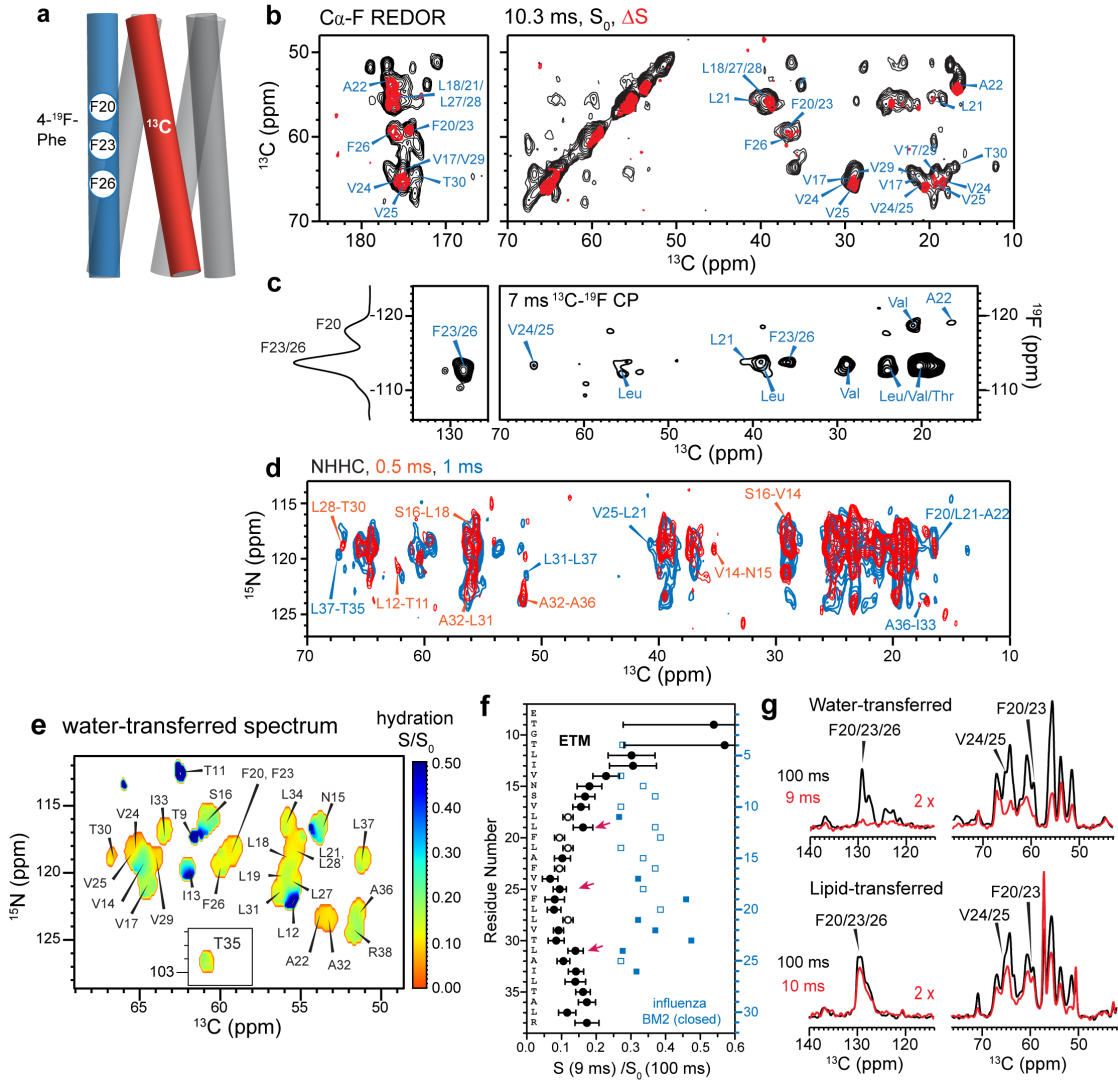
Determination of interhelical distances and water accessibility of membrane-bound ETM. (**a**) Schematic of mixed 4F-Phe and ^13^C-labeled ETM in a five-helix bundle. (**b**) 2D ^13^Cα-F REDOR spectra of ERGIC-membrane bound ETM. The REDOR mixing time was 10.3 ms. The difference spectrum (*orange*) shows residues that are close to the fluorines. (**c**) 2D ^13^C-^19^F correlation spectrum allows assignment of the −118 ppm peak to F20 due to a cross peak with A22, while the −113 ppm peak is assigned to F23/F26 based on correlations with F23, F26, and V24/V25. A 1D ^1^H-^19^F CP spectrum is overlaid on the left. (**d**) 2D NHHC correlation spectrum of mixed ^13^C and ^15^N labeled ETM, measured using 0.5 ms (*red*) and 1 ms (*black*) ^1^H mixing. All peaks arise from interhelical contacts. Selected assignments are given. **(e)** Residue-specific water accessibilities of ERGIC-bound ETM, obtained from the intensity ratios of water-edited spectra measured with 9 ms and 100 ms ^1^H mixing. Higher values (*blue*) indicate higher water accessibility. (**f**) Residue-specific N-Cα cross peak intensity ratios in the 9 ms and 100 ms water-edited spectra of ETM (black). Closed and open circles indicate resolved and overlapped peaks, respectively. For comparison, the water-edited intensities for the high-pH closed state of the influenza BM2 channel (blue squares) are much higher, indicating that the ETM pore is drier than the BM2 pore. (**g**) Water-edited and lipid-edited 1D ^13^C spectra of ERGIC-membrane bound ETM. The Phe signals are high in the lipid-edited spectra but very low in the water-edited spectra, indicating that the three Phe residues are poorly hydrated and point to the lipids or the helix-helix interface.

The water accessibility data for the closed high-pH state of influenza BM2 proton channel (Fig. 2f) for comparison with the ETM data were originally measured in 2D ^13^C-^13^C correlation spectra with 4 ms (S) and 100 ms (S_0_) ^1^H-^1^H spin diffusion ^28^. To enable comparison with the ETM watere-dited spectra measured at 9 ms and 100 ms ^1^H mixing, we scaled the BM2 S (4 ms) /S_0_ (100 ms) ratios by the integrated aliphatic intensity ratio of 1.976 between the 1D BM2 water-edited spectra with 9 ms and 4 ms ^1^H mixing. This scaling factor was verified to be accurate for two resolved sites, T24 and G26, in the 1D ^13^C spectra of BM2.

### Simulation of the ^13^C-^19^F REDOR curves

^13^C-^19^F REDOR data were simulated using the SIMPSON software ^49^. The simulations accounted for finite ^19^F and ^13^C 180° pulse lengths and ^19^F pulse imperfections by co-adding REDOR curves for ^19^F flip angles of 180° to 145° using a normal distribution centered at 180° with a standard deviation of 15° ^43^. The simulations also included ^19^F chemical shift anisotropy (CSA), which was obtained from the ^19^F CSA sideband patterns measured at 293 K under 14 kHz MAS. The sideband intensities were fit using the Solids Lineshape Analysis module in Topspin. The best-fit CSA was δ_CSA_ = 55±2 ppm and η = 0.6±0.1 for the ^19^F peak at δ_iso_ = −113.5 ppm and δ_CSA_ = 53±2 ppm and η = 0.5±0.1 for the ^19^F peak at δ_iso_ = −117.5 ppm. These CSAs indicate that all three 4-^19^F-Phe residues are immobilized.

REDOR distance analysis required two other considerations. First, the 1 : 1 ^13^C and ^19^F mixed peptides means that only 50% of all ^13^C-labeled helices have an adjacent ^19^F-labeled helix. Thus, the lowest possible REDOR S/S0 value is 0.5. Second, while most ^13^C-^19^F REDOR restraints came from 2D ^13^C-^13^C resolved peaks, dephasing to sidechain carbons were obtained from 1D ^13^C spectra with resonance overlap. These overlapped peaks will not experience complete dipolar dephasing if some of the carbons contributing to an overlapped signal are far from a fluorine. We first identified the residues experiencing dephasing by ^19^F from the 2D ^13^C-^13^C correlation spectra. These peaks then guided the assignment of the 1D ^13^C-^19^F spectra. For example, both A22 and A32 Cβ resonate at 16.6 ppm, but only A22 Cα is dephased in the 2D ^13^C-^13^C spectrum (Fig. 2b). Thus, we assigned the 16.6 ppm dephased signal in the 1D ^13^C-^19^F REDOR spectra to A22 Cβ. Making the reasonable assumption that each Ala Cβ contributes equal intensity, we account for this overlap factor by correcting the experimental dephasing (S/S_0_)_*exp*_ values according to:

(2) $${\left(S/S_{0}\right)}_{adj}=1-\left[2\times \left(1-{\left(S/S_{0}\right)}_{\exp}\right)\times f\right]$$

where *f* is the fraction of an overlapped ^13^C peak that is dephased by ^19^F. For example, for the 2-fold overlapped 16.6-ppm Ala Cβ peak with *f* = 2, the lowest possible (S/S_0_)_*exp*_ value is ~0.75, which gives a minimal (S/S_0_)_*adj*_ of ~0.0.

The random uncertainty σ(S/S_0_)_*exp*_ of the measured (S/S_0_)_*exp*_ values were propagated from the signal-to-noise ratios (SNRs) of the REDOR S_0_ and S spectra. The upper and lower limits for the (S/S_0_)_*adj*_ values were obtained by adding or subtracting the σ(S/S_0_)_*exp*_ to the (S/S_0_)_*exp*_ values before using equation (2), respectively. Best-fit distances were obtained as the distance with the lowest χ^2^ value between the (S/S_0_)_*adj*_ values and simulated S/S_0_ intensities. Upper and lower distance limits were specified using the upper and lower limits for the (S/S_0_)_*adj*_ values calculated as described above. For an upper limit of (S/S_0_)_*adj*_ >0.95 indicating a negative contact (i.e. dephasing was not significant), an upper limit of 50 Å was used. The final lower and upper distance limits for structure calculation were set by multiplying the uncertainty obtained in this manner by 2 times or by choosing distances that are 2.0 Å from the best-fit value, whichever was larger, to loosen the constraints.

### XPLOR-NIH structure calculations and analysis

Initial structure calculation attempts using ambiguous interhelical contacts, where a central helix can contact both neighboring helices, did not converge. Thus, we generated parallel pentameric models (Fig. S7) to specify the ^13^C-^19^F and NHHC intermolecular distance restraints in a directional fashion where possible. The models take into account the water- and lipid-edited spectra (*vide infra*) to pinpoint the pore-facing versus lipid-facing orientation of the residues. An ideal helix model that puts N15, L19, V25, L31 and T35 to be pore-facing and Phe sidechains to be lipid-facing does not satisfy all the experimental constraints (Fig. S7a). The measured Cβ secondary shifts (Fig. S3b), with L21 having a 1.4 ppm downfield-shifted Cβ compared to the average of all other helical Leu residues, indicate that the helix is disordered between residues F20 and F23, consistent with previous solution NMR data ^10^. Given this disorder, we generated four alternative models (Fig. S7b–e) that satisfy the measured interhelical Phe-Phe ^13^C-^19^F contacts. Only one model with F26-F23 interhelical contact adequately reproduces the key features of the experimental data. This model was then used to disambiguate the NHHC and ^13^C-^19^F distance restraints (Tables S2, S3), by mainly considering only residues that are less than four residues away in the primary sequence and that are in close proximity between two helical wheels. With this approach, 42 of the 87 interhelical restraints were set to be unambiguous.

We calculated the ETM structure for residues 8–38 using XPLOR-NIH ^50^ hosted on the NMRbox computing platform ^51^. The calculation contained two stages. In the first, annealing, stage, five extended ETM monomers were placed in a parallel pentamer geometry with each monomer located 20 Å from the center of the pentamer. A total of 120 independent XPLOR-NIH runs were performed with 5,000 steps of torsion angle dynamics at 5,000 K followed by annealing to 20 K in decrements of 20 K with 100 steps at each temperature. After the annealing, final energy minimizations in torsion angle and Cartesian coordinates were carried out. The five monomers were restrained to be identical in the annealing step using the non-crystallographic symmetry term PosDiffPot and the translational symmetry term DistSymmPot. Chemical-shift derived (ϕ, ψ) torsion angles predicted by TALOS-N were implemented with the XPLOR dihedral angle restraint term CDIH with ranges set to the higher value between twice the TALOS-N predicted uncertainty and 20°. The interhelical distance restraints (Tables S2, S3) were implemented using the NOE potential. Distance upper limits were set to 9.0 Å and 11.5 Å for 500 μs and 1000 μs of ^1^H-^1^H mixing for the NHHC constraints. Negative REDOR contacts, i.e., ^13^C sites without dephasing, were implemented as two NOE’s: one to each neighboring helix. Implicit hydrogen bonds using the hydrogen-bonding database potential term HBDB were implemented during annealing to favor formation of the α-helix conformation. Finally, standard XPLOR potentials were used to restrain the torsion angles using a structural database with the term TorsionDB, and standard bond angles and lengths were set with terms BOND, ANGL, IMPR and RepelPot. The structures were sorted by energy, using all the potentials in the calculation. The scales for all potentials are given in Table S4.

In the second, structure refinement, stage, the three lowest-energy structures from the annealing stage were used as independent inputs. A total of 64 independent XPLOR-NIH runs from each of the three starting structures were performed with 5,000 steps of torsion angle dynamics at 1,000 K followed by annealing to 20 K in decrements of 10 K with 100 steps at each temperature. This was followed by final energy minimizations in torsion angle and Cartesian coordinates. All the potentials employed in annealing were also used during refinement, with two additions. The ^13^C-^13^C correlations were implemented as intramolecular NOE distance restraints with an upper limit of 8.0 Å. Inter-residue cross peaks to long hydrophobic side chains such as Phe, Ile, and Leu were sometimes violated, and consequently the upper limits for these 5% of restraints were increased to 12.0 Å. Explicit hydrogen bonds for residues I13 (hydrogen-bonded to V17) – N15 (hydrogen-bonded to L19) and F23 (hydrogen-bonded to L27) – T30 (hydrogen-bonded to L34) were substituted for implicit hydrogen bonds, using the same HBDB potential. Finally, the scales of the NOE, Repel, and TorsionDB potentials were increased (Table S4). All 192 structures from the three independent runs were pooled and sorted using the CDIH, NOE, HBDB, BOND, ANGL, IMPR, Repel and Repel14 potentials, while excluding PosDiffPot, DistSymmPot and TorsionDB potentials. The ten structures with the lowest energies across the specified potentials were included in the final structural ensemble.

Graphical images depicting the structures were generated in PyMOL v2.3.4. The reported channel radii were calculated using the HOLE program ^52^, and represent the radii of the largest sphere that can be accommodated from exclusion of the van der Waals diameter of all atoms at each XY plane along the Z channel coordinate, which is collinear with the bilayer normal and the putative direction of ion permeation. The cutoff radius for the calculation was set to be 5 Å. The output from HOLE was visualized in PyMOL by setting the van der Waals radius of the HOLE-generated spheres ‘SPH’ to the b-factor values of the SPH output by HOLE.

### Docking of HMA to ETM structure

**Figure 4.**
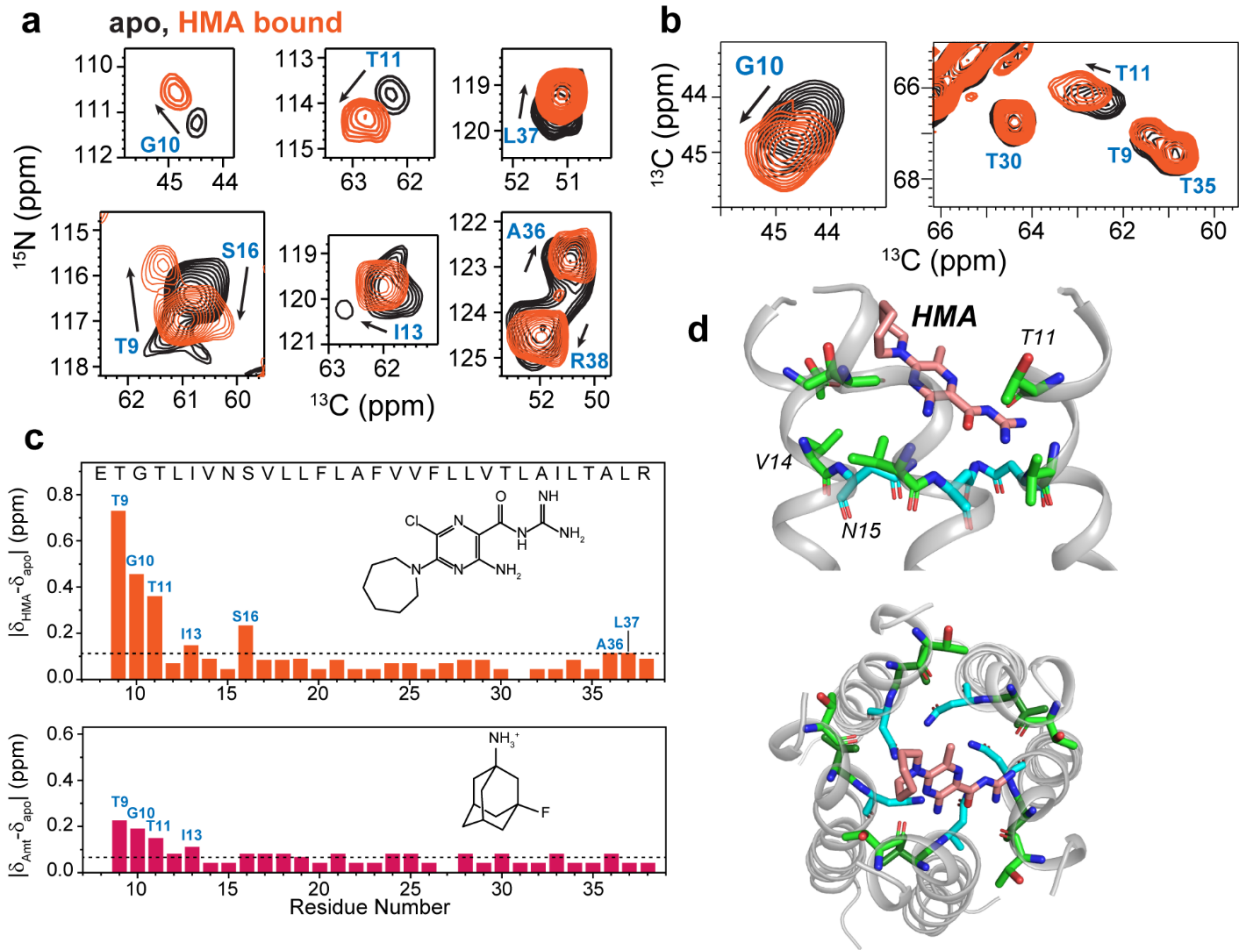
Effects of drug binding to ETM in DMPC : DMPG membranes. (**a**) 2D ^15^N-^13^Cα correlation spectra of the apo (*black*) and HMA-bound ETM (*orange*), showing chemical shift perturbations by HMA. (**c**) 2D ^13^C-^13^C correlation spectra, showing similar CSPs by HMA. (**c**) Per-residue chemical shift perturbations induced by HMA and AMT. N-terminal residues are the most perturbed by both drugs, and HMA causes larger perturbation than AMT. Dashed lines indicate the average CSPs. (**d**) A representative docking pose of HMA. The drug binds to the N-terminal vestibule, with the guanidinium group interacting with T11.

The coordinate file for HMA was generated from bond connectivity using the Chem3D module of ChemDraw Professional 18.1. Ligand geometry was optimized within Chem3D using the MM2 energy minimization module. Docking was performed using the HADDOCK 2.4 webserver using the ensemble of the ten lowest-energy protein structures calculated in XPLOR-NIH. The docking was constrained only with an active list. Active residues were defined as the N-terminal residues with significant CSPs (Fig. 4c):T9, G10, T11, I13, and S16. Several docking runs were conducted using constraints to this list of residues on all helices, one of the five helices, and different combinations of two of the five helices. Docking calculations were performed using default settings in the HADDOCK 2.4 webserver interface, except that the solvent for the final structural refinement were varied as DMSO and water. The N- and C-termini were set as uncharged as the structural model does not include the full protein sequence. Passive residues were automatically defined around the active residues with a 6.5 Å surface radius cutoff. Non-polar hydrogens were removed from the calculation, and 2 partitions for random exclusion of Ambiguous Interaction Restraints (‘AIRs’) were used (50% of AIRs were randomly excluded in the calculation). The docking used 1000 structures for rigid-body docking with 5 trials of rigid-body minimization. Semi-flexible refinement was done with 200 structures selected from the rigid-body minimization stage. Final refinement with explicit solvent (DMSO or water) were performed on all 200 structures from semi-flexible refinement. Output structures were aligned and analyzed in Pymol 2.3.5.

In docking with DMSO as a refinement solvent, 200 refined structures were grouped into 5 clusters, with 154 structures belonging to the lowest energy cluster 1. The four best structures of this cluster had an average HADDOCK score of −29.8 +/− 2.1, and a Z-score of −1.7. Similar results were obtained with docking with water as a refinement solvent, the 200 refined structures were grouped into three clusters, where cluster 1 contained 170 structures. The four best structures this cluster had a HADDOCK score of −29.3 +/− 1.9, with a Z-score of −1.4.

The majority of docked structures converged to a state where HMA partitioned to the N-terminal entry cavity of the channel, but the HMA orientation is variable. Visual inspection showed three distinct orientations: 1) HMA tilted and diving into the pore with the hexamethylene ring facing up (Fig. S9a), 2) HMA tilted and diving into the pore with the ring facing down (Fig. S9b), 3) HMA laying horizontally across the top of the channel, with the guanidinium intercalated between two helices (Fig S9c). Among the 32 lowest energy structures in the DMSO and water docking results, 13/32 structures belonged to the first mode (ring up), 6/32 to the second mode (ring down), and 13/32 to the third mode (horizontal). All these modes indicate a pore-occlusion mechanism, similar to the amantadine inhibition mechanism of the influenza AM2 proton channel ^9^.

Given the hydrophobic nature of HMA, another possible binding mode would be drug binding from the lipid side to the exterior of the helical bundle. This mode could explain the chemical shift perturbation of the lipid-facing S16, but the mechanism of inhibition would be indirect allosteric narrowing of the pore, and would require multiple drug molecules to bind each pentamer to preserve the symmetry, as only a single set of peaks are observed in the drug-bound protein spectra. Thus we consider this mechanism less likely than direct occlusion of the pore. The lipid-binding mode was only observed in docking runs with only one or two helices containing the active residues.

## Supplementary Material

Supplement

1

## Figures and Tables

**Figure 1.**
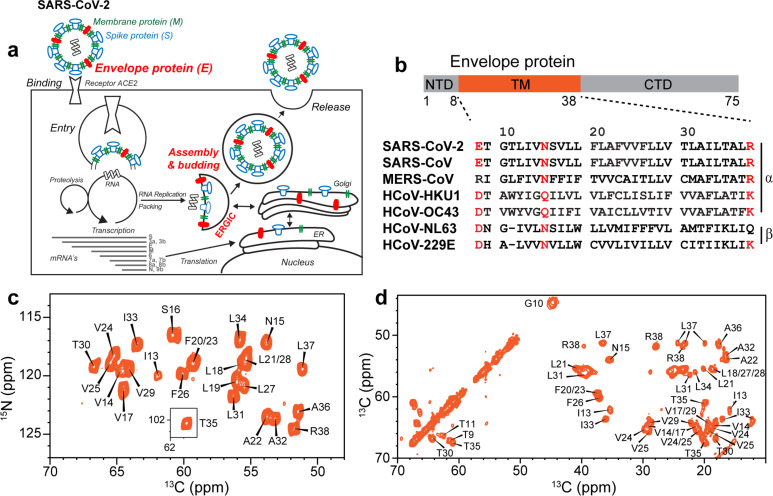
Function, sequence, and fingerprint NMR spectra of the SARS-CoV-2 envelope protein. (**a**) E mediates the budding and release of SARS-CoV-2 from the host cell ERGIC lumen and forms a cation-selective channel. (**b**) Sequence domains of E and sequence alignment of the transmembrane segment of E of SARS-CoV-2 with other human-infecting coronaviruses. Key conserved polar residues are shown in red. (**c**) 2D ^15^N-^13^Cα correlation spectrum and (**d**) 2D ^13^C-^13^C spectrum of ERGIC-membrane bound ETM. The spectra, measured at ambient temperature, show predominantly α-helical chemical shifts, and have high sensitivity and resolution, indicating that the ETM helical bundle is rigid and ordered in the ERGIC-mimetic lipid bilayer.

**Figure 3.**
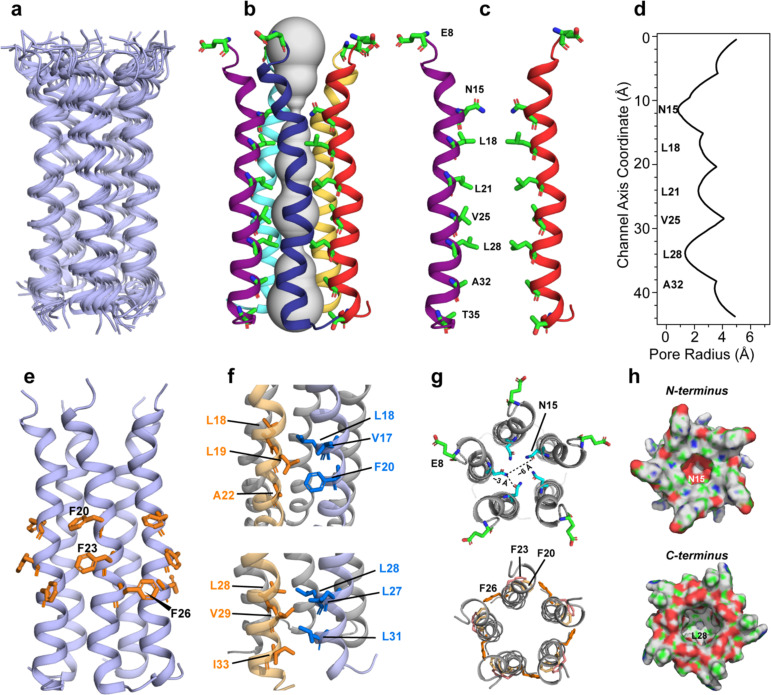
Structure of the SARS-CoV-2 envelope protein TM domain in ERGIC-mimetic lipid bilayers. **(a)** Ensemble of the ten lowest-energy structures. (**b**) Sideview of the lowest-energy structure along with pore water (gray), depicted using the HOLE program. Pore-lining residues are shown in sticks. (**c**) Simplified view showing two helices (*i* and *i*+2) with the pore-facing residues. (**d**) Pore radius of ETM obtained from the HOLE program. (**e**) Sideview of the pentamer, displaying the Phe triplet in the middle of the TM segment. (**f**) Two clusters of methyl-interdigitating Leu, Ile and Val residues, stabilizing the helix-helix interface. (**g**) Top views of the disordered N-terminal E8, the pore-occluding N15, and the three Phe residues bridging the helix-helix interface. (**h**) Surface plots of the pentamer, showing that the N-terminal vestibule is slightly tighter than the C-terminal opening.

**Table 1.** Structure calculation and refinement statistics for ERGIC-membrane bound ETM.

**NMR distance and dihedral constraints**	
Distance constraints	
Total NOE	283 × 5
Inter-residue	283 × 4
Sequential (|*i* – *j*| = 1)	125 × 5
Medium range (2 ≤ |*i* – *j*| ≤ 4)	71 × 5
Long range (|*i* – *j*| ≥ 5)	0 × 5
Intermolecular	87 × 5
Total dihedral-angle restraints	
ϕ	28 × 5
ψ	28 × 5
Total orientation constraints	0 × 5
^1^H-^15^N dipolar couplings	0 × 5
Hydrogen bond restraints	11 × 5

**Structure statistics**	
Violations (mean ± s.d.)	
Distance constraints (Å)	0.18 ± 0.09
Dihedral-angle constraints (°)	0.62 ± 0.22
Max. dihedral-angle violation (°)	6.52
Max. distance-constraint violation (Å)	1.64
Deviations from idealized geometry	
Bond lengths (Å)	0.004 ± 0.001
Bond angles (°)	0.47 ± 0.06
Impropers (°)	0.34 ± 0.04
Average pairwise r.m.s. deviation (Å)	
Heavy atom (residues 10–36)	2.43 ± 0.75
Backbone (residues 10–36)	2.08 ± 0.83

## References

[1] Weiss S. R. & Navas-Martin S. Coronavirus pathogenesis and the emerging pathogen severe acute respiratory syndrome coronavirus. Microbiol. Mol. Biol. Rev. 69, 635–664, (2005). 16339739 10.1128/MMBR.69.4.635-664.2005 PMC1306801

[2] Wilson L., McKinlay C., Gage P. & Ewart G. SARS coronavirus E protein forms cation-selective ion channels. Virology 330, 322–331, (2004). 15527857 10.1016/j.virol.2004.09.033 PMC7111769

[3] Parthasarathy K. Expression and purification of coronavirus envelope proteins using a modified β-barrel construct. Protein Expr. Purif. 85, 133–141, (2012). 22819936 10.1016/j.pep.2012.07.005 PMC7129850

[4] Schoeman D. & Fielding B. C. Coronavirus envelope protein: current knowledge. Virol. J. 16, 69, (2019). 31133031 10.1186/s12985-019-1182-0 PMC6537279

[5] Nieto-Torres J. L. Severe acute respiratory syndrome coronavirus E protein transports calcium ions and activates the NLRP3 inflammasome. Virology 485, 330–339, (2015). 26331680 10.1016/j.virol.2015.08.010 PMC4619128

[6] Wilson L., Gage P. & Ewart G. Hexamethylene amiloride blocks E protein ion channels and inhibits coronavirus replication. Virology 353, 294–306, (2006). 16815524 10.1016/j.virol.2006.05.028 PMC7111787

[7] Torres J. Conductance and amantadine binding of a pore formed by a lysine-flanked transmembrane domain of SARS coronavirus envelope protein. Protein Sci. 16, 2065–2071, (2007). 17766393 10.1110/ps.062730007 PMC2206980

[8] Hong M. & DeGrado W. F. Structural basis for proton conduction and inhibition by the influenza M2 protein. Protein Sci. 21, 1620–1633, (2012). 23001990 10.1002/pro.2158 PMC3527700

[9] Cady S. D. Structure of the amantadine binding site of influenza M2 proton channels in lipid bilayers. Nature 463, 689–692, (2010). 20130653 10.1038/nature08722 PMC2818718

[10] Pervushin K. Structure and inhibition of the SARS coronavirus envelope protein ion channel. PLoS Pathog. 5, e1000511, (2009). 19593379 10.1371/journal.ppat.1000511 PMC2702000

[11] DeDiego M. L. A severe acute respiratory syndrome coronavirus that lacks the E gene is attenuated in vitro and in vivo. J. Virol. 81, 1701–1713, (2007). 17108030 10.1128/JVI.01467-06 PMC1797558

[12] Nieto-Torres J. L. Severe acute respiratory syndrome coronavirus envelope protein ion channel activity promotes virus fitness and pathogenesis. PLoS Pathog. 10, e1004077, (2014). 24788150 10.1371/journal.ppat.1004077 PMC4006877

[13] Verdiá-Báguena C. Coronavirus E protein forms ion channels with functionally and structurally-involved membrane lipids. Virology 432, 485–494, (2012). 22832120 10.1016/j.virol.2012.07.005 PMC3438407

[14] Torres J., Wang J., Parthasarathy K. & Liu D. X. The transmembrane oligomers of coronavirus protein E. Biophys. J. 88, 1283–1290, (2005). 15713601 10.1529/biophysj.104.051730 PMC1305130

[15] Li Y., Surya W., Claudine S. & Torres J. Structure of a conserved Golgi complex-targeting signal in coronavirus envelope proteins. J. Biol. Chem. 289, 12535–12549, (2014). 24668816 10.1074/jbc.M114.560094 PMC4007446

[16] Arbely E. A highly unusual palindromic transmembrane helical hairpin formed by SARS coronavirus E protein. J. Mol. Biol. 341, 769–779, (2004). 15288785 10.1016/j.jmb.2004.06.044 PMC7134595

[17] Surya W., Li Y. & Torres J. Structural model of the SARS coronavirus E channel in LMPG micelles. Biochim. Biophys. Acta 1860, 1309–1317, (2018). 10.1016/j.bbamem.2018.02.017 PMC7094280 29474890

[18] Gullion T. & Schaefer J. Rotational echo double resonance NMR. J. Magn. Reson. 81, 196–200, (1989). 10.1016/j.jmr.2011.09.003 22152360

[19] Dregni A. J. In vitro 0N4R tau fibrils contain a monomorphic beta-sheet core enclosed by dynamically heterogeneous fuzzy coat segments. Proc. Natl. Acad. Sci. U.S.A. 116, 16357–16366, (2019). 31358628 10.1073/pnas.1906839116 PMC6697781

[20] Williams J. K. & Hong M. Probing membrane protein structure using water polarization transfer solid-state NMR. J. Magn. Reson. 247, 118–127, (2014). 25228502 10.1016/j.jmr.2014.08.007 PMC4398059

[21] Schwieters C. D., Kuszewski J. J., Tjandra N. & Clore G. M. The Xplor-NIH NMR molecular structure determination package. J. Magn. Reson. 160, 65–73, (2003). 12565051 10.1016/s1090-7807(02)00014-9

[22] Choma C., Gratkowski H., Lear J. D. & DeGrado W. F. Asparagine-mediated self-association of a model transmembrane helix. Nat. Struc. Biol. 7, 161–166, (2000). 10.1038/72440 10655620

[23] Hou X., Pedi L., Diver M. M. & Long S. B. Crystal structure of the calcium release-activated calcium channel Orai. Science 338, 1308–1313, (2012). 23180775 10.1126/science.1228757 PMC3695727

[24] Xu C. Computational design of transmembrane pores. Nature 585, 129–134, (2020). 32848250 10.1038/s41586-020-2646-5 PMC7483984

[25] Lee C. The lysosomal potassium channel TMEM175 adopts a novel tetrameric architecture. Nature 547, 472–475, (2017). 28723891 10.1038/nature23269 PMC5901963

[26] Kane Dickson V., Pedi L. & Long S. B. Structure and insights into the function of a Ca(2+)-activated Cl(−) channel. Nature 516, 213–218, (2014). 25337878 10.1038/nature13913 PMC4454446

[27] Yang T. Structure and selectivity in bestrophin ion channels. Science 346, 355–359, (2014). 25324390 10.1126/science.1259723 PMC4341822

[28] Mandala V. S., Loftis A. R., Shcherbakov A. A., Pentelute B. L. & Hong M. Atomic structures of closed and open influenza B M2 proton channel reveal the conduction mechanism. Nat. Struct. Mol. Biol. 27, 160–167, (2020). 32015551 10.1038/s41594-019-0371-2 PMC7641042

[29] Park S. H. Three-dimensional structure of the channel-forming trans-membrane domain of virus protein “u” (Vpu) from HIV-1. J. Mol. Biol. 333, 409–424, (2003). 14529626 10.1016/j.jmb.2003.08.048

[30] Lu J. X., Sharpe S., Ghirlando R., Yau W. M. & Tycko R. Oligomerization state and supramolecular structure of the HIV-1 Vpu protein transmembrane segment in phospholipid bilayers. Protein Sci., (2010). 10.1002/pro.474 PMC2998723 20669237

[31] Cady S. D., Goodman C., Tatko C. D., DeGrado W. F. & Hong M. Determining the orientation of uniaxially rotating membrane proteins using unoriented samples: a 2H, 13C, AND 15N solid-state NMR investigation of the dynamics and orientation of a transmembrane helical bundle. J. Am. Chem. Soc. 129, 5719–5729, (2007). 17417850 10.1021/ja070305e

[32] Nieto-Torres J. L. Subcellular location and topology of severe acute respiratory syndrome coronavirus envelope protein. Virology 415, 69–82, (2011). 21524776 10.1016/j.virol.2011.03.029 PMC4726981

[33] Duart G. SARS-CoV-2 envelope protein topology in eukaryotic membranes. Open Biol. 10, 200209, (2020). 32898469 10.1098/rsob.200209 PMC7536074

[34] Abramson A. An ingestible self-orienting system for oral delivery of macromolecules. Science 363, 611–615, (2019). 30733413 10.1126/science.aau2277 PMC6430586

