# Supplementary material for: Structure and Drug Binding of the SARS-CoV-2 Envelope Protein in Phospholipid Bilayers
Source: Res Sq. 2020 Sep 24:rs.3.rs-77124. Preprint. [Version 1] doi: 10.21203/rs.3.rs-77124/v1 (PMC7523133; doi:10.21203/rs.3.rs-77124/v1)
Supplement: Supplement [file D1000251802valreportfullannotateP1.pdf]

Full wwPDB NMR Structure Validation Report (i)

Sep 14, 2020 – 10:59 AM EDT

PDB ID	:	7K3G
Title	:	SARS-CoV-2 Envelope Protein Transmembrane Domain: Pentameric Structure Determined by Solid-State NMR
Deposited on	:	2020-09-11

This is a Full wwPDB NMR Structure Validation Report.

This report is produced by the wwPDB biocuration pipeline after annotation of the structure.

We welcome your comments at *validation@mail.wwpdb.org*
A user guide is available at
https://www.wwpdb.org/validation/2017/NMRValidationReportHelp
with specific help available everywhere you see the (i) symbol.

CONFIDENTIAL VALIDATION REPORT

The following versions of software and data (see references (i)) were used in the production of this report:

Cyrange	:	Kirchner and Güntert (2011)
NmrClust	:	Kelley et al. (1996)
MolProbity	:	4.02b-467
Percentile statistics	:	20191225.v01 (using entries in the PDB archive December 25th 2019)
RCI	:	v_1n_11_5_13_A (Berjanski et al., 2005)
PANAV	:	Wang et al. (2010)
ShiftChecker	:	2.14.2
Ideal geometry (proteins)	:	Engh & Huber (2001)
Ideal geometry (DNA, RNA)	:	Parkinson et al. (1996)
Validation Pipeline (wwPDB-VP)	:	2.14.2

1 Overall quality at a glance (i)

The following experimental techniques were used to determine the structure:
SOLID-STATE NMR

The overall completeness of chemical shifts assignment is 9%.

Percentile scores (ranging between 0-100) for global validation metrics of the entry are shown in the following graphic. The table shows the number of entries on which the scores are based.

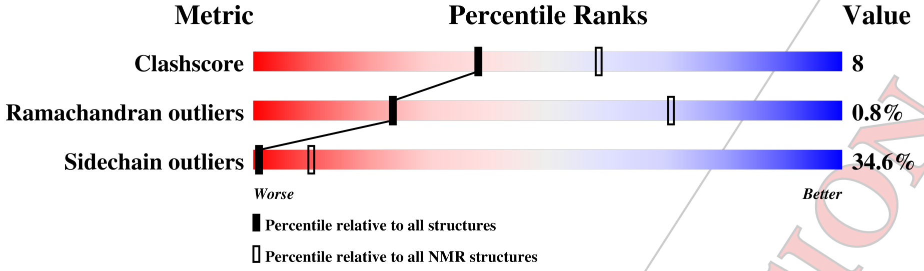

Metric	Whole archive (#Entries)	NMR archive (#Entries)
Clashscore	158937	12864
Ramachandran outliers	154571	11451
Sidechain outliers	154315	11428

The table below summarises the geometric issues observed across the polymeric chains and their fit to the experimental data. The red, orange, yellow and green segments indicate the fraction of residues that contain outliers for >=3, 2, 1 and 0 types of geometric quality criteria. A cyan segment indicates the fraction of residues that are not part of the well-defined cores, and a grey segment represents the fraction of residues that are not modelled. The numeric value for each fraction is indicated below the corresponding segment, with a dot representing fractions <=5%

Mol	Chain	Length	Quality of chain
1	A	31	32% 48% 19%
1	B	31	32% 48% 19%
1	C	31	32% 48% 19%
1	D	31	32% 48% 19%
1	E	31	29% 52% 19%

CONFIDENTIAL VALIDATION REPORT

2 Ensemble composition and analysis (i)

This entry contains 10 models. Model 3 is the overall representative, medoid model (most similar to other models). The authors have identified model 1 as representative, based on the following criterion: *lowest energy*.

The following residues are included in the computation of the global validation metrics.

Well-defined (core) protein residues			
**Well-defined core**	**Residue range (total)**	**Backbone RMSD (Å)**	**Medoid model**
1	A:13-A:37, B:13-B:37, C:13-C:37, D:13-D:37, E:13-E:37 (125)	0.83	3

Ill-defined regions of proteins are excluded from the global statistics.

Ligands and non-protein polymers are included in the analysis.

The models can be grouped into 2 clusters. No single-model clusters were found.

Cluster number	Models
1	1, 2, 3, 4, 5, 6, 7, 10
2	8, 9

CONFIDENTIAL VALIDATION REPORT

3 Entry composition (i)

There is only 1 type of molecule in this entry. The entry contains 2545 atoms, of which 1355 are hydrogens and 0 are deuteriums.

- Molecule 1 is a protein called Envelope small membrane protein.

Mol	Chain	Residues	Atoms					Trace
			Total	C	H	N	O	
1	A	31	509	163	271	35	40	0
1	B	31	509	163	271	35	40	0
1	C	31	509	163	271	35	40	0
1	D	31	509	163	271	35	40	0
1	E	31	509	163	271	35	40	0

CONFIDENTIAL VALIDATION REPORT

4 Residue-property plots (i)

4.1 Average score per residue in the NMR ensemble

These plots are provided for all protein, RNA, DNA and oligosaccharide chains in the entry. The first graphic is the same as shown in the summary in section 1 of this report. The second graphic shows the sequence where residues are colour-coded according to the number of geometric quality criteria for which they contain at least one outlier: green = 0, yellow = 1, orange = 2 and red = 3 or more. Stretches of 2 or more consecutive residues without any outliers are shown as green connectors. Residues which are classified as ill-defined in the NMR ensemble, are shown in cyan with an underline colour-coded according to the previous scheme. Residues which were present in the experimental sample, but not modelled in the final structure are shown in grey.

- Molecule 1: Envelope small membrane protein

Chain A:

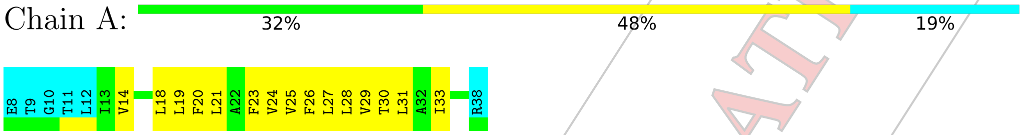

- Molecule 1: Envelope small membrane protein

Chain B:

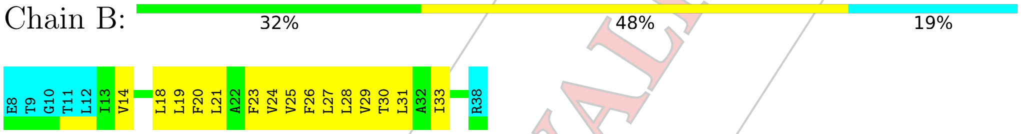

- Molecule 1: Envelope small membrane protein

Chain C:

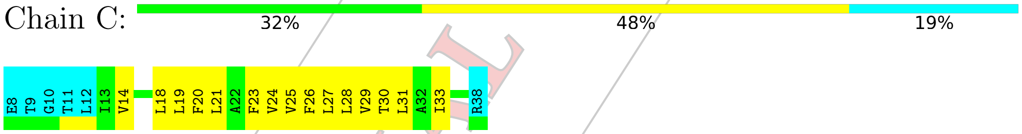

- Molecule 1: Envelope small membrane protein

Chain D:

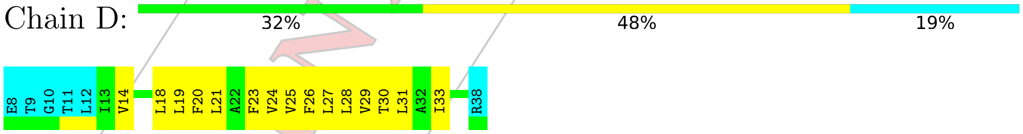

- Molecule 1: Envelope small membrane protein

Chain E:

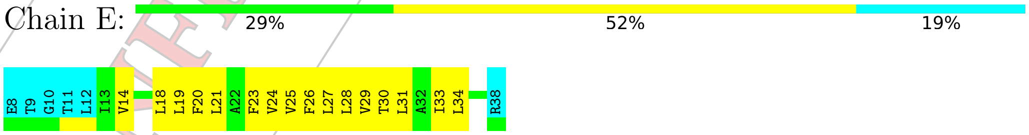

CONFIDENTIAL VALIDATION REPORT

4.2 Scores per residue for each member of the ensemble

Colouring as in section 4.1 above.

4.2.1 Score per residue for model 1

• Molecule 1: Envelope small membrane protein

Chain A:

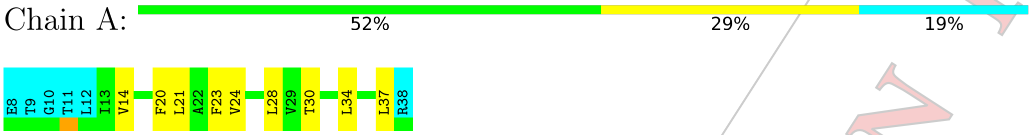

• Molecule 1: Envelope small membrane protein

Chain B:

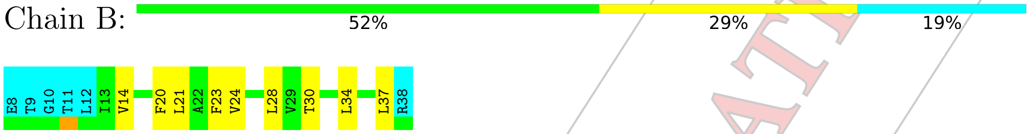

• Molecule 1: Envelope small membrane protein

Chain C:

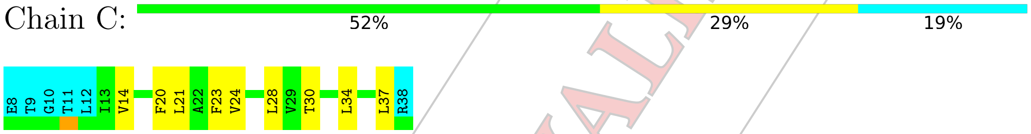

• Molecule 1: Envelope small membrane protein

Chain D:

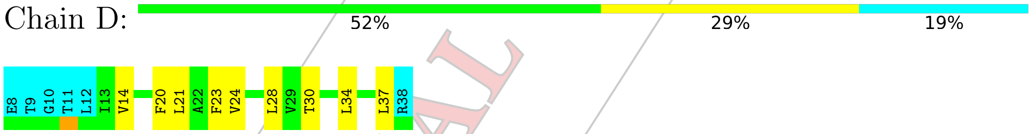

• Molecule 1: Envelope small membrane protein

Chain E:

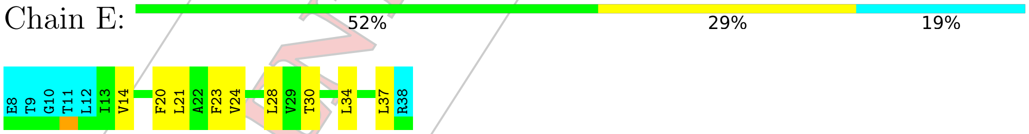

4.2.2 Score per residue for model 2

• Molecule 1: Envelope small membrane protein

Chain A:

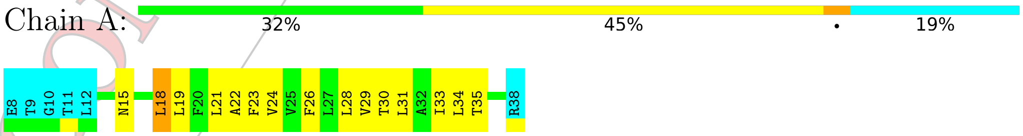

CONFIDENTIAL VALIDATION REPORT

- Molecule 1: Envelope small membrane protein

Chain B:

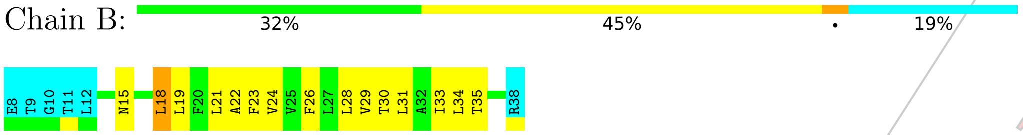

- Molecule 1: Envelope small membrane protein

Chain C:

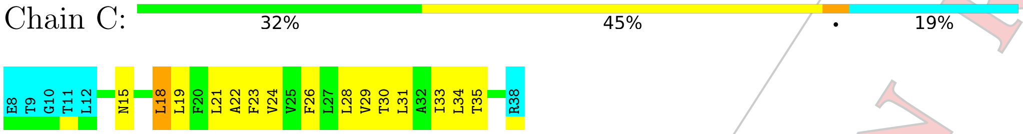

- Molecule 1: Envelope small membrane protein

Chain D:

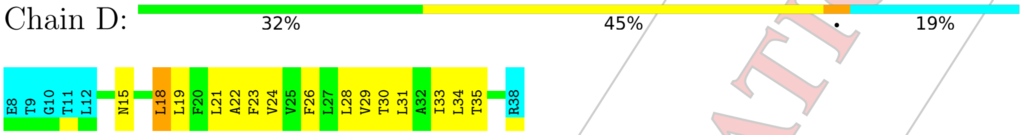

- Molecule 1: Envelope small membrane protein

Chain E:

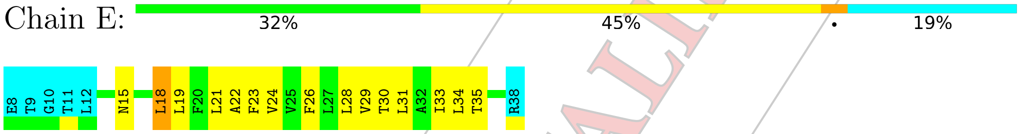

4.2.3 Score per residue for model 3 (medoid)

- Molecule 1: Envelope small membrane protein

Chain A:

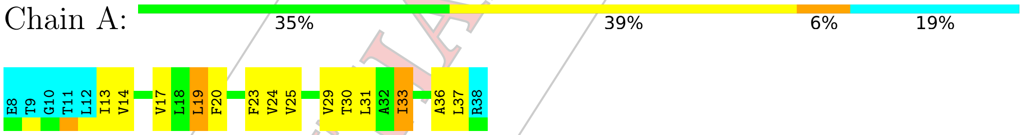

- Molecule 1: Envelope small membrane protein

Chain B:

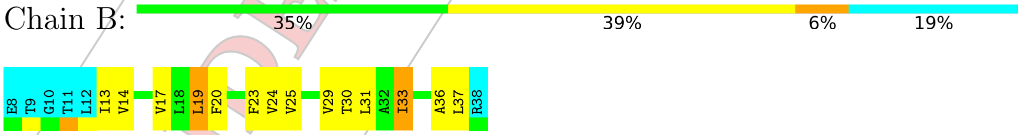

- Molecule 1: Envelope small membrane protein

Chain C:

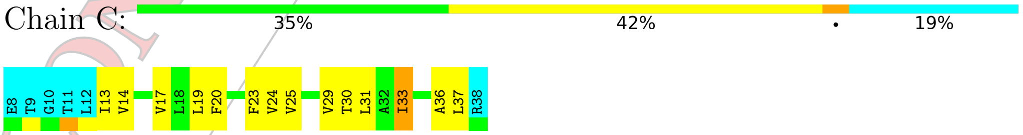

CONFIDENTIAL VALIDATION REPORT

• Molecule 1: Envelope small membrane protein

Chain D:

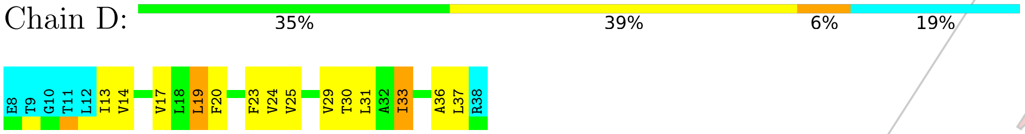

• Molecule 1: Envelope small membrane protein

Chain E:

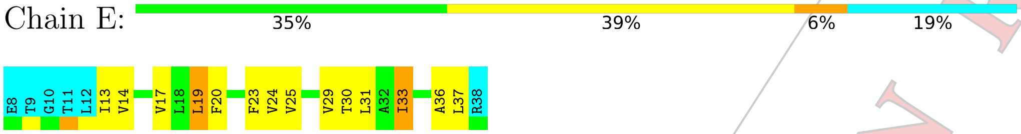

4.2.4 Score per residue for model 4

• Molecule 1: Envelope small membrane protein

Chain A:

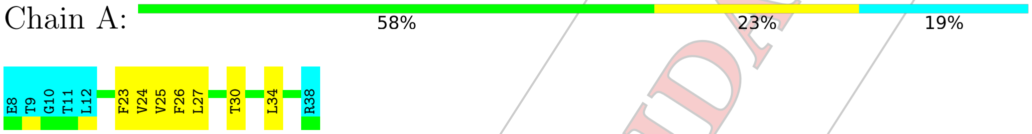

• Molecule 1: Envelope small membrane protein

Chain B:

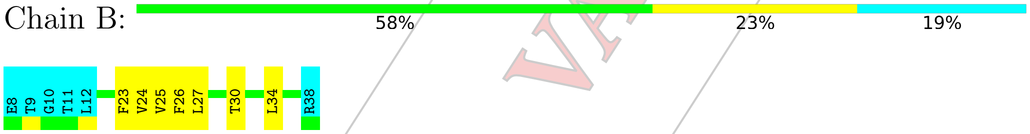

• Molecule 1: Envelope small membrane protein

Chain C:

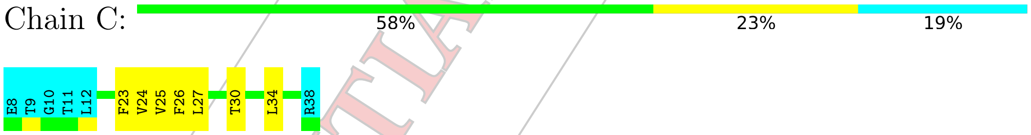

• Molecule 1: Envelope small membrane protein

Chain D:

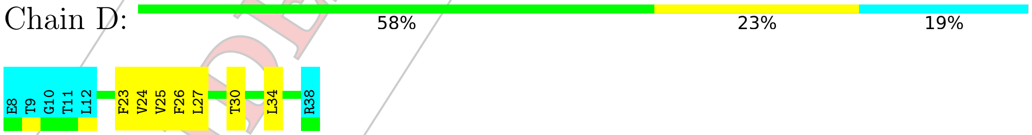

• Molecule 1: Envelope small membrane protein

Chain E:

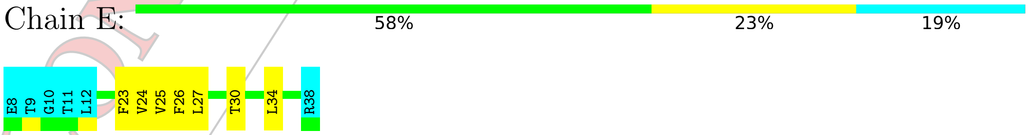

CONFIDENTIAL VALIDATION REPORT

4.2.5 Score per residue for model 5

- Molecule 1: Envelope small membrane protein

Chain A:

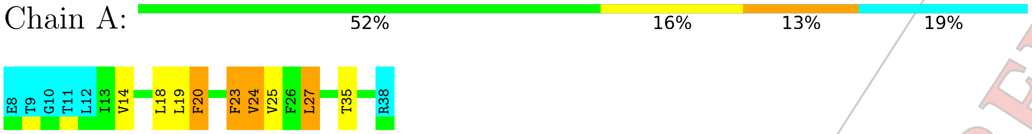

- Molecule 1: Envelope small membrane protein

Chain B:

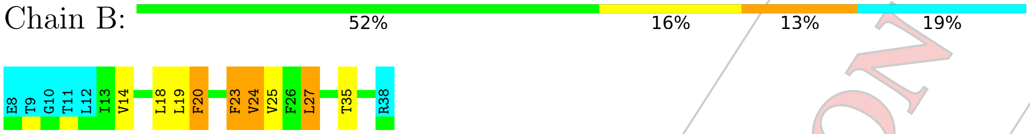

- Molecule 1: Envelope small membrane protein

Chain C:

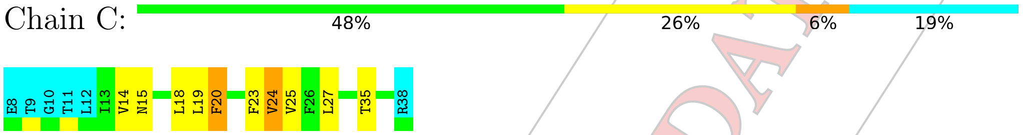

- Molecule 1: Envelope small membrane protein

Chain D:

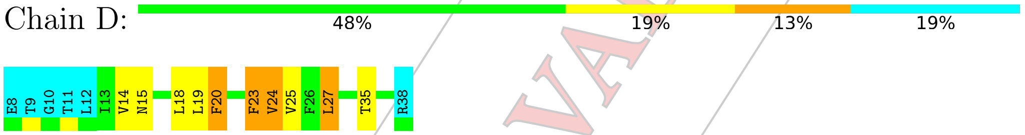

- Molecule 1: Envelope small membrane protein

Chain E:

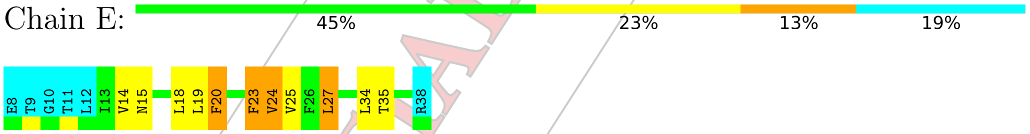

4.2.6 Score per residue for model 6

- Molecule 1: Envelope small membrane protein

Chain A:

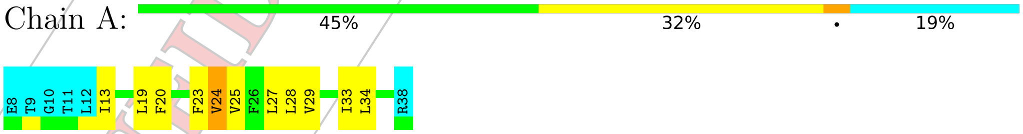

- Molecule 1: Envelope small membrane protein

Chain B:

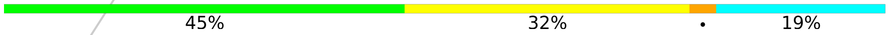

CONFIDENTIAL VALIDATION REPORT

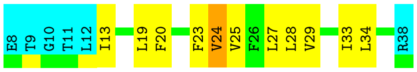

• Molecule 1: Envelope small membrane protein

Chain C: 45% 32% • 19%

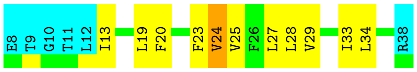

• Molecule 1: Envelope small membrane protein

Chain D: 45% 32% • 19%

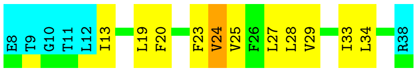

• Molecule 1: Envelope small membrane protein

Chain E: 45% 32% • 19%

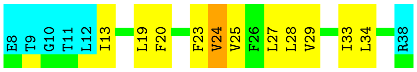

4.2.7 Score per residue for model 7

• Molecule 1: Envelope small membrane protein

Chain A: 45% 29% 6% 19%

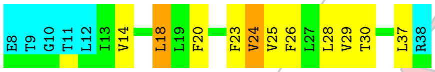

• Molecule 1: Envelope small membrane protein

Chain B: 45% 29% 6% 19%

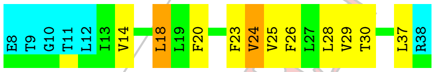

• Molecule 1: Envelope small membrane protein

Chain C: 45% 32% • 19%

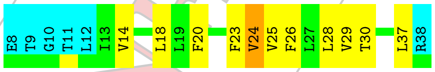

• Molecule 1: Envelope small membrane protein

Chain D: 45% 32% • 19%

CONFIDENTIAL VALIDATION REPORT

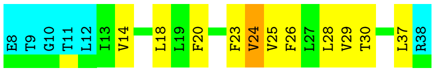

• Molecule 1: Envelope small membrane protein

Chain E:

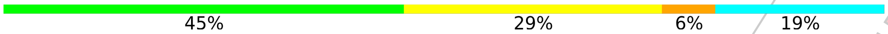

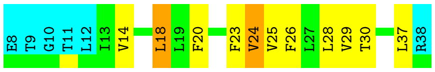

4.2.8 Score per residue for model 8

• Molecule 1: Envelope small membrane protein

Chain A:

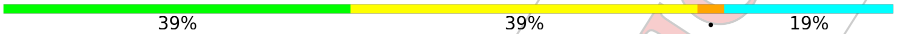

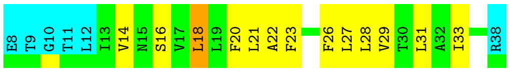

• Molecule 1: Envelope small membrane protein

Chain B:

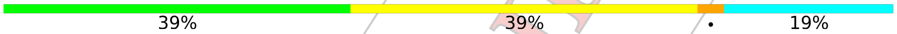

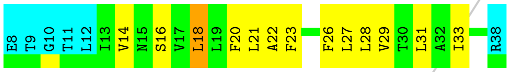

• Molecule 1: Envelope small membrane protein

Chain C:

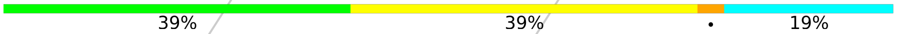

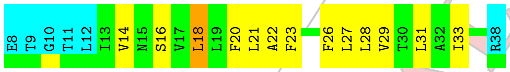

• Molecule 1: Envelope small membrane protein

Chain D:

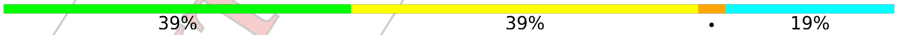

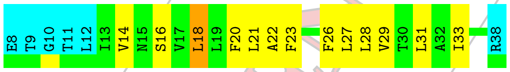

• Molecule 1: Envelope small membrane protein

Chain E:

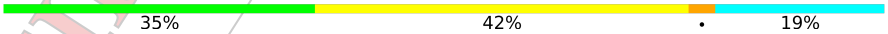

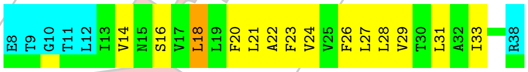

CONFIDENTIAL VALIDATION REPORT

4.2.9 Score per residue for model 9

- Molecule 1: Envelope small membrane protein

Chain A:

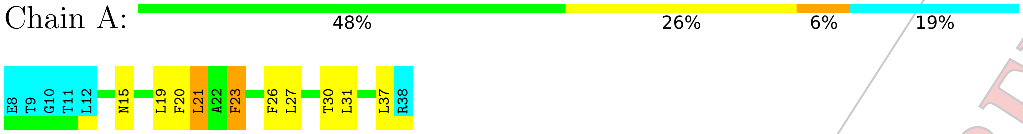

- Molecule 1: Envelope small membrane protein

Chain B:

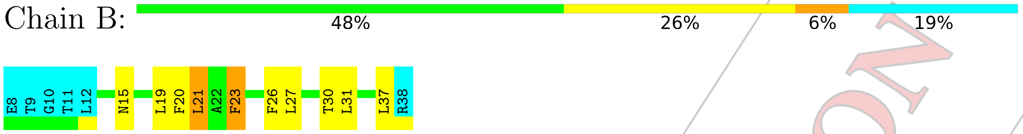

- Molecule 1: Envelope small membrane protein

Chain C:

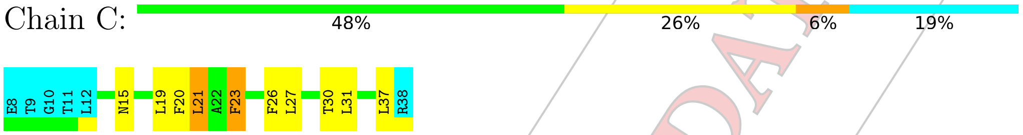

- Molecule 1: Envelope small membrane protein

Chain D:

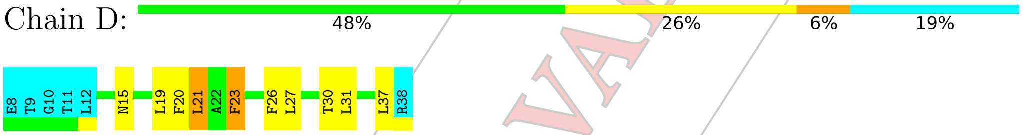

- Molecule 1: Envelope small membrane protein

Chain E:

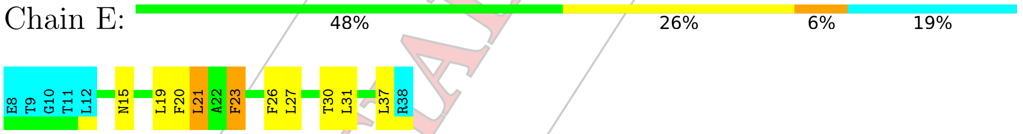

4.2.10 Score per residue for model 10

- Molecule 1: Envelope small membrane protein

Chain A:

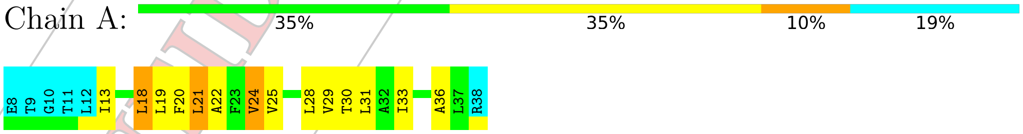

- Molecule 1: Envelope small membrane protein

Chain B:

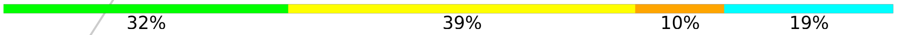

CONFIDENTIAL VALIDATION REPORT

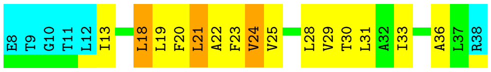

• Molecule 1: Envelope small membrane protein

Chain C:

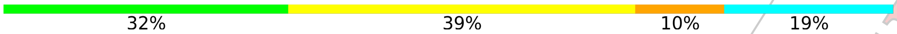

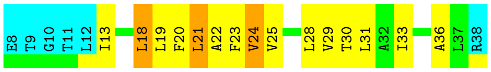

• Molecule 1: Envelope small membrane protein

Chain D:

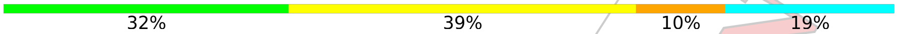

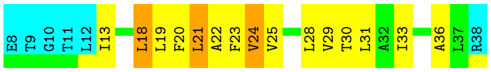

• Molecule 1: Envelope small membrane protein

Chain E:

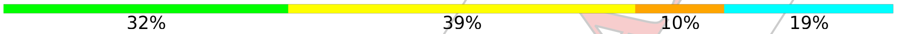

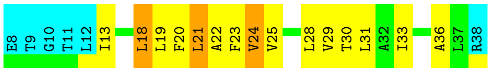

CONFIDENTIAL VALIDATION REPORT

5 Refinement protocol and experimental data overview (i)

The models were refined using the following method: *simulated annealing*.

Of the 192 calculated structures, 10 were deposited, based on the following criterion: *structures with the lowest energy*.

The following table shows the software used for structure solution, optimisation and refinement.

Software name	Classification	Version
X-PLOR NIH	structure calculation	2.47
X-PLOR NIH	refinement	2.47

The following table shows chemical shift validation statistics as aggregates over all chemical shift files. Detailed validation can be found in section 7 of this report.

Chemical shift file(s)	D_1000251802_cs_P1.cif.V1
Number of chemical shift lists	1
Total number of shifts	161
Number of shifts mapped to atoms	161
Number of unparsed shifts	0
Number of shifts with mapping errors	0
Number of shifts with mapping warnings	0
Assignment completeness (well-defined parts)	9%

Note: This is a solid-state NMR structure, where hydrogen atoms are typically not assigned a chemical shift value, which may lead to lower completeness of assignment measure.

No validations of the models with respect to experimental NMR restraints is performed at this time.

CONFIDENTIAL VALIDATION REPORT

6 Model quality (i)

6.1 Standard geometry (i)

There are no covalent bond-length or bond-angle outliers.

There are no bond-length outliers.

There are no bond-angle outliers.

There are no chirality outliers.

There are no planarity outliers.

6.2 Too-close contacts (i)

In the following table, the Non-H and H(model) columns list the number of non-hydrogen atoms and hydrogen atoms in each chain respectively. The H(added) column lists the number of hydrogen atoms added and optimized by MolProbity. The Clashes column lists the number of clashes averaged over the ensemble.

Mol	Chain	Non-H	H(model)	H(added)	Clashes
1	A	191	222	222	5±3
1	B	191	222	222	5±4
1	C	191	222	222	5±4
1	D	191	222	222	5±4
1	E	191	222	222	5±4
All	All	9550	11100	11100	165

The all-atom clashscore is defined as the number of clashes found per 1000 atoms (including hydrogen atoms). The all-atom clashscore for this structure is 8.

All unique clashes are listed below, sorted by their clash magnitude.

Atom-1	Atom-2	Clash(Å)	Distance(Å)	Models	
				Worst	Total
1:D:31:LEU:HD23	1:E:33:ILE:HD13	0.76	1.58	8	1
1:B:31:LEU:HD23	1:C:33:ILE:HD13	0.76	1.57	8	1
1:A:31:LEU:HD23	1:B:33:ILE:HD13	0.75	1.58	8	1
1:C:31:LEU:HD23	1:D:33:ILE:HD13	0.74	1.59	8	1
1:A:33:ILE:HD13	1:E:31:LEU:HD23	0.74	1.59	8	1
1:D:18:LEU:O	1:E:19:LEU:HD13	0.57	2.00	10	1
1:A:18:LEU:O	1:B:19:LEU:HD13	0.56	2.00	10	1
1:A:24:VAL:HG13	1:B:25:VAL:HG11	0.56	1.77	10	2
1:C:18:LEU:O	1:D:19:LEU:HD13	0.56	2.00	10	1
1:C:24:VAL:HG13	1:D:25:VAL:HG11	0.55	1.77	10	2

Continued on next page...

CONFIDENTIAL VALIDATION REPORT

Continued from previous page...

Atom-1	Atom-2	Clash(Å)	Distance(Å)	Models	
				Worst	Total
1:D:24:VAL:HG13	1:E:25:VAL:HG11	0.55	1.78	10	2
1:A:24:VAL:HG22	1:B:25:VAL:HG11	0.55	1.78	5	1
1:B:24:VAL:HG22	1:C:25:VAL:HG11	0.55	1.79	5	1
1:D:24:VAL:HG22	1:E:25:VAL:HG11	0.55	1.78	5	1
1:A:19:LEU:HD13	1:E:18:LEU:O	0.55	2.02	10	1
1:E:21:LEU:HD23	1:E:22:ALA:N	0.54	2.18	10	1
1:B:21:LEU:HD23	1:B:22:ALA:N	0.54	2.17	10	1
1:D:21:LEU:HD23	1:D:22:ALA:N	0.54	2.17	10	1
1:C:21:LEU:HD23	1:C:22:ALA:N	0.54	2.18	10	1
1:A:25:VAL:HG11	1:E:24:VAL:HG13	0.54	1.78	10	2
1:A:21:LEU:HD23	1:A:22:ALA:N	0.54	2.17	10	1
1:B:18:LEU:O	1:C:19:LEU:HD13	0.54	2.01	10	1
1:B:24:VAL:HG13	1:C:25:VAL:HG11	0.53	1.79	10	2
1:B:18:LEU:O	1:B:21:LEU:HD23	0.52	2.05	8	1
1:C:18:LEU:O	1:C:21:LEU:HD23	0.51	2.05	8	1
1:C:24:VAL:HG22	1:D:25:VAL:HG11	0.51	1.82	5	1
1:A:25:VAL:HG11	1:E:24:VAL:HG22	0.51	1.80	5	1
1:A:18:LEU:O	1:A:21:LEU:HD23	0.51	2.05	8	1
1:D:18:LEU:O	1:D:21:LEU:HD23	0.51	2.05	8	1
1:D:21:LEU:CB	1:E:22:ALA:HB2	0.51	2.36	8	1
1:E:18:LEU:O	1:E:21:LEU:HD23	0.50	2.06	8	1
1:B:21:LEU:CB	1:C:22:ALA:HB2	0.49	2.37	8	1
1:A:21:LEU:CB	1:B:22:ALA:HB2	0.49	2.37	8	1
1:B:24:VAL:HG11	1:C:22:ALA:HB1	0.49	1.83	2	1
1:C:21:LEU:CB	1:D:22:ALA:HB2	0.49	2.37	8	1
1:A:22:ALA:HB1	1:E:24:VAL:HG11	0.48	1.85	2	1
1:A:25:VAL:HG11	1:E:24:VAL:CG1	0.48	2.39	6	1
1:C:24:VAL:HG11	1:D:22:ALA:HB1	0.48	1.84	2	1
1:A:22:ALA:HB2	1:E:21:LEU:CB	0.48	2.38	8	1
1:D:24:VAL:HG11	1:E:22:ALA:HB1	0.48	1.84	2	1
1:B:26:PHE:O	1:B:30:THR:HG23	0.48	2.09	4	1
1:D:24:VAL:CG1	1:E:25:VAL:HG11	0.48	2.39	6	1
1:D:26:PHE:O	1:D:30:THR:HG23	0.47	2.09	4	1
1:C:26:PHE:O	1:C:30:THR:HG23	0.47	2.09	4	1
1:A:24:VAL:CG1	1:B:25:VAL:HG11	0.47	2.39	6	1
1:E:26:PHE:O	1:E:30:THR:HG23	0.47	2.09	4	1
1:B:24:VAL:CG1	1:C:25:VAL:HG11	0.47	2.40	6	1
1:A:24:VAL:HG11	1:B:22:ALA:HB1	0.47	1.85	2	1
1:A:24:VAL:HG22	1:B:25:VAL:CG1	0.47	2.40	5	1
1:D:24:VAL:HG22	1:E:25:VAL:CG1	0.47	2.40	5	1
1:A:13:ILE:HG22	1:A:17:VAL:HG23	0.46	1.87	3	1

Continued on next page...

CONFIDENTIAL VALIDATION REPORT

Continued from previous page...

Atom-1	Atom-2	Clash(Å)	Distance(Å)	Models	
				Worst	Total
1:A:26:PHE:O	1:A:30:THR:HG23	0.46	2.09	4	1
1:C:24:VAL:CG1	1:D:25:VAL:HG11	0.46	2.40	6	1
1:E:23:PHE:CE1	1:E:27:LEU:HD13	0.46	2.46	9	1
1:E:13:ILE:HG22	1:E:17:VAL:HG23	0.46	1.87	3	1
1:D:23:PHE:CE1	1:D:27:LEU:HD13	0.46	2.46	9	1
1:D:14:VAL:HG21	1:E:16:SER:OG	0.46	2.11	8	1
1:B:24:VAL:HG22	1:C:25:VAL:CG1	0.46	2.41	5	1
1:A:14:VAL:HG21	1:B:16:SER:OG	0.46	2.11	8	1
1:D:13:ILE:HG22	1:D:17:VAL:HG23	0.46	1.87	3	1
1:A:16:SER:OG	1:E:14:VAL:HG21	0.45	2.11	8	1
1:A:23:PHE:CE1	1:A:27:LEU:HD13	0.45	2.46	9	1
1:B:13:ILE:HG22	1:B:17:VAL:HG23	0.45	1.87	3	1
1:C:14:VAL:HG21	1:D:16:SER:OG	0.45	2.11	8	1
1:B:15:ASN:HA	1:B:18:LEU:HD23	0.45	1.88	2	1
1:D:15:ASN:HA	1:D:18:LEU:HD23	0.45	1.88	2	1
1:C:13:ILE:HG22	1:C:17:VAL:HG23	0.45	1.87	3	1
1:A:25:VAL:CG1	1:E:24:VAL:HG22	0.45	2.42	5	1
1:B:23:PHE:CE1	1:B:27:LEU:HD13	0.45	2.46	9	1
1:C:23:PHE:CE1	1:C:27:LEU:HD13	0.45	2.46	9	1
1:E:15:ASN:HA	1:E:18:LEU:HD23	0.45	1.88	2	1
1:B:33:ILE:HA	1:B:36:ALA:HB3	0.45	1.89	3	2
1:A:21:LEU:HD11	1:E:21:LEU:HD13	0.45	1.87	9	1
1:C:15:ASN:HA	1:C:18:LEU:HD23	0.44	1.88	2	1
1:C:28:LEU:HD11	1:D:28:LEU:HD22	0.44	1.87	2	2
1:D:23:PHE:CE1	1:D:27:LEU:HD22	0.44	2.48	9	1
1:A:28:LEU:HD22	1:E:28:LEU:HD11	0.44	1.88	2	2
1:A:28:LEU:HD11	1:B:28:LEU:HD22	0.44	1.89	2	2
1:B:31:LEU:HD22	1:C:29:VAL:CG1	0.44	2.42	8	1
1:E:24:VAL:O	1:E:28:LEU:HD23	0.44	2.13	6	1
1:E:24:VAL:O	1:E:28:LEU:HD13	0.44	2.12	7	1
1:D:31:LEU:HD22	1:E:29:VAL:CG1	0.44	2.42	8	1
1:D:24:VAL:O	1:D:28:LEU:HD13	0.44	2.13	7	1
1:C:31:LEU:HD22	1:D:29:VAL:CG1	0.44	2.43	8	1
1:C:23:PHE:CE1	1:C:27:LEU:HD22	0.44	2.48	9	1
1:C:33:ILE:HA	1:C:36:ALA:HB3	0.44	1.89	3	2
1:C:24:VAL:O	1:C:28:LEU:HD13	0.44	2.13	7	1
1:A:31:LEU:HD22	1:B:29:VAL:CG1	0.44	2.42	8	1
1:B:23:PHE:CE1	1:B:27:LEU:HD22	0.44	2.48	9	1
1:E:23:PHE:CE1	1:E:27:LEU:HD22	0.44	2.48	9	1
1:D:33:ILE:HA	1:D:36:ALA:HB3	0.44	1.89	10	2
1:D:24:VAL:O	1:D:28:LEU:HD23	0.44	2.13	6	1

Continued on next page...

CONFIDENTIAL VALIDATION REPORT

Continued from previous page...

Atom-1	Atom-2	Clash(Å)	Distance(Å)	Models	
				Worst	Total
1:B:24:VAL:O	1:B:28:LEU:HD13	0.44	2.13	7	1
1:A:33:ILE:HA	1:A:36:ALA:HB3	0.44	1.90	10	2
1:C:21:LEU:HB3	1:D:22:ALA:HB2	0.44	1.90	8	1
1:D:21:LEU:HB3	1:E:22:ALA:HB2	0.44	1.89	8	1
1:D:28:LEU:HD11	1:E:28:LEU:HD22	0.43	1.90	2	2
1:A:29:VAL:CG1	1:E:31:LEU:HD22	0.43	2.43	8	1
1:A:23:PHE:CE1	1:A:27:LEU:HD22	0.43	2.48	9	1
1:A:19:LEU:HD11	1:E:20:PHE:CE2	0.43	2.48	5	1
1:A:24:VAL:O	1:A:28:LEU:HD23	0.43	2.13	6	1
1:B:24:VAL:O	1:B:28:LEU:HD23	0.43	2.13	6	1
1:A:24:VAL:O	1:A:28:LEU:HD13	0.43	2.13	7	1
1:B:21:LEU:HD13	1:C:21:LEU:HD11	0.43	1.90	9	1
1:C:20:PHE:CE2	1:D:19:LEU:HD11	0.43	2.49	5	1
1:C:24:VAL:O	1:C:28:LEU:HD23	0.43	2.13	6	1
1:A:21:LEU:HB3	1:B:22:ALA:HB2	0.43	1.90	8	1
1:E:33:ILE:HA	1:E:36:ALA:HB3	0.42	1.89	3	2
1:A:15:ASN:HA	1:A:18:LEU:HD23	0.42	1.88	2	1
1:B:28:LEU:HD11	1:C:28:LEU:HD22	0.42	1.90	2	2
1:B:29:VAL:O	1:B:33:ILE:HG22	0.42	2.15	2	1
1:E:25:VAL:HG23	1:E:26:PHE:N	0.42	2.30	4	1
1:A:20:PHE:CE2	1:B:19:LEU:HD11	0.42	2.49	5	1
1:E:21:LEU:O	1:E:24:VAL:HG12	0.42	2.14	8	1
1:C:24:VAL:HG22	1:D:25:VAL:CG1	0.42	2.44	5	1
1:B:21:LEU:HB3	1:C:22:ALA:HB2	0.42	1.90	8	1
1:D:21:LEU:HD13	1:E:21:LEU:HD11	0.42	1.92	9	1
1:A:29:VAL:O	1:A:33:ILE:HG22	0.42	2.15	2	1
1:C:29:VAL:O	1:C:33:ILE:HG22	0.42	2.15	2	1
1:C:21:LEU:HD13	1:D:21:LEU:HD11	0.42	1.90	9	1
1:B:14:VAL:HG21	1:C:16:SER:OG	0.41	2.14	8	1
1:E:14:VAL:HG23	1:E:15:ASN:N	0.41	2.30	5	1
1:D:29:VAL:O	1:D:33:ILE:HG22	0.41	2.15	2	1
1:A:25:VAL:HG23	1:A:26:PHE:N	0.41	2.30	4	1
1:D:14:VAL:HG23	1:D:15:ASN:N	0.41	2.31	5	1
1:B:20:PHE:CE2	1:C:19:LEU:HD11	0.41	2.50	5	1
1:E:23:PHE:CE1	1:E:27:LEU:HD23	0.41	2.51	5	1
1:A:22:ALA:HB2	1:E:21:LEU:HB3	0.41	1.92	8	1
1:D:25:VAL:HG23	1:D:26:PHE:N	0.41	2.30	4	1
1:B:25:VAL:HG23	1:B:26:PHE:N	0.41	2.30	4	1
1:A:19:LEU:HD12	1:A:19:LEU:O	0.41	2.16	3	1
1:E:19:LEU:HD12	1:E:19:LEU:O	0.41	2.16	3	1
1:B:23:PHE:CE1	1:B:27:LEU:HD23	0.41	2.51	5	1

Continued on next page...

CONFIDENTIAL VALIDATION REPORT

Continued from previous page...

Atom-1	Atom-2	Clash(Å)	Distance(Å)	Models	
				Worst	Total
1:B:18:LEU:HD12	1:B:18:LEU:O	0.41	2.16	7	2
1:D:19:LEU:HD12	1:D:19:LEU:O	0.41	2.16	3	1
1:A:21:LEU:HD13	1:B:21:LEU:HD11	0.41	1.93	9	1
1:E:29:VAL:O	1:E:33:ILE:HG22	0.41	2.15	2	1
1:B:23:PHE:CG	1:B:24:VAL:N	0.41	2.89	10	1
1:B:19:LEU:O	1:B:19:LEU:HD12	0.40	2.16	3	1
1:C:25:VAL:HG23	1:C:26:PHE:N	0.40	2.30	4	1
1:C:14:VAL:HG23	1:C:15:ASN:N	0.40	2.31	5	1
1:A:18:LEU:HD12	1:A:18:LEU:O	0.40	2.16	7	1
1:E:18:LEU:HD12	1:E:18:LEU:O	0.40	2.16	7	1
1:D:23:PHE:CG	1:D:24:VAL:N	0.40	2.89	10	1
1:D:23:PHE:CE1	1:D:27:LEU:HD23	0.40	2.51	5	1
1:E:23:PHE:CG	1:E:24:VAL:N	0.40	2.89	10	1
1:D:20:PHE:CE2	1:E:19:LEU:HD11	0.40	2.51	5	1
1:C:23:PHE:CG	1:C:24:VAL:N	0.40	2.90	10	1
1:A:23:PHE:CE1	1:A:27:LEU:HD23	0.40	2.51	5	1

6.3 Torsion angles (i)

6.3.1 Protein backbone (i)

In the following table, the Percentiles column shows the percent Ramachandran outliers of the chain as a percentile score with respect to all PDB entries followed by that with respect to all NMR entries. The Analysed column shows the number of residues for which the backbone conformation was analysed and the total number of residues.

Mol	Chain	Analysed	Favoured	Allowed	Outliers	Percentiles	
1	A	25/31 (81%)	24±1 (94±4%)	1±1 (5±4%)	0±0 (1±2%)	24	71
1	B	25/31 (81%)	24±1 (95±4%)	1±1 (4±5%)	0±0 (1±2%)	24	71
1	C	25/31 (81%)	24±1 (95±4%)	1±1 (4±5%)	0±0 (1±2%)	24	71
1	D	25/31 (81%)	24±1 (94±4%)	1±1 (5±4%)	0±0 (1±2%)	24	71
1	E	25/31 (81%)	23±1 (93±4%)	2±1 (6±5%)	0±0 (1±2%)	24	71
All	All	1250/1550 (81%)	1179 (94%)	61 (5%)	10 (1%)	24	71

All 5 unique Ramachandran outliers are listed below. They are sorted by the frequency of occurrence in the ensemble.

Mol	Chain	Res	Type	Models (Total)
1	E	37	LEU	2

Continued on next page...

CONFIDENTIAL VALIDATION REPORT

Continued from previous page...

Mol	Chain	Res	Type	Models (Total)
1	D	37	LEU	2
1	C	37	LEU	2
1	B	37	LEU	2
1	A	37	LEU	2

6.3.2 Protein sidechains ⓘ

In the following table, the Percentiles column shows the percent sidechain outliers of the chain as a percentile score with respect to all PDB entries followed by that with respect to all NMR entries. The Analysed column shows the number of residues for which the sidechain conformation was analysed and the total number of residues.

Mol	Chain	Analysed	Rotameric	Outliers	Percentiles	
1	A	22/27 (81%)	14±2 (65±9%)	8±2 (35±9%)	1	10
1	B	22/27 (81%)	14±2 (65±9%)	8±2 (35±9%)	1	10
1	C	22/27 (81%)	14±2 (65±9%)	8±2 (35±9%)	1	10
1	D	22/27 (81%)	14±2 (65±9%)	8±2 (35±9%)	1	10
1	E	22/27 (81%)	14±2 (65±8%)	8±2 (35±8%)	1	9
All	All	1100/1350 (81%)	719 (65%)	381 (35%)	1	10

All 95 unique residues with a non-rotameric sidechain are listed below. They are sorted by the frequency of occurrence in the ensemble.

Mol	Chain	Res	Type	Models (Total)
1	B	23	PHE	9
1	C	23	PHE	9
1	A	23	PHE	9
1	E	23	PHE	9
1	D	23	PHE	9
1	C	20	PHE	8
1	B	20	PHE	8
1	E	20	PHE	8
1	D	20	PHE	8
1	A	20	PHE	8
1	E	24	VAL	7
1	D	24	VAL	7
1	A	24	VAL	7
1	B	24	VAL	7
1	C	24	VAL	7
1	A	30	THR	6

Continued on next page...

CONFIDENTIAL VALIDATION REPORT

Continued from previous page...

Mol	Chain	Res	Type	Models (Total)
1	B	30	THR	6
1	E	30	THR	6
1	C	30	THR	6
1	D	30	THR	6
1	C	18	LEU	5
1	A	18	LEU	5
1	E	18	LEU	5
1	E	34	LEU	5
1	D	18	LEU	5
1	B	18	LEU	5
1	B	31	LEU	4
1	D	34	LEU	4
1	D	29	VAL	4
1	A	19	LEU	4
1	B	19	LEU	4
1	B	21	LEU	4
1	A	26	PHE	4
1	B	29	VAL	4
1	E	26	PHE	4
1	A	27	LEU	4
1	D	21	LEU	4
1	D	19	LEU	4
1	B	34	LEU	4
1	E	21	LEU	4
1	C	34	LEU	4
1	A	34	LEU	4
1	D	27	LEU	4
1	A	21	LEU	4
1	C	31	LEU	4
1	B	26	PHE	4
1	E	19	LEU	4
1	C	26	PHE	4
1	D	31	LEU	4
1	E	29	VAL	4
1	D	26	PHE	4
1	C	21	LEU	4
1	A	29	VAL	4
1	B	27	LEU	4
1	C	27	LEU	4
1	E	27	LEU	4
1	C	19	LEU	4
1	E	31	LEU	4

Continued on next page...

CONFIDENTIAL VALIDATION REPORT

Continued from previous page...

Mol	Chain	Res	Type	Models (Total)
1	C	29	VAL	4
1	A	31	LEU	4
1	A	37	LEU	2
1	E	13	ILE	2
1	B	35	THR	2
1	C	13	ILE	2
1	E	35	THR	2
1	A	28	LEU	2
1	C	28	LEU	2
1	E	28	LEU	2
1	D	37	LEU	2
1	E	33	ILE	2
1	B	13	ILE	2
1	C	37	LEU	2
1	D	33	ILE	2
1	D	28	LEU	2
1	C	35	THR	2
1	C	33	ILE	2
1	B	25	VAL	2
1	E	25	VAL	2
1	D	13	ILE	2
1	B	37	LEU	2
1	E	37	LEU	2
1	A	33	ILE	2
1	B	33	ILE	2
1	C	25	VAL	2
1	D	25	VAL	2
1	A	35	THR	2
1	A	13	ILE	2
1	D	35	THR	2
1	A	25	VAL	2
1	B	28	LEU	2
1	B	15	ASN	1
1	E	15	ASN	1
1	C	15	ASN	1
1	D	15	ASN	1
1	A	15	ASN	1

6.3.3 RNA (i)

There are no RNA molecules in this entry.

CONFIDENTIAL VALIDATION REPORT

6.4 Non-standard residues in protein, DNA, RNA chains (i)

There are no non-standard protein/DNA/RNA residues in this entry.

6.5 Carbohydrates (i)

There are no monosaccharides in this entry.

6.6 Ligand geometry (i)

There are no ligands in this entry.

6.7 Other polymers (i)

There are no such molecules in this entry.

6.8 Polymer linkage issues (i)

There are no chain breaks in this entry.

CONFIDENTIAL VALIDATION REPORT

7 Chemical shift validation (i)

The completeness of assignment taking into account all chemical shift lists is 9% for the well-defined parts and 8% for the entire structure.

7.1 Chemical shift list 1

File name: D_1000251802_cs_P1.cif.V1

Chemical shift list name: *starch_output*

7.1.1 Bookkeeping (i)

The following table shows the results of parsing the chemical shift list and reports the number of nuclei with statistically unusual chemical shifts.

Total number of shifts	161
Number of shifts mapped to atoms	161
Number of unparsed shifts	0
Number of shifts with mapping errors	0
Number of shifts with mapping warnings	0
Number of shift outliers (ShiftChecker)	0

7.1.2 Chemical shift referencing (i)

The following table shows the suggested chemical shift referencing corrections.

Nucleus	# values	Correction ± precision, *ppm*	Suggested action
$^{13}C_{\alpha}$	29	-0.75 ± 0.11	Should be applied
$^{13}C_{\beta}$	28	0.42 ± 0.14	None needed (< 0.5 ppm)
$^{13}C'$	28	0.28 ± 0.15	None needed (< 0.5 ppm)
^{15}N	27	-0.12 ± 0.30	None needed (< 0.5 ppm)

7.1.3 Completeness of resonance assignments (i)

The following table shows the completeness of the chemical shift assignments for the well-defined regions of the structure. The overall completeness is 9%, i.e. 140 atoms were assigned a chemical shift out of a possible 1525. 12 out of 65 assigned methyl groups (LEU and VAL) were assigned stereospecifically.

	Total	^{1}H	^{13}C	^{15}N
Backbone	75/625 (12%)	0/250 (0%)	50/250 (20%)	25/125 (20%)
Sidechain	65/765 (8%)	0/420 (0%)	64/340 (19%)	1/5 (20%)

Continued on next page...

CONFIDENTIAL VALIDATION REPORT

Continued from previous page...

	Total	^{1}H	^{13}C	^{15}N
Aromatic	0/135 (0%)	0/75 (0%)	0/60 (0%)	0/0 (—%)
Overall	140/1525 (9%)	0/745 (0%)	114/650 (18%)	26/130 (20%)

Note: This is a solid-state NMR structure, where hydrogen atoms are typically not assigned a chemical shift value, which may lead to lower completeness of assignment measure.

The following table shows the completeness of the chemical shift assignments for the full structure. The overall completeness is 8%, i.e. 155 atoms were assigned a chemical shift out of a possible 1885. 12 out of 70 assigned methyl groups (LEU and VAL) were assigned stereospecifically.

	Total	^{1}H	^{13}C	^{15}N
Backbone	84/775 (11%)	0/310 (0%)	57/310 (18%)	27/155 (17%)
Sidechain	71/975 (7%)	0/540 (0%)	70/415 (17%)	1/20 (5%)
Aromatic	0/135 (0%)	0/75 (0%)	0/60 (0%)	0/0 (—%)
Overall	155/1885 (8%)	0/925 (0%)	127/785 (16%)	28/175 (16%)

Note: This is a solid-state NMR structure, where hydrogen atoms are typically not assigned a chemical shift value, which may lead to lower completeness of assignment measure.

7.1.4 Statistically unusual chemical shifts (i)

There are no statistically unusual chemical shifts.

7.1.5 Random Coil Index (RCI) plots (i)

The image below reports *random coil index* values for the protein chains in the structure. The height of each bar gives a probability of a given residue to be disordered, as predicted from the available chemical shifts and the amino acid sequence. A value above 0.2 is an indication of significant predicted disorder. The colour of the bar shows whether the residue is in the well-defined core (black) or in the ill-defined residue ranges (cyan), as described in section 2 on ensemble composition.

Random coil index (RCI) for chain A:

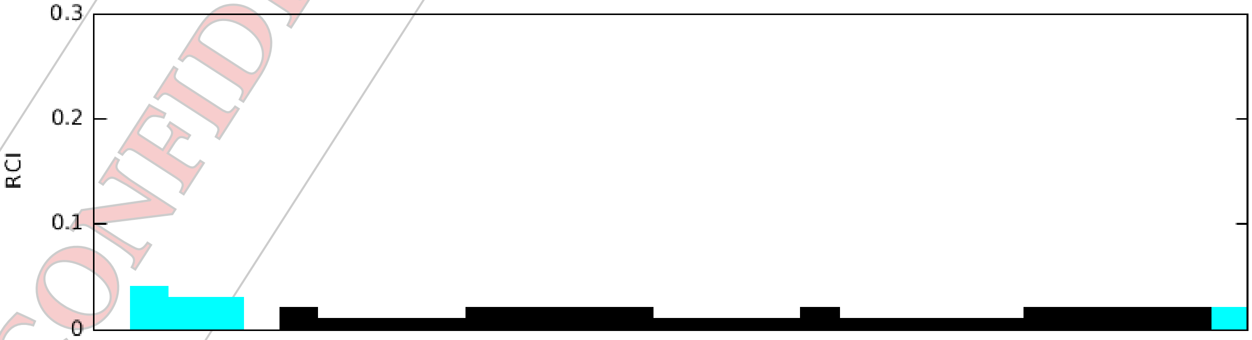

CONFIDENTIAL VALIDATION REPORT
