# Supplementary material for: Structure and Drug Binding of the SARS-CoV-2 Envelope Protein in Phospholipid Bilayers
Source: Res Sq. 2020 Sep 24:rs.3.rs-77124. Preprint. [Version 1] doi: 10.21203/rs.3.rs-77124/v1 (PMC7523133; doi:10.21203/rs.3.rs-77124/v1)
Supplement: 1 [file NIHPPrs77124v1-supplement-1.pdf]

Extended Data Figures and Tables

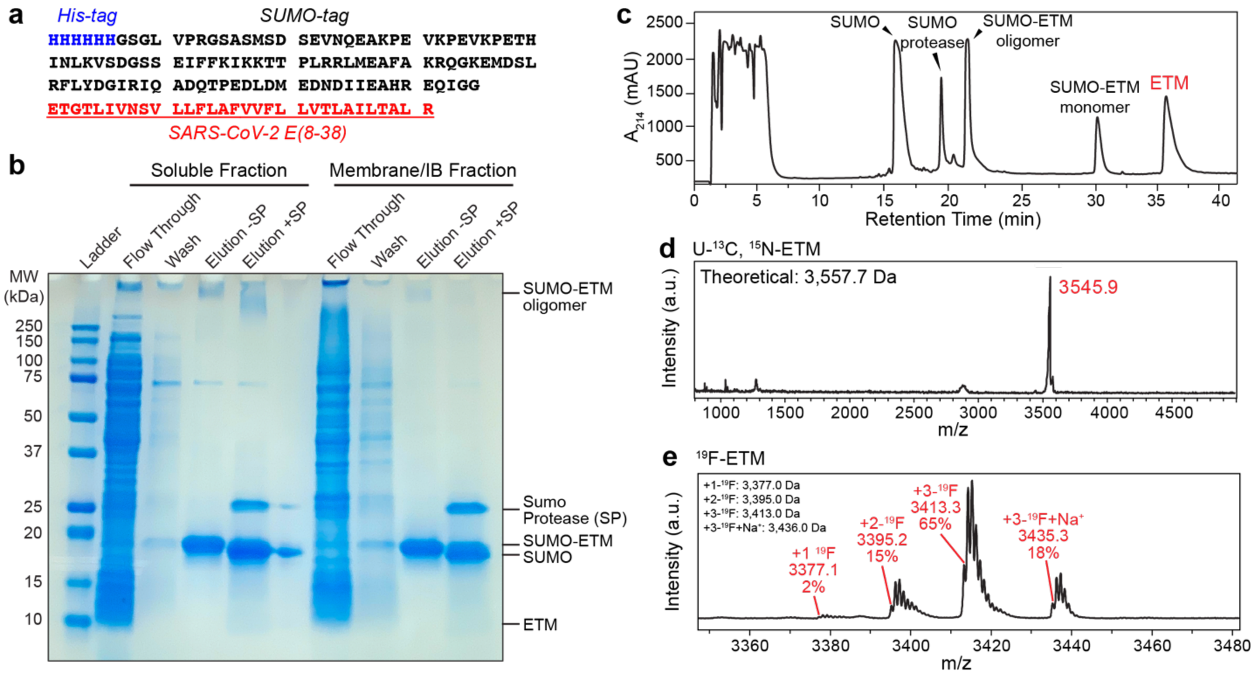

Figure S1. Cloning, purification and characterization of SARS-CoV-2 ETM. (**a**) SUMO-tagged ETM sequence. (**b**) SDS-PAGE gel showing purification of ETM by Ni^{2+}-affinity column chromatography. The flowthrough contains all soluble proteins that have low affinity for Ni^{2+}. The column was washed with 30 mM imidazole, and SUMO-ETM (18 kDa band) was eluted at >90% purity with 250 mM imidazole. High molecular-weight SUMO-ETM oligomers are visible as a minor species. ETM was cleaved from the SUMO fusion tag using SUMO protease (SP). (**c**) Preparative reverse-phase HPLC chromatogram after protease cleavage. ETM elutes at 37.5 min. (**d**) MALDI mass spectrum of purified U-^{13}C, ^{15}N labeled ETM. (**e**) MALDI mass spectrum of purified 4-^{19}F-Phe labeled ETM. The measured masses show excellent agreement with the theoretical masses. 83% of the 4-^{19}F-Phe labeled ETM monomers have all three Phe residues fluorinated, indicating a per-site labeling efficiency of 94%.

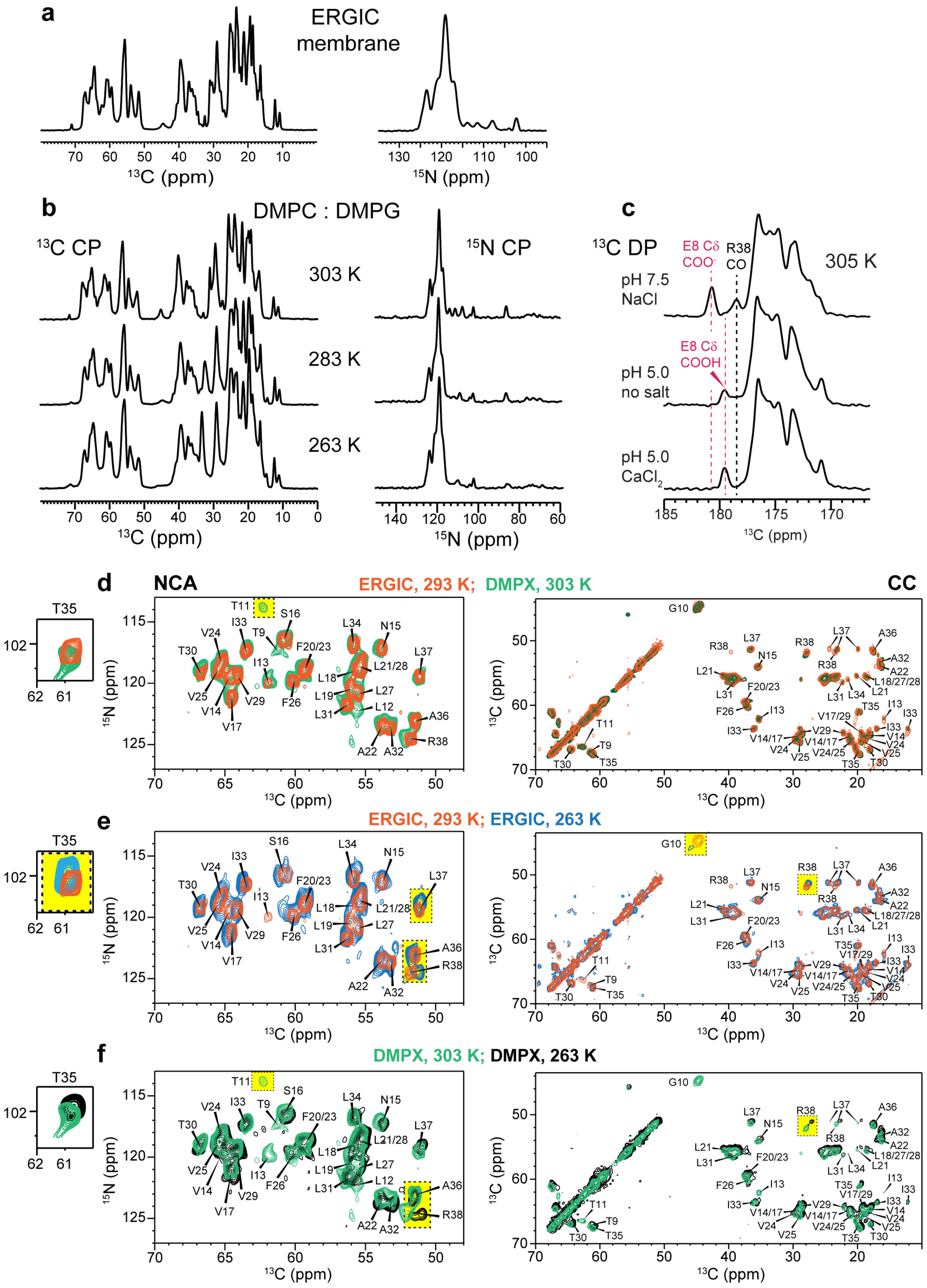

a
ERGIC membrane
13C (ppm)
15N (ppm)
b
DMPC : DMPG
13C CP
15N CP
303 K
283 K
263 K
c
13C DP
E8 Cδ COO-
R38 CO
305 K
pH 7.5 NaCl
E8 Cδ COOH
pH 5.0 no salt
pH 5.0 CaCl2
d
NCA
ERGIC, 293 K; DMPX, 303 K
CC
e
ERGIC, 293 K; ERGIC, 263 K
f
DMPX, 303 K; DMPX, 263 K
T35
15N (ppm)
13C (ppm)

Figure S2. Effects of temperature and membrane composition on ETM structure. (**a**) 1D ^{13}C and ^{15}N CP-MAS spectra of ERGIC membrane-bound ETM. The spectra show high sensitivity and resolution, indicating a well ordered and rigid structure. (**b**) 1D ^{13}C and ^{15}N CP-MAS spectra of ETM in DMPC : DMPG membranes from 303 K to 263 K. The spectral intensities and linewidths are insensitive to temperature, indicating that the protein is mostly immobilized at ambient temperature. (**c**) ^{13}C direct-polarization (DP) spectra of DMPX-bound ETM, showing the Glu8 sidechain carboxyl chemical shift change between high and low pH, indicating the protonation of this N-terminal residue at low pH. (**d-f**) 2D ^{15}N-^{13}C (left) and ^{13}C-^{13}C (right) correlation spectra of ETM at high and low temperatures and in different membranes. Yellow shaded areas highlight peaks with significant chemical shift or intensity changes. (**d**) 2D correlation spectra of ERGIC-bound ETM (*orange*) at 293 K and DMPC : DMPG bound ETM at 303 K (*green*). The chemical shifts are similar, indicating that the protein conformation is mostly unaffected by the presence of POPS, POPI and cholesterol. The T11 signal is not detected in the ERGIC membrane. (**e**) 2D correlation spectra of ERGIC-bound ETM at 293 K (*orange*) and 263 K (*blue*). Moderate chemical shift changes are observed for C-terminal residues from T35 to R38. (**f**) 2D correlation spectra of DMPC : DMPG-bound ETM at 303 K (*green*) and 263 K (*purple*). The C-terminal residues exhibit temperature-dependent chemical shifts that are similar to the ERGIC-bound peptide. The N-terminal residues of T9 to I13 do not show signals at 263 K, indicating that the N-terminus undergoes intermediate-timescale motion at low temperature. Thus, the ETM C-terminal conformation is temperature-dependent while the N-terminus is dynamic at high temperature.

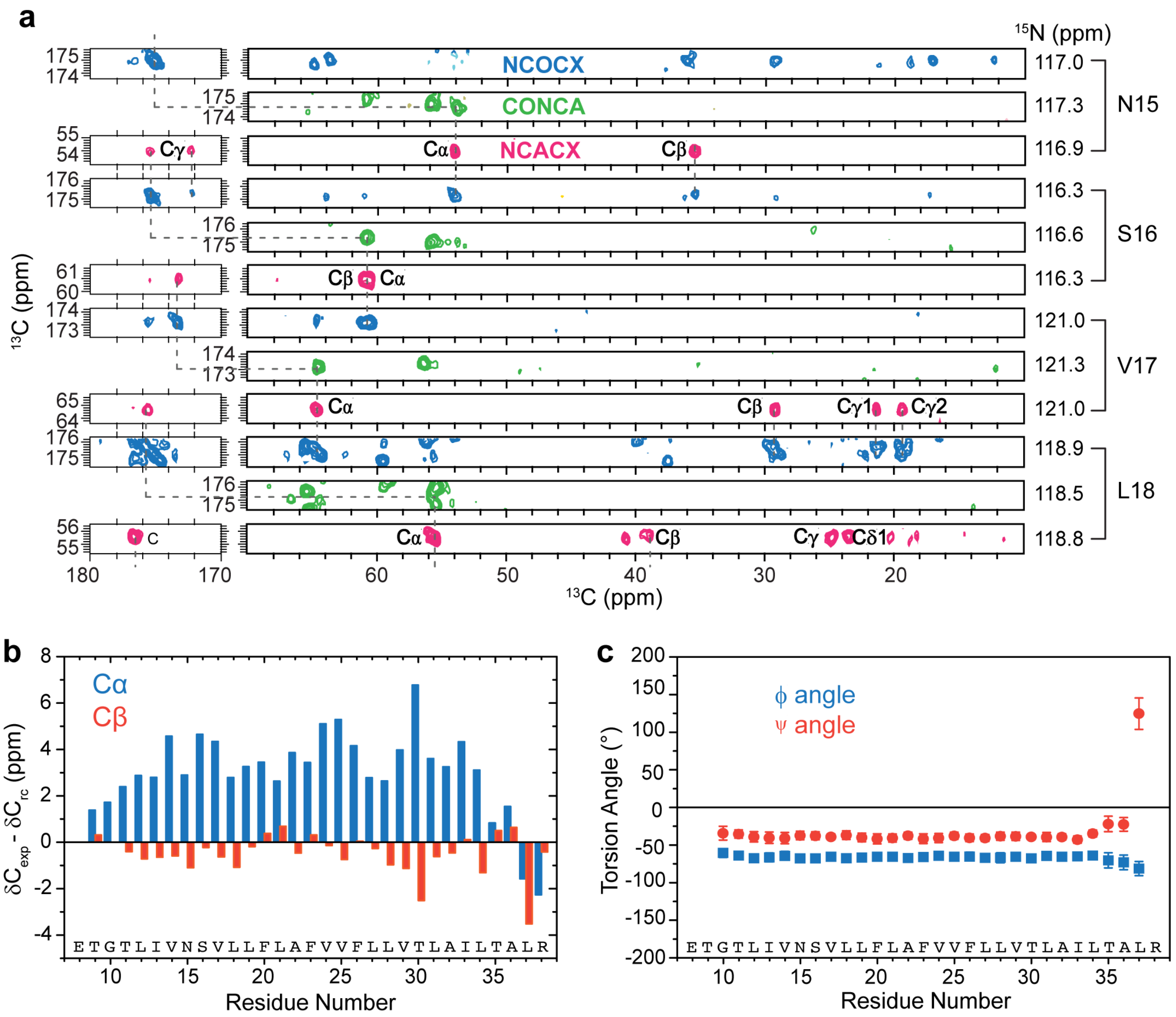

Figure S3. Chemical shift assignment and secondary structure of ETM. (**a**) Representative strips from 3D NCACX (*magenta*), CONCA (*green*) and NCOCX (*blue*) spectra of ERGIC-membrane bound ETM. These 3D spectra allow full assignment of the ^{13}C and ^{15}N chemical shifts. (**b**) Cα (*blue*) and Cβ (*orange*) secondary chemical shifts compared to the random coil chemical shifts. Most residues show positive Cα and negative Cβ secondary shifts, indicating an α-helical secondary structure. (**c**) (φ, ψ) torsion angles calculated using TALOS-N. Residues G10 to L34 show α-helical conformation.

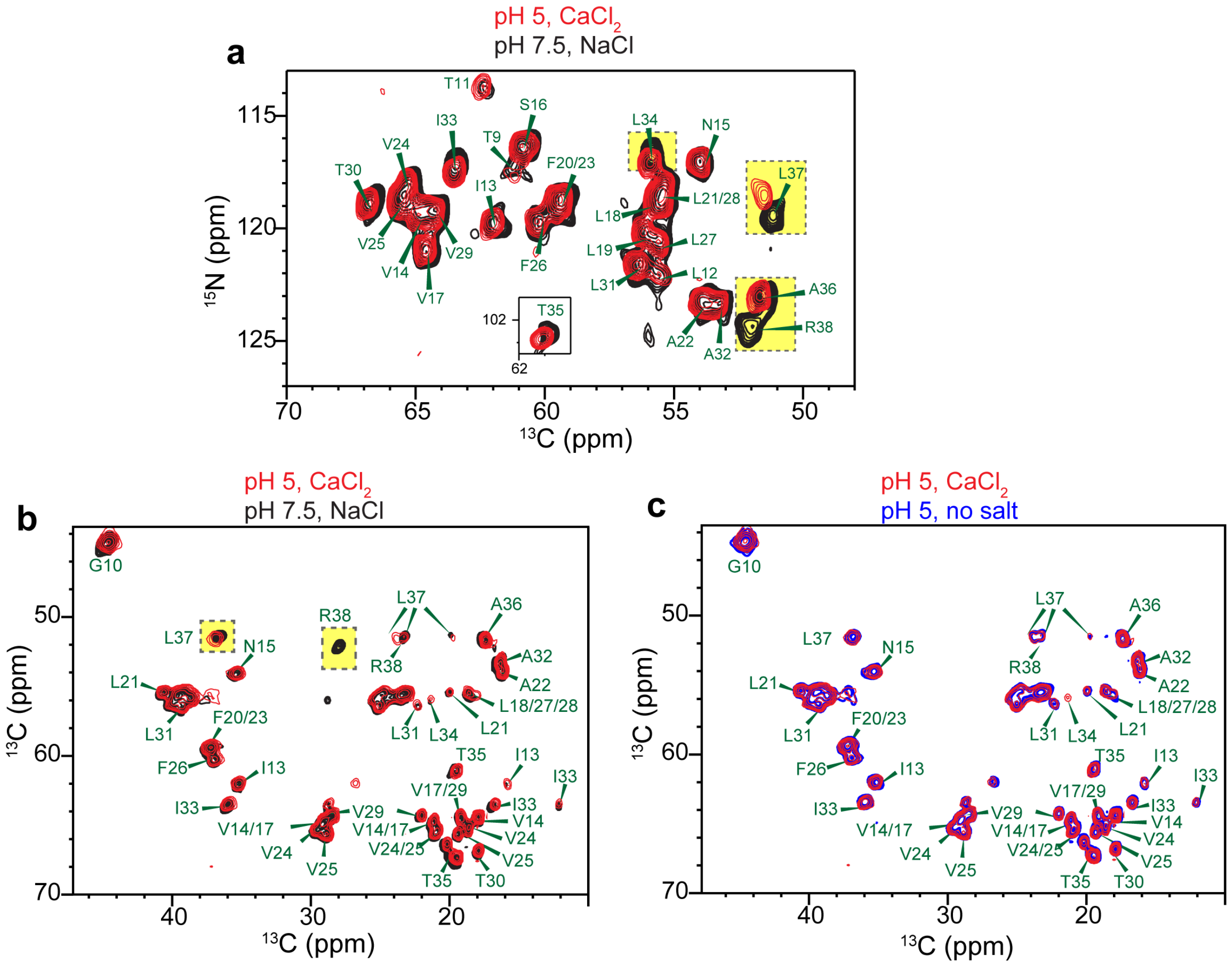

Figure S4. Effects of pH and ions on the chemical shifts of ERGIC-membrane bound ETM. Where cations are present, the ion concentration is 5 mM. (**a**) 2D ^{15}N-^{13}Cα correlation spectra of high-pH ETM with 5 mM NaCl and low-pH ETM with 5 mM $CaCl_2$. Chemical shift changes are observed for C-terminal residues such as R38, L37 and L34 (yellow highlighted regions). (**b**) 2D ^{13}C-^{13}C correlation spectra of low-pH ETM with $CaCl_2$ and high-pH ETM with NaCl. (**c**) 2D ^{13}C-^{13}C correlation spectrum of low-pH ETM with $CaCl_2$ and low-pH ETM without salt. These spectra show that chemical shift changes mainly result from pH changes.

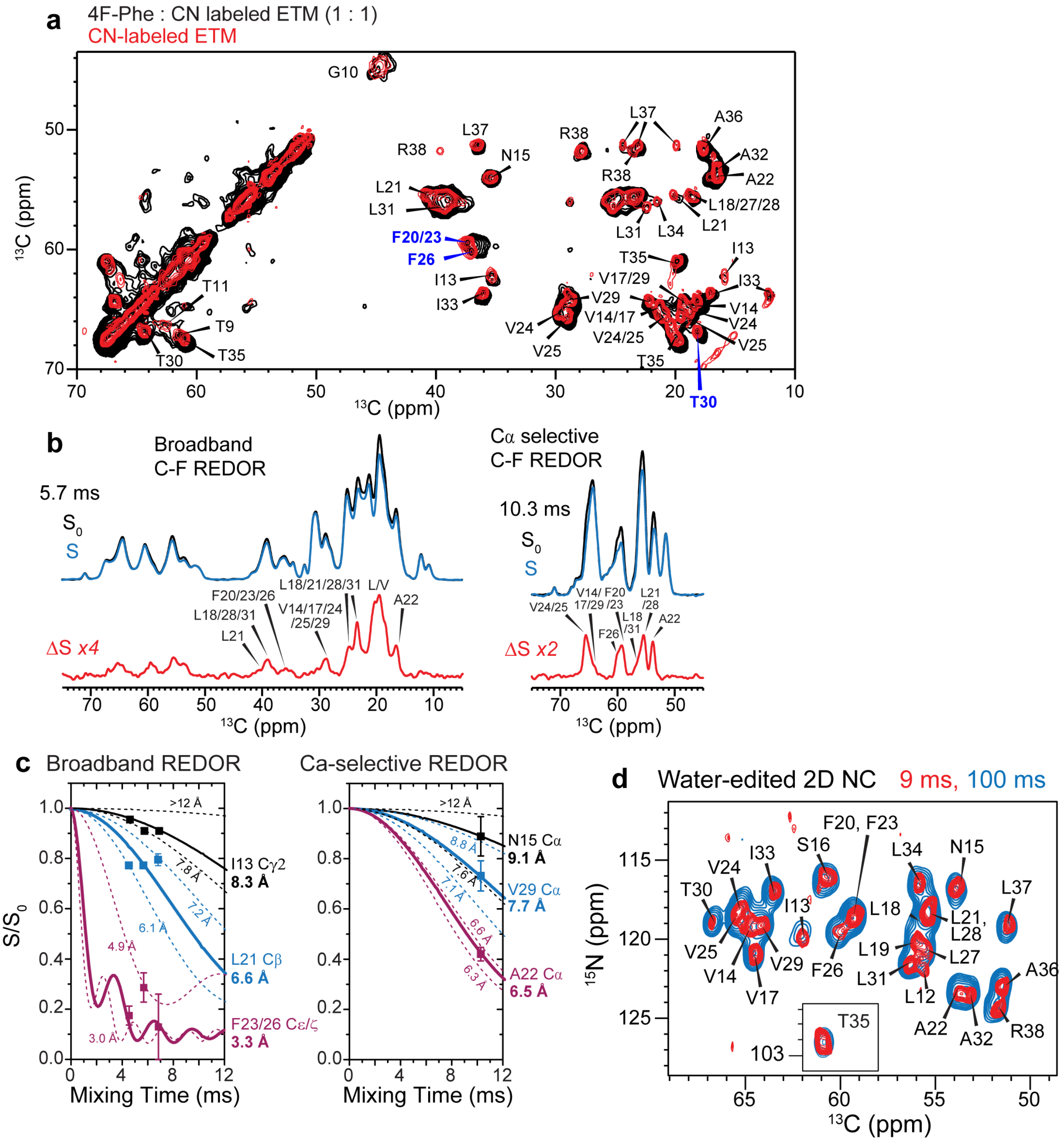

Figure S5. Additional ^{13}C-^{19}F REDOR spectra and water-edited spectra to determine the interhelical assembly of ETM. (**a**) 2D ^{13}C-^{13}C correlation spectrum of mixed 4-^{19}F-Phe labeled and U-^{13}C,^{15}N-labeled ETM (*black*). The ^{13}C chemical shifts of most residues are similar to the ^{13}C,^{15}N-labeled protein (*red*), indicating that fluorination does not significantly perturb the ETM conformation. F20/23 Cβ, F26 Cβ, and T30 Cγ2 show small chemical shift changes (*blue*) of 0.3-0.6 ppm. The spectra were measured at 293 K. (**b**) 1D ^{13}C-^{19}F REDOR control (S_0), dephased (S), and difference (ΔS) spectra. The difference peaks indicate carbons that are in close proximity to a fluorine in a neighboring helix. The broadband REDOR spectra (*left*) show both sidechain and backbone ^{13}C signals whereas the Cα-selective REDOR spectra (*right*) detect only Cα signals. (**c**) Representative ^{13}C-^{19}F REDOR dephasing curves for broadband and Cα-selective C-F REDOR spectra. The S/S_0 values have been corrected for the isotopic dilution factor (50%) and

the overlap factor. Best-fit distance curves are shown as solid lines, and lower and upper distance bounds are shown as dashed lines. (**d**) Water-edited 2D ^{15}N-$^{13}C\alpha$ correlation spectra to detect well hydrated residues. The spectra were measured at 293 K under 11.8 kHz MAS using ^{1}H-^{1}H mixing times of 9 ms (*red*) and 100 ms (*blue*).

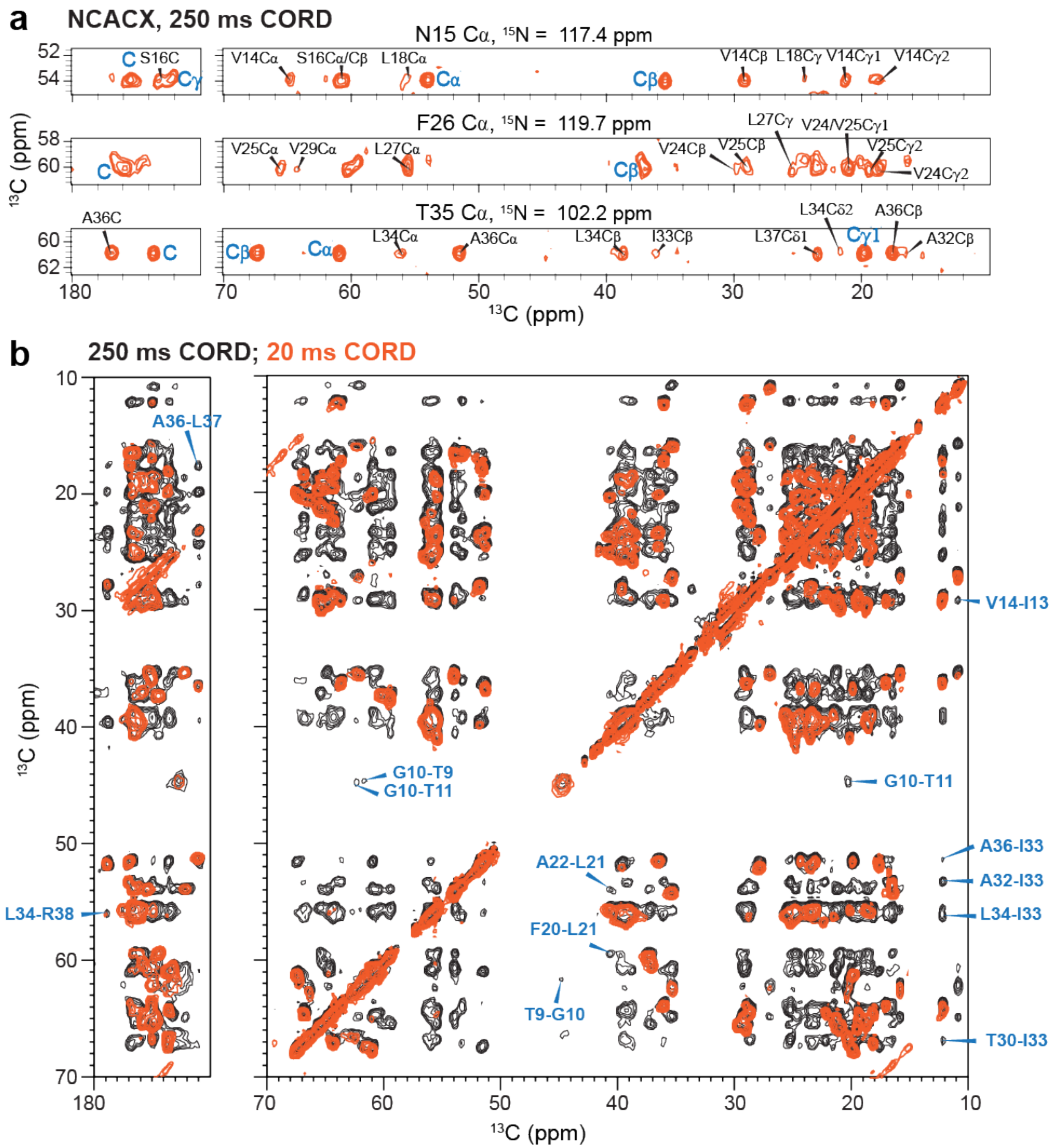

Figure S6. Inter-residue correlations obtained from 250 ms ^{13}C-^{13}C spin diffusion. (**a**) Representative strips from a well-resolved 3D NCACX spectrum recorded with 250 ms ^{13}C spin diffusion. Inter-residue cross peaks are assigned in black and intra-residue resonances are marked in blue. (**b**) Overlay of 2D ^{13}C-^{13}C correlation spectra measured with 250 ms mixing (*black*) and 20 ms (*orange*). Representative inter-residue cross peaks are assigned. All spectra were measured at 293 K under 11.8 kHz MAS.

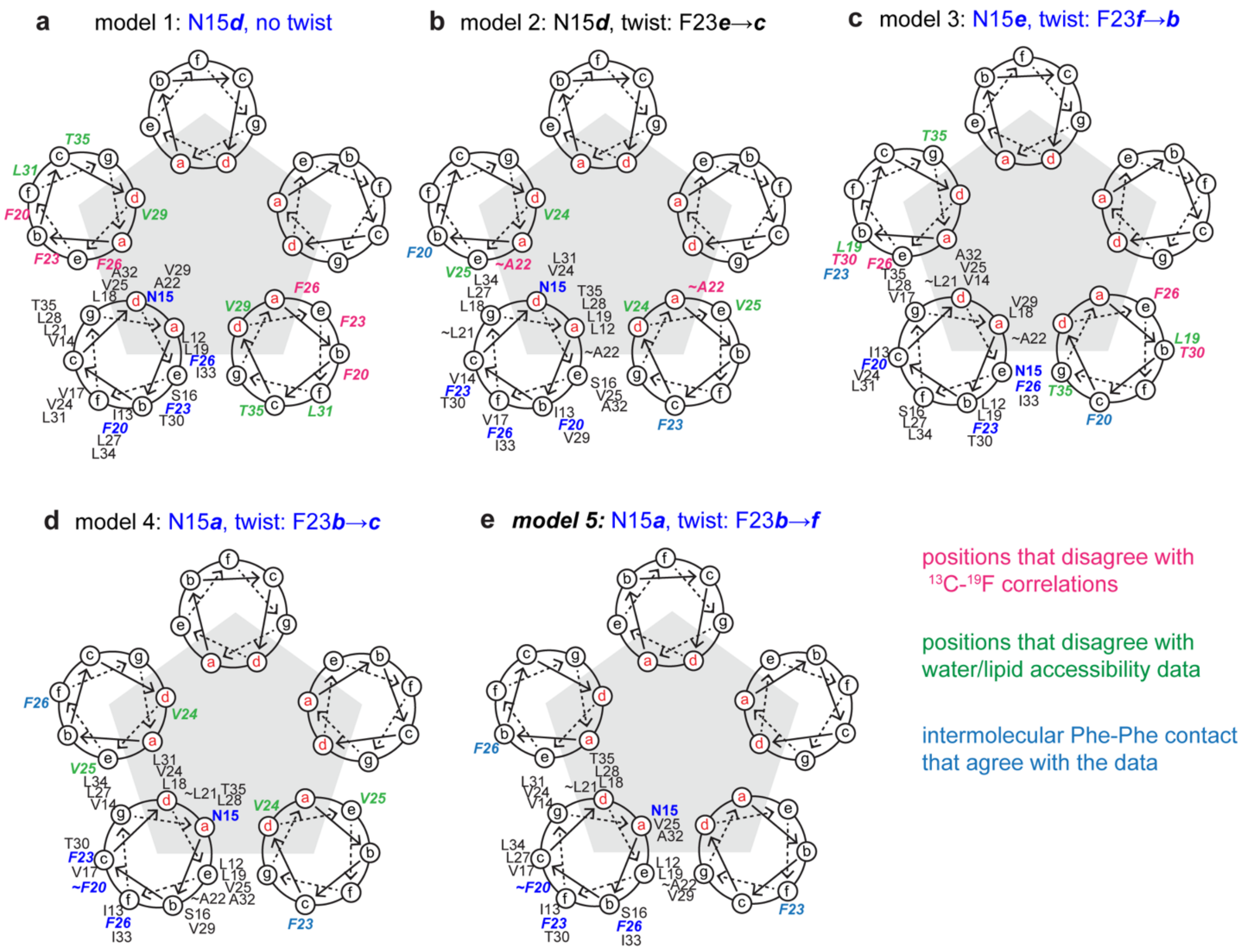

Figure S7. ETM pentameric models analyzed for structure calculation. For each model, the heptad repeat positions (*abcdefg*) of every residue from L12 to T35 is indicated on the helical wheel for one subunit. On the two neighboring helices, residue positions that violate measured ^{13}C-^{19}F correlations are shown in pink, while residue positions that violate the water/lipid accessibility data are shown in green. The positions of Phe residues that satisfy the interhelical contacts are shown in blue. (**a**) Model 1 places N15 at heptad position *d* without a twist. (**b**) Model 2 places N15 at *d* with a twist such that F23 moves from *e* to *c*. (**c**) Model 3 places N15 at *e* with a twist such that F23 moves from *f* to *b*. (**d**) Model 4 places N15 at *a* with a twist such that F23 moves from *b* to *c*. (**e**) Model 5 places N15 at *a* with a twist such that F23 moves from *b* to *f*. Model 5 does not violate any experimental data and was thus chosen to disambiguate intermolecular contacts for structure calculation.

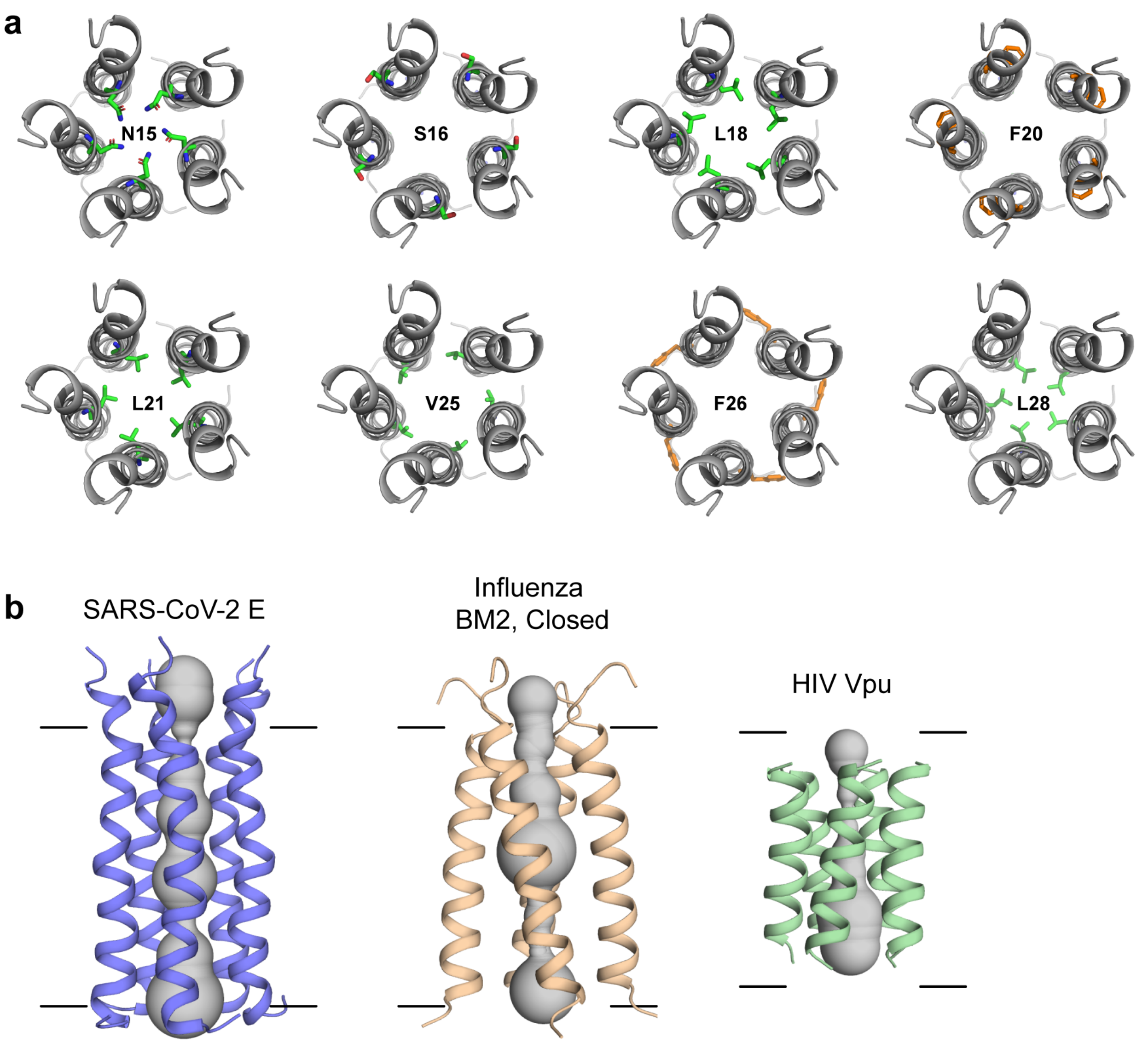

Figure S8. Membrane-bound structure of SARS-CoV-2 E TM domain compared to other viroporins. (**a**) Top views (seen from the N-terminus) of various residues in the ETM pentamer. Most residues are hydrophobic, including both pore-facing and lipid-facing residues. (**b**) Comparative HOLE plots of the pentameric ETM channel with the closed state of the tetrameric influenza BM2 proton channel (PDB:6PVR) and the pentameric HIV-1 Vpu channel (PDB: 1PI7). ETM is longer and tighter than BM2 and Vpu and exhibits a smaller helix tilt angle.

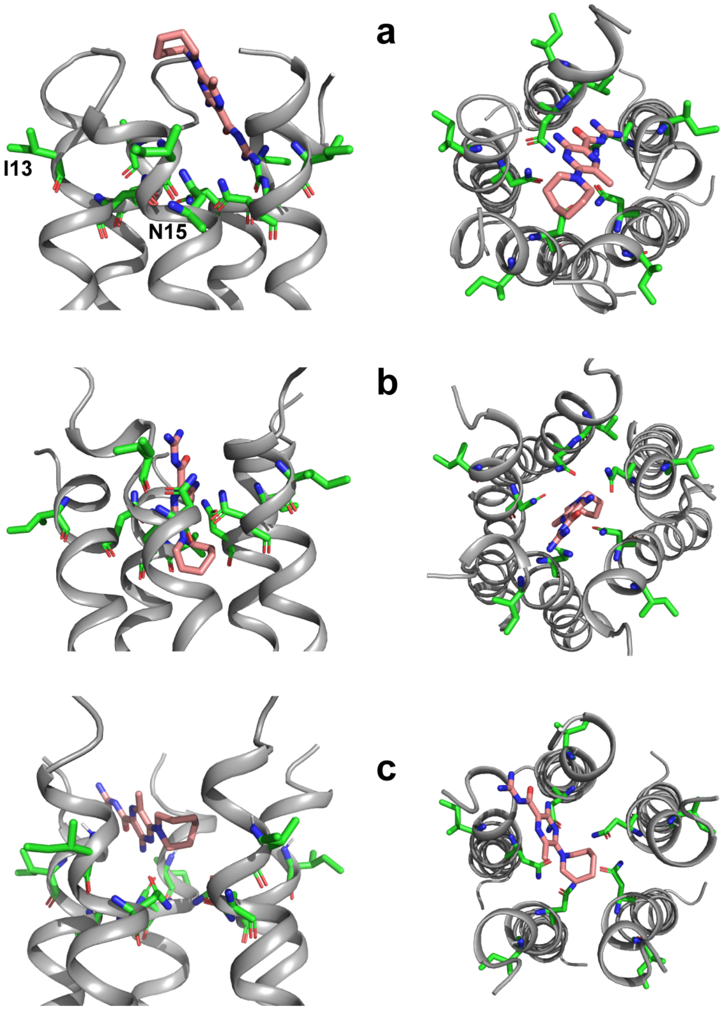

Figure S9. Additional docking poses of HMA in SARS-CoV-2 E, shown in sideview (left) and top view from the N-terminus (right). (**a**) Structure with hexamethylene up and HMA vertical, obtained from docking in DMSO. (**b**) Structure with hexamethylene down and HMA vertical, obtained from docking in DMSO. (**c**) Structure with HMA across the channel entrance, bridging two helices, obtained from docking in water. The lipid-facing I13 and pore-occluding N15 are shown in sticks to guide the eye.

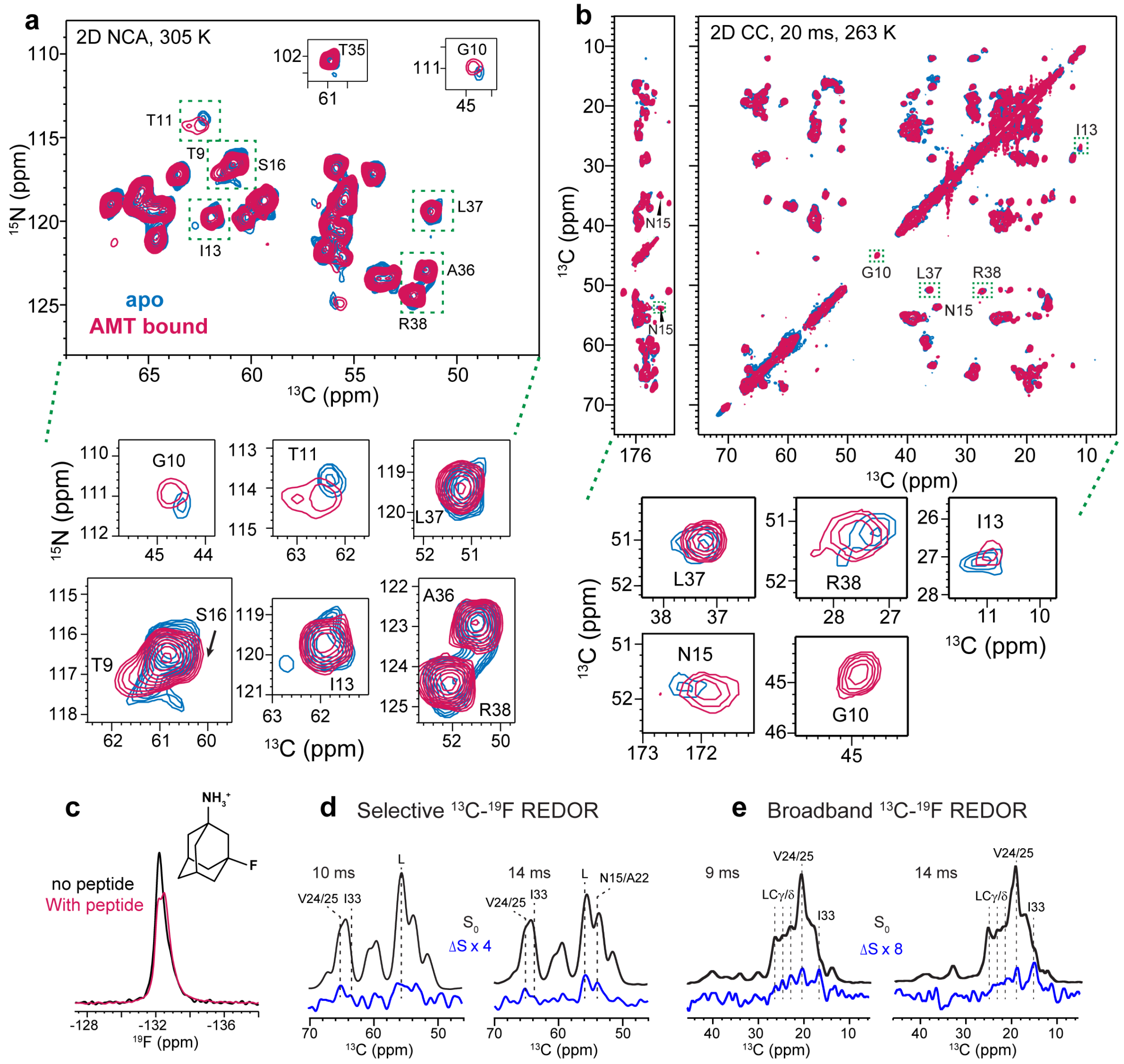

Figure S10. Effects of amantadine (Amt) binding on ETM. The peptide is reconstituted in DMPC : DMPG membranes with an Amt : ETM monomer molar ratio is 8 : 1. (**a**) 2D ^{15}N-^{13}Cα correlation spectra of apo (*blue*) and Amt-bound ETM (*magenta*). The spectra were measured at 305 K under 14 kHz MAS. Zoomed-in areas show peaks with significant CSPs. (**b**) 2D ^{13}C-^{13}C correlation spectra with 20 ms mixing of apo (*blue*) and Amt-bound ETM (*magenta*). The spectra were measured at 263 K. Zoomed-in areas shows peaks with significant CSPs. The perturbed residues are concentrated in the N- and C-termini of the protein. (**c**) 1D ^{19}F direct-polarization spectra of 3F-Amt with and without ETM in DMPX membranes. The spectra were measured at 270 K under 14 kHz MAS. (**d**) ^{13}Cα selective ^{19}F-dephased REDOR spectra of Amt-bound ETM in DMPC : DMPG membranes. The ΔS spectra show dephasing at 65.5 ppm, 63.6 ppm, 56 ppm and 54 ppm. (**e**) Broadband ^{13}C-^{19}F REDOR spectra. The ΔS spectra show ^{13}C dephasing for sidechains that belong to residues that show Cα dephasing in (**d**).

Table S1. ^{13}C and ^{15}N chemical shifts (ppm) of ERGIC-bound ETM at pH 7.5. The ^{13}C and ^{15}N chemical shift uncertainties are ±0.3 ppm and ±0.5 ppm, respectively.

Residue	Cα	Cβ	C'	N	Cγ/γ1	Cγ2	Cδ/δ1	Cδ2	Cε/ Cε1	Cε2/ ζ	Nδ
E8					33.6		181.1				
T9	61.5	67.2		117.5							
G10	44.8		172.7	110.7							
T11	62.5	66.5	173.4	113.5		20.4					
L12	55.7	39.4		121.9	25.0						
I13	62.1	35.3	175.1	119.9	27.1	15.9	10.9				
V14	64.9	29.2	174.7	119.6	21.3	18.8					
N15	53.9	35.4	175.4	117.1	172.2						107.7
S16	60.9	61.0	173.2	116.4							
V17	64.7	29.2	175.4	121.0	21.3	19.4					
L18	55.6	39.0	176.7	118.6	24.7		23.4	18.3			
L19	56.1	39.9	176.0	120.5	24.9						
F20	59.5	37.4	174.5	118.9	136.8		129.3		128.3		
L21	55.5	40.8	176.6	118.8	24.9		23.6	20.2			
A22	53.9	16.5	176.5	123.5							
F23	59.4	37.3	174.3	118.9	137.2		129.3		127.3		
V24	65.4	29.7	175.7	118.3	21.0	18.8					
V25	65.6	29.1	174.8	118.9	21.1	19.5					
F26	60.2	37.0	175.7	119.7	137.0		129.3		128.1		
L27	55.6	39.8	175.9	120.8	25.5		23.8	18.6			
L28	55.5	39.1	176.6	118.4	23.5		23.4	18.8			
V29	64.3	28.7	175.0	119.4	22.2	19.4					
T30	66.9	64.4	173.5	119.2		18.2					
L31	56.5	39.5	176.1	121.7	25.3		25.2	22.3			
A32	53.3	16.5	177.0	123.6							
I33	63.6	36.1	175.0	117.6	28.9	17.1	12.2				
L34	55.9	38.8	176.5	117.0	25.6		25.5	21.5			
T35	60.9	67.4	173.6	102.2		19.8					
A36	51.6	17.6	176.9	123.1							
L37	51.3	36.6	171.0	119.5	24.4		23.3	19.9			
R38	51.8	27.9	178.9	124.4	23.5		39.6			157.3	

Table S2. Interhelical ^{1}H-^{1}H distance restraints obtained from the NHHC spectra. The direction of the interhelical contact from the ^{15}N to the ^{13}C is indicated as 'CCW' for counter-clockwise, 'CW' for clockwise, and 'Ambig' for either of the two neighboring helices during structure calculation.

Source (^{15}N)	Sink (^{13}C)	Direction	^{1}H-^{1}H SD time (us)	Distance (Å)	Lower Error (Å)	Upper Error (Å)
L12N	T11CA	CCW	500	6.0	3.0	3.0
S16N	L18CA	CCW	500	6.0	3.0	3.0
V14N	S16CB	CW	500	6.0	3.0	3.0
A32N	L31CA	CCW	500	6.0	3.0	3.0
L21N	A22CA	CW	500	6.0	3.0	3.0
L31N	V29CA	CW	500	6.0	3.0	3.0
V29N	T30CB	CCW	1000	6.0	3.0	5.5
S16N	V14CB	CCW	500	6.0	3.0	3.0
V14N	N15CB	CW	500	6.0	3.0	3.0
L28N/V29N	T30CA	Ambig	500	6.0	3.0	3.0
V25N	L21CB	CCW	1000	6.0	3.0	5.5
A22N	F20CB/F23CB	CCW	1000	6.0	3.0	5.5
S16N	T11CG2/L21CD2	CCW	1000	6.0	3.0	5.5
N15N	V14CA/V17CA	CCW	1000	6.0	3.0	5.5
L34N	A36CA	CW	1000	6.0	3.0	5.5
I33N/L34N	T35CA	Ambig	1000	6.0	3.0	5.5
L21N	A22CB	CW	1000	6.0	3.0	5.5
L27N	V25CA	CW	1000	6.0	3.0	5.5
A22N	V17CB	CCW	1000	6.0	3.0	5.5
A32N/A36N	I33CG2	Ambig	1000	6.0	3.0	5.5
L27N	F26CA	CW	1000	6.0	3.0	5.5
L31N	L37CA	CW	1000	6.0	3.0	5.5
T30N/L37N	R38CA	Ambig	1000	6.0	3.0	5.5
L37N	T35CB	Ambig	1000	6.0	3.0	5.5
L21N/F23N	V25CA	CCW	1000	6.0	3.0	5.5
A32N	A36CA/R38CA	Ambig	1000	6.0	3.0	5.5
L34N	I33CG1	Ambig	1000	6.0	3.0	5.5
L31N	V29CB	CW	1000	6.0	3.0	5.5
L27N	V29CB	CW	1000	6.0	3.0	5.5
N15N	S16CA	CW	500	6.0	3.0	3.0
S16N	L21CB	CCW	1000	6.0	3.0	5.5
L19N	V17CA	CCW	500	6.0	3.0	3.0
N15N	L18CA	Ambig	500	6.0	3.0	3.0
S16N	L18CB	CCW	500	6.0	3.0	3.0
V17N	L18CA/L19CA	Ambig	500	6.0	3.0	3.0
L19N	S16CA	CW	500	6.0	3.0	3.0
L31N	I33CG1	CW	500	6.0	3.0	3.0
A32N	T35CG2	Ambig	500	6.0	3.0	3.0
A32N/R38N	I33CG2	Ambig	500	6.0	3.0	3.0
S16N	L18CD2	CCW	500	6.0	3.0	3.0

I33N/L34N	T35CB	Ambig	1000	6.0	3.0	5.5
L31N	A32CB	CW	1000	6.0	3.0	5.5
A32N	A32CB	Ambig	1000	6.0	3.0	5.5
A32N/R38N	A36CA	Ambig	1000	6.0	3.0	5.5
T35N	T35CG2	Ambig	1000	6.0	3.0	5.5
L31N	I33CB	CW	1000	6.0	3.0	5.5
L31N	T30CA	Ambig	1000	6.0	3.0	5.5
A32N	T35CA	Ambig	1000	6.0	3.0	5.5
A32N	T35CB	Ambig	1000	6.0	3.0	5.5
A32N/R38N	L34CG	Ambig	1000	6.0	3.0	5.5
A22N	F20C/F23C	CCW	1000	6.0	3.0	5.5
L28N	T30CG2	Ambig	1000	6.0	3.0	5.5

Table S3. Interhelical ^{13}C-^{19}F distance restraints. Positive contacts are ^{13}C sites that show significant REDOR dephasing while negative contacts are sites that do not show significant REDOR dephasing and are thus far from all ^{19}F spins. The direction of the interhelical contact from the ^{19}F spin to ^{13}C is indicated as 'CCW' for counter-clockwise, The experiments that yielded these constraints include broadband ^{13}C-^{19}F REDOR (BB), Cα-selective ^{13}C-^{19}F REDOR (Cα-sel), and water-edited and lipid-edited (Water/lipid) experiments.

Dephasing Atom (^{19}F)	Dephased Site (^{13}C)	Direction	Experiment	Distance (Å)	Lower Error (A)	Upper Error (A)	Contact Type
F20HZ	I13CA	Ambig	2D Cα-sel	12.0	4.7	38.0	negative
F20HZ	N15CA	Ambig	2D Cα-sel	9.1	2.0	40.9	negative
F20HZ	S16CA	Ambig	2D Cα-sel	12.0	4.0	38.0	negative
F20HZ	V17CA	Ambig	2D Cα-sel	7.7	2.0	2.0	positive
F26HZ	F23CA	CCW	2D Cα-sel	6.8	2.0	2.0	positive
F20HZ	L21CA	Ambig	2D Cα-sel	6.6	2.0	2.0	positive
F20HZ/F23HZ	A22CA	CW	2D Cα-sel	6.5	2.0	2.0	positive
F26HZ	V24CA	CCW	2D Cα-sel	6.7	2.0	2.0	positive
F23HZ	V25CA	CW	2D Cα-sel	6.7	2.0	2.0	positive
F23HZ	F26CA	CW	2D Cα-sel	6.5	2.6	2.0	positive
F20HZ/F23HZ/ F26HZ	L18CA/L27C A/L28CA	Ambig	2D Cα-sel	7.2	2.0	2.0	positive
F23HZ/F26HZ	V29CA	Ambig	2D Cα-sel	7.7	2.0	2.0	positive
F26HZ	T30CB	CCW	2D Cα-sel	7.1	2.0	2.0	positive
F26HZ	T30CA	Ambig	2D Cα-sel	8.5	2.0	41.5	negative
F26HZ	L31CA	Ambig	2D Cα-sel	7.5	2.0	42.5	negative
F26HZ	A32CA	Ambig	2D Cα-sel	9.5	2.0	40.5	negative
F26HZ	I33CA	Ambig	2D Cα-sel	10.9	2.7	39.1	negative
F26HZ	T35CB	Ambig	2D Cα-sel	12.0	4.8	38.0	negative
F26HZ	T35CA	Ambig	2D Cα-sel	12.0	4.5	38.0	negative
F20HZ	I13CD1	Ambig	1D BB	12.0	2.0	38.0	negative
F20HZ	I13CG2	Ambig	1D BB	8.3	2.0	41.7	negative
F20HZ/F23HZ/ F26HZ	L21CB	Ambig	1D BB	6.1	2.0	2.0	positive
F20HZ	A22CB	CW	1D BB	5.7	2.0	2.0	positive
F26HZ	T30CG2	CCW	1D BB	5.9	2.0	2.0	positive
F26HZ	I33CD1	Ambig	1D BB	12.0	2.0	38.0	negative
F26HZ	A36CA	Ambig	1D Cα-sel	12.0	2.0	38.0	negative
F26HZ	L37CA	Ambig	1D Cα-sel	12.0	2.0	38.0	negative
F26HZ	R38CA	Ambig	1D Cα-sel	12.0	2.0	38.0	negative
F26HZ	R38CB	Ambig	1D BB	12.0	3.4	38.0	negative
F26HZ	R38CZ	Ambig	1D BB	12.0	3.4	38.0	negative
F23HZ	F26CE#/F26 CZ	CW	1D BB	3.3	2.0	2.0	positive
F26HZ	L31CD2	CCW	1D BB	6.4	2.0	2.0	positive
F26HZ	F26HZ	Ambig	Water/lipid	12.0	3.0	38.0	negative
F20HZ	F20HZ	Ambig	Water/lipid	12.0	3.0	38.0	negative
F23HZ	F23HZ	Ambig	Water/lipid	12.0	3.0	38.0	negative

Table S4. XPLOR-NIH parameters for ETM structure calculations.

XPLOR-NIH Potential	Experimental Basis	Restraints per monomer	Round 1 Scale Factor	Round 2 Scale Factor	Best model energy (kcal/mol)
PosDiffPot (ncs)	Single set of chemical shifts	-	100	100	0.57
DistSymmPot	Single set of chemical shifts	-	100	100	0.17
CDIH (dihedral angles)	TALOS-N predictions	56	400	400	0.97
NOE	Intermolecular NHHC contacts	52	0.01-20	0.5-30	128.70
NOE	Intermolecular ^{13}C-^{19}F REDOR	35	0.01-20	0.5-30	
NOE	Intramolecular inter-residue ^{13}C-^{13}C contacts	196	0	0.17-10	
HBDB	α-helical TM for residues 13-19, 23-34	11	1	1	-121.52
TorsionDB	Database favored side-chain rotamers	-	0.0001-0.1	0.01-1	2771.91
BOND	Standard bond lengths	-	1	1	19.56
ANGL	Standard bond angles	-	0.4-1	0.4-1	130.89
IMPR	Standard bond geometry	-	0.1-1	0.1-1	14.41
RepelPot	Non-bonded atomic radii repulsion	-	0.004-4	0.006-6	57.33

Table S5. Detailed solid-state NMR experimental parameters for bilayer-bound ETM.

Sample	Experiment	NMR Parameters	Experimental Time
pH 7.5, ERGIC, U-^{13}C, ^{15}N-labeled ETM	2D CC short CORD, high T	B_0 = 21.1 T; $T_{bearing}$ = 293 K; ν_{MAS} = 11.8 kHz, ns = 72, τ_{rd} = 1.7 s, $t_{1,max}$ = 4.4 ms; $t_{1,inc}$ = 25.0 μs; τ_{dwell} = 6.0 μs; τ_{acq} = 10.2 ms; τ_{HC} = 0.5 ms; τ_{CORD} = 20 ms; ν_{1Hacq} = 71 kHz	14 hrs
	2D CC short CORD, low T	B_0 = 21.1 T; $T_{bearing}$ = 263 K; ν_{MAS} = 11.8 kHz, ns = 64, τ_{rd} = 1.7 s, $t_{1,max}$ = 5.0 ms; $t_{1,inc}$ = 25.0 μs; τ_{dwell} = 6.0 μs; τ_{acq} = 10.2 ms; τ_{HC} = 0.5 ms; τ_{CORD} = 20 ms; ν_{1Hacq} = 71 kHz	12 hrs
	2D CC long CORD	B_0 = 21.1 T; $T_{bearing}$ = 293 K; ν_{MAS} = 11.8 kHz, ns = 160, τ_{rd} = 1.6 s , $t_{1,max}$ = 5.0 ms; $t_{1,inc}$ = 22.1 μs; τ_{dwell} = 6.0 μs; τ_{acq} = 12.8 ms; τ_{HC} = 0.5 ms; τ_{CORD} = 250 ms; ν_{1Hacq} = 71 kHz	30 hrs
	2D NCA, high T	B_0 = 21.1 T; $T_{bearing}$ = 293 K; ν_{MAS} = 11.8 kHz, ns = 32, τ_{rd} = 2.0 s, $t_{1,max}$ = 8.9 ms; $t_{1,inc}$ = 127 μs; τ_{dwell} = 5 μs; τ_{acq} = 15.4 ms; τ_{HN} = 0.75 ms; τ_{CN} = 4 ms; $\nu_{15NspecificCP}$ = 30 kHz; $\nu_{13CspecificCP}$ = 18 kHz; $\nu_{1HspecificCP}$ = 80 kHz; ν_{1Hacq} = 71 kHz	2.5 hrs
	2D NCA, low T	B_0 = 21.1 T; $T_{bearing}$ = 263 K; ν_{MAS} = 11.8 kHz, ns = 32, τ_{rd} = 2.0 s, $t_{1,max}$ = 8.9 ms; $t_{1,inc}$ = 127 μs; τ_{dwell} = 5 μs; τ_{acq} = 15.4 ms; τ_{HN} = 0.75 ms; τ_{CN} = 4 ms; $\nu_{15NspecificCP}$ = 30 kHz; $\nu_{13CspecificCP}$ = 18 kHz; $\nu_{1HspecificCP}$ = 80 kHz; ν_{1Hacq} = 71 kHz	2.5 hrs
	3D NCACX, 28 ms CORD	B_0 = 21.1 T; $T_{bearing}$ = 293 K; ν_{MAS} = 11.8 kHz, ns = 8, τ_{rd} = 2.0 s, $t_{1,max}$ = 5.9 ms; $t_{1,inc}$ = 169.5 μs; $t_{2,max}$ = 4 ms; $t_{2,inc}$ = 100 μs; τ_{dwell} = 5 μs; τ_{acq} = 12.8 ms; τ_{HN} = 0.75 ms; τ_{CN} = 4 ms; $\nu_{15NspecificCP}$ = 30 kHz; $\nu_{13CspecificCP}$ = 18 kHz; τ_{CORD} = 28 ms; $\nu_{1HspecificCP}$ = 80 kHz; ν_{1Hacq} = 71 kHz	25 hrs
	3D NCACX, 250 ms CORD	B_0 = 21.1 T; $T_{bearing}$ = 293 K; ν_{MAS} = 11.8 kHz, ns = 24, τ_{rd} = 2.2 s, $t_{1,max}$ = 4.7 ms; $t_{1,inc}$ = 169.5 μs; $t_{2,max}$ = 3.7 ms; $t_{2,inc}$ = 100 μs; τ_{dwell} = 5 μs; τ_{acq} = 12.8 ms; τ_{HN} = 0.75 ms; τ_{CN} = 4 ms; $\nu_{15NspecificCP}$ = 30 kHz; $\nu_{13CspecificCP}$ = 18 kHz; τ_{CORD} = 250 ms; $\nu_{1HspecificCP}$ = 80 kHz; ν_{1Hacq} = 71 kHz	69 hrs
	3D NCOCX, 37 ms CORD	B_0 = 21.1 T; $T_{bearing}$ = 293 K; ν_{MAS} = 11.8 kHz, ns = 16, τ_{rd} = 2.0 s, $t_{1,max}$ = 5.9 ms; $t_{1,inc}$ = 169.5 μs; $t_{2,max}$ = 4.4 ms; $t_{2,inc}$ = 169.5 μs; τ_{dwell} = 5 μs; τ_{acq} = 12.8 ms; τ_{HN} = 0.75 ms; τ_{CN} = 4 ms; $\nu_{15NspecificCP}$ = 30 kHz; $\nu_{13CspecificCP}$ = 42 kHz; τ_{CORD} = 37 ms; $\nu_{1HspecificCP}$ = 80 kHz; ν_{1Hacq} = 71 kHz	35 hrs
	3D CONCA	B_0 = 21.1 T; T = 293 K; ν_{MAS} = 11.8 kHz, ns = 16, τ_{rd} = 2.0 s, $t_{1,max}$ = 4.1 ms; $t_{1,inc}$ = 169.5 μs; $t_{2,max}$ = 5.8 ms; $t_{2,inc}$ = 169.5 μs; τ_{dwell} =5 μs; τ_{acq} = 12.9 ms; τ_{HC} = 1 ms; τ_{CN} = 4 ms; $\nu_{15NspecificCP}$ = 30 kHz; $\nu_{13CspecificCP}$ = 42 kHz; τ_{NC} = 4 ms; $\nu_{15NspecificCP}$ = 30 kHz; $\nu_{13CspecificCP}$ = 18 kHz; $\nu_{1HspecificCP}$ = 80 kHz; ν_{1Hacq} = 71 kHz	30 hrs
	1D ^{13}C CP	B_0 = 21.1 T; ν_{MAS} = 11.8 kHz, ns = 1024, τ_{rd} = 1.5 s, τ_{dwell} = 6 μs; τ_{acq} = 12.3 ms; τ_{HC} = 0.5 ms; ν_{1Hacq} = 71 kHz	0.4 hrs
	1D ^{15}N CP	B_0 = 21.1 T; ν_{MAS} = 11.8 kHz, ns = 1024, τ_{rd} = 1.6 s, τ_{dwell} = 12 μs; τ_{acq} = 12.3 ms; τ_{HN} = 0.75 ms; ν_{1Hacq} = 71 kHz	0.4 hrs
	1D water-edited ^{13}C CP with 9 ms ^{1}H SD	B_0 = 21.1 T; $T_{bearing}$ = 293 K; ν_{MAS} = 11.8 kHz, ns = 1024, τ_{rd} = 2.0 s, τ_{dwell} = 5 μs; τ_{acq} = 12.8 ms; τ_{1Hexc} = 1.7 ms; τ_{1HSD} = 9 ms; τ_{HC} = 0.5 ms; ν_{1Hacq} = 71 kHz	0.5 hrs
	1D water-edited ^{13}C CP with 100 ms ^{1}H SD	B_0 = 21.1 T; $T_{bearing}$ = 293 K; ν_{MAS} = 11.8 kHz, ns = 1024, τ_{rd} = 2.0 s, τ_{dwell} = 5 μs; τ_{acq} = 12.8 ms; τ_{1Hexc} = 1.7 ms; τ_{1HSD} = 100 ms; τ_{HC} = 0.5 ms; ν_{1Hacq} = 71 kHz	0.5 hrs
	2D water-edited NCA with 9 ms ^{1}H SD	B_0 = 21.1 T; $T_{bearing}$ = 293 K; ν_{MAS} = 11.8 kHz, ns = 640, τ_{rd} = 2.0 s, $t_{1,max}$ = 8.5 ms; $t_{1,inc}$ = 169.5 μs; τ_{dwell} = 5 μs; τ_{acq} = 12.8 ms; τ_{1Hexc} = 1.7 ms; τ_{1HSD} = 9 ms; τ_{HN} = 0.75 ms; τ_{NC} = 4 ms; $\nu_{15NspecificCP}$ = 30 kHz; $\nu_{13CspecificCP}$ = 18 kHz; $\nu_{1HspecificCP}$ = 80 kHz; ν_{1Hacq} = 71 kHz	46 hrs

	2D water-edited NCA with 100 ms ^{1}H SD	B_0 = 21.1 T; $T_{bearing}$ = 293 K; ν_{MAS} = 11.8 kHz, ns = 384, τ_{rd} = 2.0 s, $t_{1,max}$ = 8.5 ms; $t_{1,inc}$ = 169.5 μs; τ_{dwell} = 5 μs; τ_{acq} = 12.8 ms; τ_{1Hexc} = 1.7 ms; τ_{1HSD} = 100 ms; τ_{HN} = 0.75 ms; τ_{NC} = 4 ms; $\nu_{15NspecificCP}$ = 30 kHz; $\nu_{13CspecificCP}$ = 18 kHz; ν1HspecificCP = 80 kHz; ν_{1Hacq} = 71 kHz	23 hrs
	1D lipid-edited ^{13}C CP with 10 ms ^{1}H SD	B_0 = 18.8 T; $T_{bearing}$ = 290 K; ν_{MAS} = 14 kHz, ns = 73728, τ_{rd} = 1.6 s, τ_{dwell} = 6 μs; τ_{acq} = 12.3 ms; τ_{1Hexc} = 2.86 ms; τ_{1HSD} = 10 ms; τ_{HC} = 0.5 ms; ν_{1Hacq} = 71 kHz	35 hrs
	1D lipid-edited ^{13}C CP with 100 ms ^{1}H SD	B_0 = 18.8 T; $T_{bearing}$ = 290 K; ν_{MAS} = 14 kHz, ns = 26624, τ_{rd} = 1.6 s, τ_{dwell} = 6 μs; τ_{acq} = 12.3 ms; τ_{1Hexc} = 2.86 ms; τ_{1HSD} = 100 ms; τ_{HC} = 0.5 ms; ν_{1Hacq} = 71 kHz	12 hrs
pH 7.5, ERGIC, 1 : 1 ^{15}N-labeled : ^{13}C-labeled ETM	NHHC with 0.5 ms ^{1}H SD	B_0 = 18.8 T; $T_{bearing}$ = 290 K; ν_{MAS} = 14 kHz, ns = 1792, τ_{rd} = 1.7 s, $t_{1,max}$ = 5.7 ms; $t_{1,inc}$ = 142.9 μs; τ_{dwell} = 6 μs; τ_{acq} = 10.8 ms; τ_{1HSD} = 0.5 ms; τ_{HN} = 0.75 ms; τ_{NH} = 0.75 ms; τ_{HC} = 0.5 ms; ν_{1Hacq} = 71 kHz	68 hrs
	NHHC with 1 ms ^{1}H SD	B_0 = 18.8 T; $T_{bearing}$ = 290 K; ν_{MAS} = 14 kHz, ns = 2176, τ_{rd} = 1.7 s, $t_{1,max}$ = 5.7 ms; $t_{1,inc}$ = 142.9 μs; τ_{dwell} = 6 μs; τ_{acq} = 10.8 ms; τ_{1HSD} = 1 ms; τ_{HN} = 0.75 ms; τ_{NH} = 0.75 ms; τ_{HC} = 0.5 ms; ν_{1Hacq} = 71 kHz	87 hrs
pH 7.5, ERGIC, 1 : 1, 4-^{19}F-Phe : U-^{13}C, ^{15}N ETM	Broadband 1D ^{13}C-^{19}F REDOR, 5.7 ms (S and S_0 pair)	B_0 = 14.1 T; $T_{bearing}$ = 293 K; ν_{MAS} = 14 kHz, ns = 6144, τ_{rd} = 1.6 s, τ_{dwell} = 8 μs; τ_{acq} = 16.4 ms; τ_{HC} = 0.5 ms; $\tau_{CFREDOR}$=5.7 ms; $\nu_{1HREDOR}$ = 117 kHz; ν_{1Hacq} = 71 kHz	5.5 hrs
	1D Cα-sel ^{13}C-^{19}F REDOR, 10.3 ms (S and S_0 pair)	B_0 = 14.1 T; $T_{bearing}$ = 293 K; ν_{MAS} = 14 kHz, ns = 2048, τ_{rd} = 1.8 s, τ_{dwell} = 8 μs; τ_{acq} = 16.4 ms; $\tau_{13C\alpha sel}$ = 286 μs; τ_{HC} = 0.5 ms; $\tau_{CFREDOR}$=10.3 ms; $\nu_{1HREDOR}$ = 117 kHz; ν_{1Hacq} = 71 kHz	2 hrs
	2D ^{13}C-^{13}C resolved ^{13}C-^{19}F REDOR, 10.3 ms (S and S_0 pair)	B_0 = 14.1 T; $T_{bearing}$ = 293 K; ν_{MAS} = 14 kHz, ns = 448, τ_{rd} = 1.8 s, $t_{1,max}$ = 5.4 ms; $t_{1,inc}$ = 64 μs; τ_{dwell} = 8 μs; τ_{acq} = 14.3 ms; $\tau_{13C\alpha sel}$ = 286 μs; τ_{HC} = 0.4 ms; $\tau_{CFREDOR}$=10.3 ms; $\nu_{1HREDOR}$ = 117 kHz; ν_{1Hacq} = 71 kHz	76 hrs
	2D ^{13}C-^{19}F double-quantum CP correlation	B_0 = 14.1 T; $T_{bearing}$ = 270 K; ν_{MAS} = 38 kHz, ns = 592, τ_{rd} = 2.0 s, $t_{1,max}$ = 3.7 ms; $t_{1,inc}$ = 33 μs; τ_{dwell} = 3 μs; τ_{acq} = 6.1 ms; τ_{HC} = 1 ms; τ_{CF} = 7 ms; ν_{19F-CP} = 13 kHz; ν_{13C-CP} = 25 kHz; ν_{1H-CP} = 117 kHz; ν_{1Hacq} = 71 kHz	79 hrs
pH 7.5, DMPX	1D ^{13}C CP	B_0 = 18.8 T; ν_{MAS} = 14 kHz, ns = 1024, τ_{rd} = 1.5 s, τ_{dwell} = 6 μs; τ_{acq} = 12.3 ms; τ_{HC} = 0.5 ms; ν_{1Hacq} = 71 kHz	0.4 hrs
	1D ^{13}C DP	B_0 = 18.8 T; ν_{MAS} = 14 kHz, ns = 1024, τ_{rd} = 2.0 s, τ_{dwell} = 6 μs; τ_{acq} = 12.3 ms; ν_{1Hacq} = 71 kHz	0.5 hrs
	1D ^{15}N CP	B_0 = 18.8 T; ν_{MAS} = 14 kHz, ns = 1024, τ_{rd} = 1.6 s, τ_{dwell} = 15 μs; τ_{acq} = 12 ms; τ_{HN} = 0.75 ms; ν_{1Hacq} = 71 kHz	0.4 hrs
	2D CC short CORD, high T	B_0 = 18.8 T; $T_{bearing}$ = 305 K; ν_{MAS} = 14 kHz, ns = 96, τ_{rd} = 1.6 s, $t_{1,max}$ = 6.2 ms; $t_{1,inc}$ = 25.0 μs; τ_{dwell} = 6.0 μs; τ_{acq} = 12.2 ms; τ_{HC} = 0.5 ms; τ_{CORD} = 20 ms; ν_{1Hacq} = 71 kHz	17 hrs
	2D CC short CORD, low T	B_0 = 18.8 T; $T_{bearing}$ = 263 K; ν_{MAS} = 14 kHz, ns = 96, τ_{rd} = 1.6 s, $t_{1,max}$ = 6.2 ms; $t_{1,inc}$ = 25.0 μs; τ_{dwell} = 10.0 μs; τ_{acq} = 12.2 ms; τ_{HC} = 0.5 ms; τ_{CORD} = 20 ms; ν_{1Hacq} = 71 kHz	17 hrs
	2D NC TEDOR	B_0 = 18.8 T; $T_{bearing}$ = 305 K; ν_{MAS} = 14 kHz, ns = 256, τ_{rd} = 2.0 s, $t_{1,max}$ = 10 ms; $t_{1,inc}$ = 142.9 μs; τ_{dwell} = 6 μs; τ_{acq} = 12.3 ms; τ_{HC} = 0.5 ms; τ_{NC} = 1.43 ms; ν_{1Hacq} = 71 kHz	20 hrs
	2D NC TEDOR	B_0 = 18.8 T; $T_{bearing}$ = 263 K; ν_{MAS} = 14 kHz, ns = 256, τ_{rd} = 2.0 s, $t_{1,max}$ = 10 ms; $t_{1,inc}$ = 142.9 μs; τ_{dwell} = 6 μs; τ_{acq} = 12.3 ms; τ_{HC} = 0.5 ms; τ_{NC} = 1.43 ms; ν_{1Hacq} = 71 kHz	20 hrs

pH 7.5, DMPX, HMA : ETM (4 : 1)	2D CC short CORD	B_0 = 18.8 T; $T_{bearing}$ = 305 K; ν_{MAS} = 14 kHz, ns = 96, τ_{rd} = 1.6 s, $t_{1,max}$ = 6.2 ms; $t_{1,inc}$ = 25.0 μs; τ_{dwell} = 6.0 μs; τ_{acq} = 12.2 ms; τ_{HC} = 0.5 ms; τ_{CORD} = 20 ms; ν_{1Hacq} = 71 kHz	17 hrs
	2D NC TEDOR	B_0 = 18.8 T; $T_{bearing}$ = 305 K; ν_{MAS} = 14 kHz, ns = 224, τ_{rd} = 2.0 s, $t_{1,max}$ = 10 ms; $t_{1,inc}$ = 142.9 μs; τ_{dwell} = 6 μs; τ_{acq} = 12.3 ms; τ_{HC} = 0.5 ms; τ_{NC} = 1.43 ms; ν_{1Hacq} = 71 kHz	17 hrs
pH 7.5 DMPX Amt : ETM (8 : 1)	1D ^{19}F DP	B_0 = 14.1 T; $T_{bearing}$ = 270 K; ν_{MAS} = 14 kHz, ns = 10240, τ_{rd} = 1.5 s, τ_{dwell} = 6 μs; τ_{acq} = 6.1 ms; ν_{1Hacq} = 71 kHz	4.3 hrs
	1D broadband ^{13}C-^{19}F REDOR, 9.1 ms (S and S_0 pair)	B_0 = 14.1 T; $T_{bearing}$ = 270 K; ν_{MAS} = 14 kHz, ns = 5120, τ_{rd} = 1.8 s, τ_{dwell} = 8 μs; τ_{acq} = 6.1 ms; τ_{HC} = 0.5 ms; $\tau_{CFREDOR}$=9.1 ms; $\nu_{1HREDOR}$ = 117 kHz; ν_{1Hacq} = 71 kHz	5.2 hrs
	1D broadband ^{13}C-^{19}F REDOR, 11.4 ms (S and S_0 pair)	B_0 = 14.1 T; $T_{bearing}$ = 270 K; ν_{MAS} = 14 kHz, ns = 5120, τ_{rd} = 1.8 s, τ_{dwell} = 8 μs; τ_{acq} = 6.1 ms; τ_{HC} = 0.5 ms; $\tau_{CFREDOR}$=11.4 ms; $\nu_{1HREDOR}$ = 117 kHz; ν_{1Hacq} = 71 kHz	5.2 hrs
	1D Cα-selective ^{13}C-^{19}F REDOR, 10.0 ms (S and S_0 pair)	B_0 = 14.1 T; $T_{bearing}$ = 270 K; ν_{MAS} = 14 kHz, ns = 5120, τ_{rd} = 1.8 s, τ_{dwell} = 8 μs; τ_{acq} = 6.1 ms; $\tau_{13C\alpha sel}$ = 286 μs; τ_{HC} = 0.5 ms; $\tau_{CFREDOR}$=10.0 ms; $\nu_{1HREDOR}$ = 117 kHz; ν_{1Hacq} = 71 kHz	5.2 hrs
	1D Cα-selective ^{13}C-^{19}F REDOR, 14.3 ms (S and S_0 pair)	B_0 = 14.1 T; $T_{bearing}$ = 270 K; ν_{MAS} = 14 kHz, ns = 5120, τ_{rd} = 1.8 s, τ_{dwell} = 8 μs; τ_{acq} = 6.1 ms; $\tau_{13C\alpha sel}$ = 286 μs; τ_{HC} = 0.5 ms; $\tau_{CFREDOR}$=14.3 ms; $\nu_{1HREDOR}$ = 117 kHz; ν_{1Hacq} = 71 kHz	5.2 hrs
	2D CC short CORD	B_0 = 18.8 T; $T_{bearing}$ = 263 K; ν_{MAS} = 14 kHz, ns = 96, τ_{rd} = 1.6 s, $t_{1,max}$ = 6.2 ms; $t_{1,inc}$ = 25.0 μs; τ_{dwell} = 6.0 μs; τ_{acq} = 12.2 ms; τ_{HC} = 0.5 ms; τ_{CORD} = 20 ms; ν_{1Hacq} = 71 kHz	17 hrs
	2D NC TEDOR	B_0 = 18.8 T; $T_{bearing}$ = 305 K; ν_{MAS} = 14 kHz, ns = 256, τ_{rd} = 2.0 s, $t_{1,max}$ = 10 ms; $t_{1,inc}$ = 142.9 μs; τ_{dwell} = 6 μs; τ_{acq} = 12.3 ms; τ_{HC} = 0.5 ms; τ_{NC} = 1.43 ms; ν_{1Hacq} = 71 kHz	20 hrs
pH 5.0, DMPX No Salt	2D CC short CORD	B_0 = 18.8 T; $T_{bearing}$ = 305 K; ν_{MAS} = 14 kHz, ns = 96, τ_{rd} = 1.6 s, $t_{1,max}$ = 6.2 ms; $t_{1,inc}$ = 25.0 μs; τ_{dwell} = 10.0 μs; τ_{acq} = 12.2 ms; τ_{HC} = 0.5 ms; τ_{CORD} = 20 ms; ν_{1Hacq} = 71 kHz	17 hrs
pH 5.0, DMPX 5 mM $CaCl_2$	2D CC short CORD	B_0 = 18.8 T; $T_{bearing}$ = 305 K; ν_{MAS} = 14 kHz, ns = 96, τ_{rd} = 1.6 s, $t_{1,max}$ = 6.2 ms; $t_{1,inc}$ = 25.0 μs; τ_{dwell} = 10.0 μs; τ_{acq} = 12.2 ms; τ_{HC} = 0.5 ms; τ_{CORD} = 20 ms; ν_{1Hacq} = 71 kHz	17 hrs
	2D NC TEDOR	B_0 = 18.8 T; $T_{bearing}$ = 305 K; ν_{MAS} = 14 kHz, ns = 224, τ_{rd} = 2.0 s, $t_{1,max}$ = 10 ms; $t_{1,inc}$ = 142.9 μs; τ_{dwell} = 6 μs; τ_{acq} = 12.3 ms; τ_{HC} = 0.5 ms; τ_{NC} = 1.43 ms; ν_{1Hacq} = 71 kHz	17 hrs

Definitions of symbols: B_0 = magnetic field; $T_{bearing}$ = thermocouple-reported bearing gas temperature; ν_{MAS} = MAS frequency; ns = number of scans per free induction decay; τ_{rd} = recycle delay; $t_{1,max}$ = maximum t_1 evolution time; $t_{1,inc}$ = t_1 increment; $t_{2,max}$ = maximum t_2 evolution time; $t_{2,inc}$ = t_2 increment; τ_{dwell} = dwell-time in the direct dimension; τ_{acq} = maximum acquisition time in the direct dimension; τ_{HC} = ^{1}H-^{13}C cross polarization contact time; τ_{CORD} = ^{13}C-^{13}C mixing time using CORD; ν_{1Hacq} = ^{1}H rf field strength for decoupling during acquisition; τ_{NC} = total ^{15}N-^{13}C TEDOR recoupling time; τ_{HN} = ^{1}H-^{15}N cross polarization contact time; τ_{1Hexc} = ^{1}H 90° pulse length for selective excitation of water; τ_{1HSD} = ^{1}H-^{1}H spin diffusion mixing time; $\tau_{13C\alpha sel}$ = 180° pulse length for selective inversion of Cα resonances; $\tau_{CFREDOR}$ = total ^{13}C-^{19}F REDOR recoupling time; $\nu_{1HREDOR}$ = ^{1}H rf field strength for decoupling during ^{13}C-^{19}F REDOR.
